# Design, synthesis, and anticancer evaluation of novel pyrrole–pyrazoline/chalcone hybrids: *in vitro* and computational insights into EGFR inhibition

**DOI:** 10.1039/d5md00800j

**Published:** 2026-04-27

**Authors:** Mansour S. Alturki, Marwa F. Ahmed, Abdulaziz H. Al Khzem, Mohamed S. Gomaa, Mohammad Sarafroz, Nada Tawfeeq, Mashael M. Alharbi, Abdulaziz K. Al Mouslem, Mohammed F. Aldawsari, Wajin R. Alruwili, Shah Alam Khan, Radwan El-Haggar, Atiah H. Almalki

**Affiliations:** a Department of Pharmaceutical Chemistry, College of Pharmacy, Imam Abdulrahman Bin Faisal University P. O. Box 1982 Dammam 31441 Eastern Province Kingdom of Saudi Arabia msalturki@iau.edu.sa; b Department of Pharmaceutical Chemistry, College of Pharmacy, Taif University P.O. Box 11099 Taif 21944 Saudi Arabia marwa.farg@tu.edu.sa marwafarag80@yahoo.com ahalmalki@tu.edu.sa; c Addiction and Neuroscience Research Unit, Taif University Taif 21944 Kingdom of Saudi Arabia; d Department of Chemistry, College of Science, King Faisal University Al-Ahsa 31982 Saudi Arabia; e Department of Pharmaceutical Sciences, College of Clinical Pharmacy, King Faisal University Al-Ahsa 31982 Saudi Arabia; f Department of Pharmaceutics, College of Pharmacy, Prince Sattam Bin Abdulaziz University Al-kharj-11942 Saudi Arabia; g College of Pharmacy, Imam Abdulrahman Bin Faisal University P.O. Box 1982 Dammam 31441 Saudi Arabia; h Department of Pharmaceutical Chemistry, College of Pharmacy, National University of Science and Technology PO Box 620, PC 130 Muscat Oman; i Pharmaceutical Chemistry Department, Faculty of Pharmacy, Capital (Formerly Helwan) University 11795 Ain Helwan, Cairo Egypt

## Abstract

A novel series of pyrrole–pyrazoline/chalcone hybrids were designed, synthesized and evaluated for antiproliferative activity to target epidermal growth factor receptor (EGFR) inhibition. All the synthesized compounds were evaluated in the NCI panel of 59 human cancer cell lines, where compound 6b emerged as the most active analogue. Enzyme-binding assays confirmed its potent EGFR inhibitory activity (IC_50_ = 0.225 μM), comparable to the reference inhibitor erlotinib (IC_50_ = 0.198 μM). Flow cytometry analysis of human breast cancer cells (MCF-7) showed that 6b induces significant cell arrest in the G2/M phase. Real-time polymerase chain reaction (RT-PCR) experiments further confirmed the molecular mechanisms, revealing that 6b modulated key apoptotic regulators, significantly increasing the Bax/Bcl-2 ratio and upregulating p53, BAX, and caspase-7, while concurrently suppressing Bcl-2 expression. Molecular simulation studies provide evidence for the preferential binding of 6b to the active state of EGFR, consistent with the experimental results. The synthetic strategy used to prepare the pyrrole–pyrazoline/chalcone scaffold is simple, hence providing efficient access to the title compounds whose potential can be further explored as an EGFR-targeted anticancer chemotype.

## Abstract 2

Design, synthesis, and biological evaluation of pyrazoline–pyrrole hybrids led to the identification of compound 6b as a potent EGFR inhibitor with significant antiproliferative activity against MCF-7 cells.

## Graphical abstract

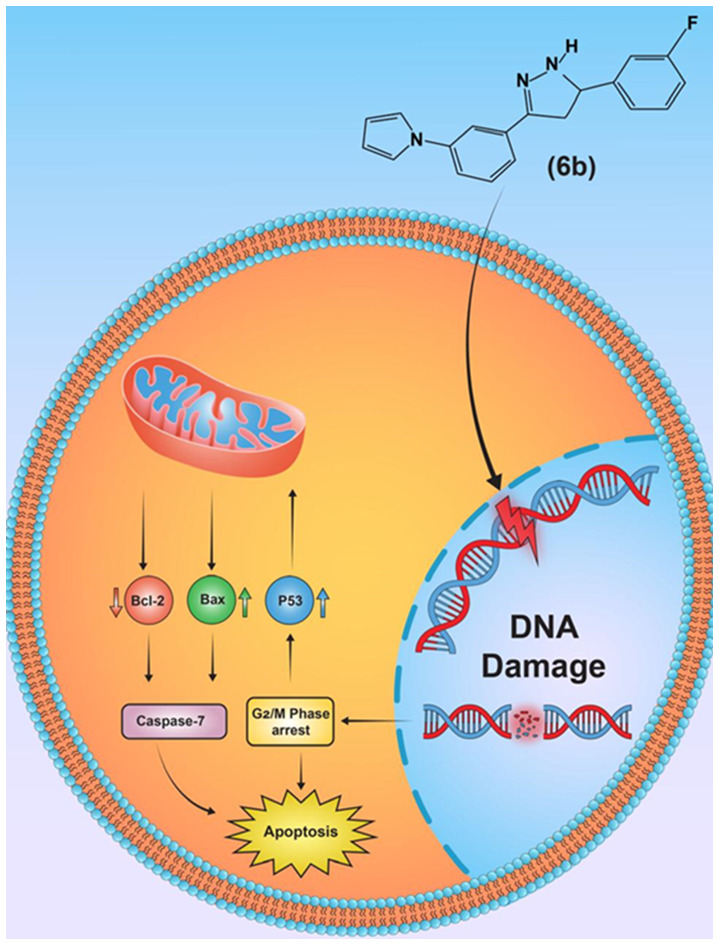

## 1. Introduction

Cancer is one of the most challenging diseases worldwide; the World Health Organization (WHO) estimated 20 million new cases and 9.7 million deaths in 2022. Data covering 185 countries and 36 cancers indicated lung, breast and colorectal cancers to be the three major cancer types in 2022.^1^ Carcinogenesis is a multifactorial process comprising several metabolic changes within a single cell. Conventional cancer treatment approaches include surgery, chemotherapy, radiation therapy, and hormonal therapy but each of these strategies poses physical, financial and psychological challenges.^2^ Amongst them, chemotherapy remains a primary approach for cancer management; however, it often fails to fully eradicate tumors and is frequently associated with significant toxicity and the development of resistance.^3^ Although, in the recent past, tremendous progress with oncological therapies has been made, severe side effects (safety and toxicity) and the development of drug resistance due to chemotherapy remain a dilemma.^3^ Given the increasing burden of cancer, there is a pressing need to develop newer chemotherapeutic agents with novel mechanisms of action for the prevention and treatment of this deadly disease.^2^

In addition, the low specificity of most chemotherapeutic agents often leads to modest efficacy or poor safety profiles. One of the most promising strategies to address these issues is the use of targeted cancer therapies.^4,5^

The epidermal growth factor receptor (EGFR), a transmembrane receptor tyrosine kinase (TK), is essential for appropriate epidermal cell development and differentiation. EGFR is a ligand-binding receptor composed of a single transmembrane domain, an extracellular domain, and a cytoplasmic region that contains a conserved protein tyrosine kinase (PTK) surrounding a series of regulatory sequences.^6,7^ The EGFR's crucial function in cell signaling pathways makes it a prominent target in cancer treatment. EGFR is a well-established target implicated in the development and prognosis of various types of cancer. EGFR overexpression is associated with poor treatment outcomes for patients with a number of human malignancies, including breast, lung, colorectal, prostate, and brain cancers,^8^ which is due to the development of resistance to hormone therapy, chemotherapy, and radiation.^9^ Therefore, this important protein has been and is still the target of various drug discovery projects in the ongoing battle with cancer. Several EGFR targeting ligands have shown clinical success and decent numbers have acquired FDA approval for the treatment of various types of cancer including among others, erlotinib and lapatinib.^10^ Accordingly, there have been a growing number of recommendations for the use of EGFR inhibitors as the preferred first-line treatment due to their superior efficacy and safety compared to conventional chemotherapy.^11,12^

**Fig. 1:**
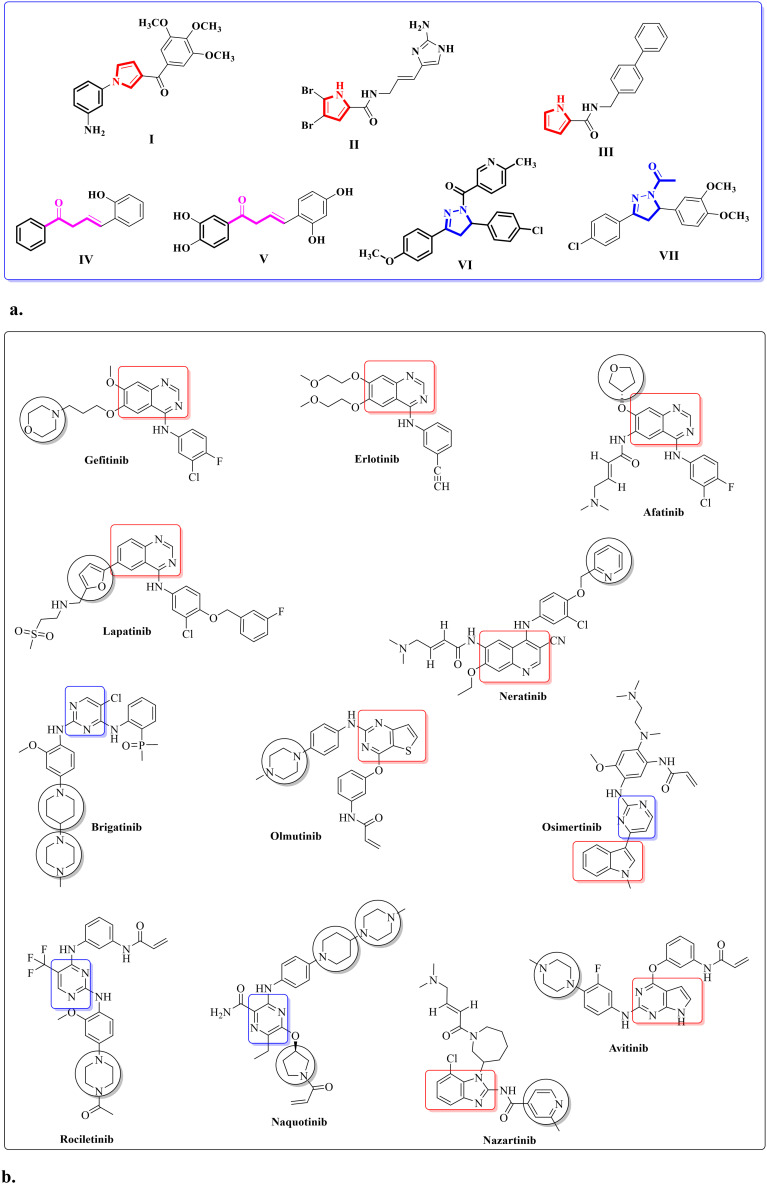
a. Structures of reported antitumor compounds containing pyrrole (I–III), chalcone (IV–V), pyrazoline (VI), and/or acetylpyrazoline (VII) moieties. b. Examples of US FDA approved small molecules as EGFR-TKIs.

The chemistry of heterocyclic compounds is one of the most prominent branches of organic chemistry. These molecules have gained a great deal of interest in recent years, both for their biological applications and their utility in human society. Pyrrole derivatives are a class of biologically active heterocyclic compounds that can be used as scaffolds for the development of antimicrobial,^13–15^ antimalarial,^16–18^ antiviral,^19–21^ and anti-inflammatory drugs.^22^ Furthermore, studies have reported that the pyrrole nucleus is an essential core capable of exhibiting potent anticancer activity^23–25^ (Fig. 1a). Chalcone chemistry has attracted considerable interest due to its simple chemistry and broad spectrum of potential biological activities.^23,25,26^ There have been several compounds (Fig. 1a) containing chalcone motifs identified as tyrosine kinases inhibitors (TKIs) of EGFR that have been demonstrated to be potent antitumor agents.^27,28^ Additionally, pyrazoline derivatives have gained popularity over the past several years due to their diverse range of biological properties.^29–37^ Potent pyrazolines and acetylpyrazoline-based derivatives against the TK of EGFR were reported as novel chemotherapeutic agents (Fig. 1a).^20,21,38–44^ The majority of US Food and Drug Administration (FDA) approved small molecule anticancer drugs contain heterocyclic rings, with nearly 60% of them in general possessing a nitrogen heterocycle.^45^ Some of the examples of US FDA approved small molecule EGFR-TKIs (Fig. 1b) containing at least one more nitrogen atom as part of their pharmacophore motif include gefitinib, erlotinib, icotinib, lapatinib, vandetanib afatinib, dacomitinib, canertinib (aminoquinazoline-based derivatives), brigatinib (aminopyrimidine derivative), neratinib, pelitinib (2nd generation 4-aminoquinoline derivatives), osimertinib (3rd generation irreversible EGFR-TKI, pyrrolopyrimidine derivative), olmutinib (thieno [3,2-*d*] pyrimidine-based drug), rociletinib (2,4-diaminopyrimidine-based compound), naquotinib, avitinib (pyrrolopyrimidine-based irreversible EGFR inhibitor), and nazartinib (aminobenzimidazole-based derivative).^46–49^

The epidermal growth factor receptor (EGFR) is a well-validated oncogenic target characterized by a conserved ATP-binding pocket within its tyrosine kinase domain (TKD). Effective EGFR inhibitors typically share key pharmacophoric features essential for high-affinity binding and kinase inhibition. Hydrogen bonding with the hinge region is a critical pharmacophoric element with the ability to form hydrogen bonds with backbone amide groups in the hinge region of EGFR, particularly involving residues such as MET769. This interaction anchors the inhibitor within the ATP-binding site and is central to inhibitory potency. Hydrophobic/aromatic interactions through aromatic rings and hydrophobic moieties enable π–π stacking and van der Waals contacts with hydrophobic pockets adjacent to the hinge region, enhancing binding affinity and specificity. Key residues involved include LEU694, VAL702, LEU764, and LEU768. Ionic and polar interactions through charged or polar groups capable of ionic–π or hydrogen bonding interactions with residues such as LYS721, GLU738, and ASP831 (part of the DFG motif) contribute to stabilizing the ligand conformation and improving selectivity.

**Fig. 2:**
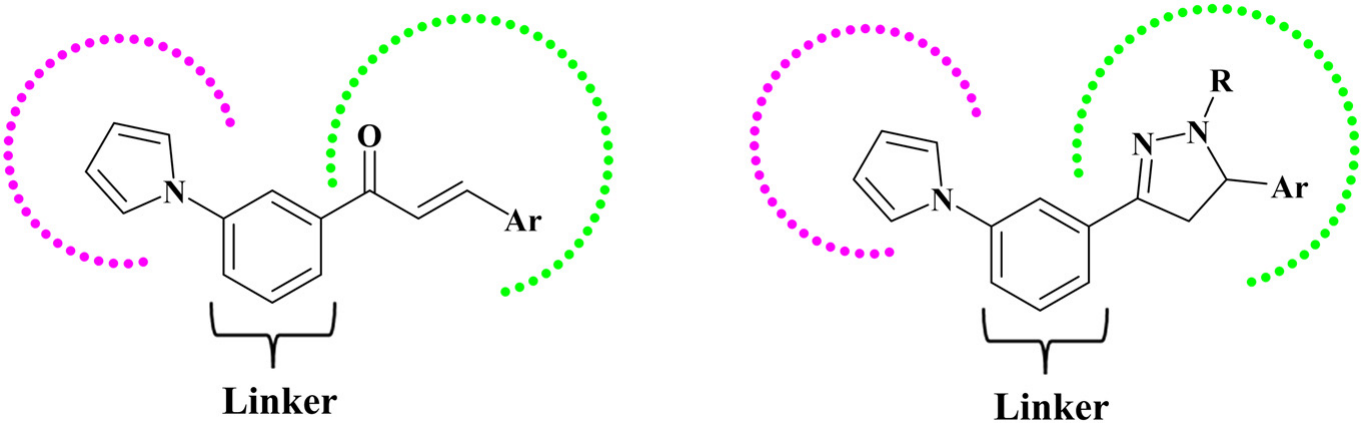
Design of the pyrrole–chalcone/pyrrole–pyrazoline hybrid compounds.

The proposed hybrid compounds strategically integrate these essential pharmacophoric elements through a rational mix-and-match approach informed by prior antitumor scaffolds. The pyrrole ring serves as an aromatic scaffold capable of engaging in π–π stacking interactions with aromatic residues lining the EGFR binding site, contributing hydrophobic contacts and enhancing binding affinity. The chalcone α,β-unsaturated ketone and pyrazoline ring systems provide hydrogen bond acceptors/donors capable of forming key interactions with the hinge region, particularly mimicking the hydrogen bonding pattern with MET769 as observed in clinically approved inhibitors like erlotinib. Acetyl groups on the pyrazoline scaffold modulate electronic properties and steric bulk, optimizing the fit within hydrophobic pockets and potentially improving metabolic stability and pharmacokinetics. Substitution on the aromatic rings with electron-withdrawing fluoro substituents may enhance metabolic stability and modulate electronic density to strengthen hydrogen bonding and van der Waals interactions within the ATP-binding pocket or that with electron-donating methyl groups may contribute hydrophobic contacts and influence the binding orientation, providing complementary activity profiles (Fig. 2). These compounds offer the distinct benefit of being easily synthesized through simple and efficient chemical procedures, facilitating effective molecular optimization and in-depth mechanistic investigations in molecular pharmacology.

These newly synthesized compounds were submitted to the National Cancer Institute (NCI) for evaluation of their antiproliferative activities. A follow-up study was conducted to determine which of the drugs is most effective as an EGFR TKI. This work also introduced the Annexin V-FITC apoptosis test. The cell cycle activity of the most effective compound was also determined to determine the possible stage at which the novel derivatives may inhibit cancer cell proliferation. Moreover, in addition to the levels of Bax and Bcl2, this investigation evaluated the levels of caspase-7. Finally, a molecular modeling approach was employed to identify binding interactions against the WT EGFR TK therapeutic target. ADMET studies and molecular dynamics simulations were conducted to evaluate the pharmacokinetic and pharmacodynamic properties of the potential EFGR inhibitors. This study aimed to develop novel EGFR inhibitors that are highly and efficiently accessible through simple, rapid and clean synthetic chemistry. This would eventually speed up the drug optimization phase and facilitate the chemical development process in the clinical trial phase.

## 2. Experimental

### 2.1. Chemistry

All reagents and solvents used in the following experiments were obtained from commercial sources. A digital melting point apparatus Electrothermal Stuart 5MP3 was utilized to measure melting points. Elemental analysis was performed on a Vario EL III elemental analyzer. The IR spectrograms of the compounds were validated using an infrared spectrophotometer (Perkin Elmer 9712). NMR spectra were obtained using Joel-Ex and Varian-Gemini NMR spectrometers for the investigation of ^1^H NMR and ^13^C NMR (500 MHz for ^1^H-NMR and 101 MHz for ^13^C-NMR). For the mass spectra, a Shimadzu GC mass spectrometer MSQp1000EX was used. High-resolution mass spectrometry (HRMS) measurements were carried out on an ACQUITY UPLC–QTOF Premier system (Waters) using ESI in positive mode. Data were acquired over *m*/*z* 100–1300 with lock-mass correction (HP-1221) to ensure mass accuracy.

#### General procedure for the synthesis of 1-(3-(1*H*-pyrrol-1-yl)phenyl)ethan-1-one (3)

A mixture of 2,5-dimethoxytetrahydrofuran 2 (0.02 mol) and 3-aminoacetophenone 1 (0.02 mol) was refluxed in glacial acetic acid (25 mL) for 3 hours, followed by cooling in ice water. The resulting mixture was then basified with a NaHCO_3_ solution. Afterwards, the separated solid was washed with water and subsequently crystallized from the methanol solution; m.p. 55–56 °C.^50^

#### General method for the preparation of 5a–d

To a mixture of 3 (0.02 mol) and different aldehydes (4a–d; 0.02 mol) in ethanol (25 mL), a solution of NaOH (20%, 7 mL) was gradually added. Following 24 hours of stirring, the mixture was poured into ice water and neutralized with HCl. The separated product was filtered, washed, and crystallized from ethanol.

#### (*E*)-1-(3-(1*H*-Pyrrol-1-yl)phenyl)-3-phenylprop-2-en-1-one (5a)

Yield 65%, m.p. 174–176 °C. Elemental analysis, calculated C_19_H_15_NO; calcd.: % C, 83.49; H, 5.53; N, 5.12. Found: % C, 83.45; H, 5.50; N, 5.14. IR: *υ*_max._/cm^−1^ 3010 (C–H aromatic), 1723 (C=O), 1590 (C=C). ^1^HNMR (DMSO-*d*_6_): *δ* 6.43 (d, 1H, *J* = 15.5 Hz, CH=), 6.62 (d, 2H, *J* = 8.4 Hz, H-pyrrole), 6.97 (d, 2H, *J* = 8.2 Hz, H-pyrrole), 7.34–7.82 (m, 9H, Ar–H, CH=) and 8.18 (s, 1H, Ar–H). ^13^C NMR (DMSO-*d*_6_): 189.4, 145.3, 142.4, 138.5, 135.3, 131.2, 129.1, 128.4, 127.6, 126.5, 124.1, 122.2, 120.6, 109.4, 107.3. MS: *m*/*z* = 273.34.

#### (*E*)-1-(3-(1*H*-Pyrrol-1-yl)phenyl)-3-(3-fluorophenyl)prop-2-en-1-one (5b)

Yield 69%, m.p. 150–152 °C. Elemental analysis, calculated C_19_H_14_FNO; calcd.: % C, 78.33; H, 4.84; N, 4.81. Found: % C, 78.39; H, 4.80; N, 4.85. IR: *υ*_max._/cm^−1^ 3000 (C–H aromatic), 1720 (C=O), 1594 (C=C). ^1^HNMR (DMSO-*d*_6_): *δ* 6.38 (d, 1H, *J* = 15.4 Hz, CH=), 6.59 (d, 2H, *J* = 8.3 Hz, H-pyrrole), 6.91 (d, 2H, *J* = 8.0 Hz, H-pyrrole), 7.48–7.83 (m, 8H, Ar–H, CH=) and 8.16 (s, 1H, Ar–H). ^13^C NMR (DMSO-*d*_6_): 189.6, 160.7, 145.2, 142.5, 138.4, 136.7, 130.8, 129.6, 128.4, 127.4, 125.3, 124.1, 121.2, 120.3, 115.7, 112.8, 110.6, 107.2. MS: *m*/*z* = 291.33.

#### (*E*)-1-(3-(1*H*-Pyrrol-1-yl)phenyl)-3-(*m*-tolyl)prop-2-en-1-one (5c)

Yield 70%, m.p. 162–164 °C. Elemental analysis, calculated C_20_H_17_NO; calcd.: % C, 83.59; H, 5.96; N, 4.87. Found: % C, 83.52; H, 5.90; N, 4.83. IR: *υ*_max._/cm^−1^ 3050 (C–H aromatic), 2950 (C–H aliphatic), 1725 (C=O), 1594 (C=C). ^1^H NMR (DMSO-*d*_6_): *δ* 2.47 (s, 3H, CH_3_), 6.33 (d, 1H, *J* = 15.0 Hz, CH=), 6.68 (d, 2H, *J* = 8.3 Hz, H-pyrrole), 6.95 (d, 2H, *J* = 8.0 Hz, H-pyrrole), 7.23–7.62 (m, 8H, Ar–H, CH=) and 8.21 (s, 1H, Ar–H). ^13^C NMR (DMSO-*d*_6_): 189.8, 145.3, 142.4, 138.5, 136.2, 132.7, 129.6, 128.6, 128.3, 127.2, 126.6, 125.4, 122.3, 119.4, 111.7, 107.1, 21.7. MS: *m*/*z* = 287.36.

#### (*E*)-1-(3-(1*H*-Pyrrol-1-yl)phenyl)-3-(4-fluorophenyl)prop-2-en-1-one (5d)

Yield 64%, m.p. 140–142 °C. Elemental analysis, calculated C_19_H_14_FNO; calcd.: % C, 78.33; H, 4.84; N, 4.81. Found: % C, 78.38; H, 4.81; N, 4.87. IR: *υ*_max._/cm^−1^ 3020 (C–H aromatic), 1721 (C=O), 1593 (C=C). ^1^H NMR (DMSO-*d*_6_): *δ* 6.25 (d, 1H, *J* = 15.5 Hz, CH=), 6.44 (d, 2H, *J* = 8.6 Hz, H-pyrrole), 6.95 (d, 2H, *J* = 8.2 Hz, H-pyrrole), 7.12–7.66 (m, 8H, Ar–H, CH=) and 8.23 (s, 1H, Ar–H). ^13^C NMR (DMSO-*d*_6_): 190.2, 160.3, 145.4, 141.1, 137.6, 135.8, 130.7, 129.5, 126.4, 125.3, 121.2, 118.7, 116.6, 111.8, 107.4. MS: *m*/*z* = 291.33.

#### General method for the preparation of 6a–c

A mixture of chalcone 5a–c (0.05 mol) and hydrazine hydrate (0.05 mol, 98 percent) in absolute ethanol (25 mL) was heated under reflux for 16 hours. After cooling the reaction, the precipitate formed was filtered off and crystallized from ethanol.

#### 3-(1*H*-Pyrrol-1-yl)phenyl-5-phenyl-4,5-dihydro-1*H*-pyrazoline (6a)

Yield 70%, m.p. 126–128 °C. Elemental analysis, calculated C_19_H_17_N_3_; calcd.: % C 79.41; H, 5.96; N, 14.62; found: % C 79.45; H, 5.90; N, 14.67. IR: *υ*_max._/cm^−1^ 3390 (NH), 3020 (C–H aromatic), 2960 (C–H aliphatic), 1595 (C=C). ^1^H NMR (DMSO-*d*_6_): *δ* 3.04 (dd, 1H, *J* = 11.2, 5.0 Hz, H5 pyrazoline), 3.23 (dd, 1H, *J* = 11.6, 5.9 Hz, H_b_4 pyrazoline), 4.97 (dd, 1H, *J* = 12.0, 5.7 Hz, H_a_4 pyrazoline), 6.36 (d, 2H, *J* = 8.6 Hz, H-pyrrole), 6.91 (d, 2H, *J* = 8.2 Hz, H-pyrrole), 7.24–7.58 (m, 9H, Ar–H) and 8.60 (s, NH, D_2_O exchangeable). ^13^C NMR (DMSO-*d*_6_): 151.5, 143.4, 142.0, 134.5, 129.3, 128.5, 126.9, 126.7, 125.2, 123.4, 120.3, 110.9, 106.2, 51.3, 44.9. MS: *m*/*z* = 287.37.

#### 3-(3-(1*H*-Pyrrol-1-yl)phenyl)-5-(3-fluorophenyl)-4,5-dihydro-1*H*-pyrazoline (6b)

Yield 67%, m.p. 129–131 °C. Elemental analysis, calculated C_19_H_16_FN_3_; calcd.: % C 74.74; H, 5.28; N, 13.76; found: % C 74.70; H, 5.22; N, 13.70. IR: *υ*_max._/cm^−1^ 3386 (NH), 3026 (CH aromatic), 2965 (C–H aliphatic), 1590 (C=C). ^1^H NMR (DMSO-*d*_6_): *δ* 3.08 (dd, 1H, *J* = 11.0, 5.1 Hz, H5 pyrazoline), 3.28 (dd, 1H, *J* = 11.4, 5.7 Hz, H_b_4 pyrazoline), 4.89 (dd, 1H, *J* = 12.1, 5.7 Hz, H_a_4 pyrazoline), 6.21 (d, 2H, *J* = 8.4 Hz, H-pyrrole), 6.97 (d, 2H, *J* = 8.0 Hz, H-pyrrole), 7.13–7.46 (m, 8H, Ar–H) and 8.52 (s, NH, D_2_O exchangeable). ^13^C NMR (DMSO-*d*_6_): 162.8, 151.9, 145.4, 142.4, 136.7, 130.1, 128.5, 126.0, 123.4, 122.4, 120.1, 115.8, 115.9, 109.9, 106.5, 51.5, 43.8. MS: *m*/*z* = 305.36. LC-HRMS: calculated for C_19_H_17_N_3_F (M + H): 306.1407, found: 306.1480.

#### 3-(3-(1*H*-Pyrrol-1-yl)phenyl)-5-(*m*-tolyl)-4,5-dihydro-1*H*-pyrazoline (6c)

Yield 71%, m.p. 154–156 °C. Elemental analysis, calculated C_20_H_19_N_3_; calcd.: % 79.70; H, 6.35; N, 13.94; found: % C 79.73; H, 6.30; N, 13.99. IR: *υ*_max._/cm^−1^ 3385 (NH), 3020 (CH aromatic), 2940 (C–H aliphatic), 1594 (C=C). ^1^H NMR (DMSO-*d*_6_): *δ* 2.30 (s, 3H, CH_3_), 3.11 (dd, 1H, *J* = 11.0, 5.2 Hz, H5 pyrazoline), 3.23 (dd, 1H, *J* = 11.4, 5.7 Hz, H_b_4 pyrazoline), 4.99 (dd, 1H, *J* = 12.1, 5.6 Hz, H_a_4 pyrazoline), 6.29 (d, 2H, *J* = 8.7 Hz, H-pyrrole), 6.96 (d, 2H, *J* = 8.5 Hz, H-pyrrole), 7.15–7.47 (m, 8H, Ar–H) and 8.59 (s, NH, D_2_O exchangeable). ^13^C NMR (DMSO-*d*_6_): 151.6, 143.4, 142.2, 138.2, 134.5, 129.8, 128.4, 127.1, 125.0, 123.6, 123.4, 122.3, 120.4, 110.8, 106.7, 51.5. 42.6, 21.8. MS: *m*/*z* = 301.39.

#### General method for the preparation of 7a–c

A mixture of chalcone 5a–c (0.01 mol) and hydrazine hydrate (0.01 mol, 98%) in glacial acetic acid (30 mL) was heated for 20 h under reflux. The reaction was cooled and the formed precipitate was filtered off and crystallized from ethanol.

#### 1-(3-(3-(1*H*-Pyrrol-1-yl)phenyl)-5-phenyl-4,5-dihydro-1*H*-pyrazoline-1-yl)ethan-1-one (7a)

Yield 70%, m.p. 119–121 °C. Elemental analysis, calculated C_21_H_19_N_3_O; calcd.: % 76.57; H, 5.81; N, 12.76; found: % C 76.51; H, 5.83; N, 12.70. IR: *υ*_max._/cm^−1^ 3020 (CH aromatic), 2930 (C–H aliphatic), 1662 (C=O). ^1^H NMR (DMSO-*d*_6_): *δ* 2.41 (s, 3H, COCH_3_), 3.72 (m, 1H, H5 pyrazoline), 3.89 (m, 1H, H_b_4 pyrazoline), 5.54 (m, 1H, H_a_4 pyrazoline), 6.21 (d, 2H, *J* = 8.6 Hz, H-pyrrole), 6.97 (d, 2H, *J* = 8.4 Hz, H-pyrrole), 7.28–7.63 (m, 9H, Ar–H). ^13^C NMR (DMSO-*d*_6_): 168.2, 151.3, 142.4, 141.6, 135.5, 129.5, 128.4, 127.0, 126.7, 125.1, 123.6, 120.3, 110.9, 106.5, 62.9, 42.9, 23.2. MS: *m*/*z* = 329.40.

#### 1-(3-(3-(1*H*-Pyrrol-1-yl)phenyl)-5-(3-fluorophenyl)-4,5-dihydro-1*H*-pyrazoline-1-yl)ethan-1-one (7b)

Yield 62%, m.p. 187–189 °C. Elemental analysis, calculated C_21_H_18_FN_3_O; calcd.: % 72.61; H, 5.22; N, 12.10; found: % C 72.58; H, 5.28; N, 12.14. IR: *υ*_max._/cm^−1^ 3025 (CH aromatic), 2950 (C–H aliphatic), 1666 (C=O). ^1^H NMR (DMSO-*d*_6_): *δ* 2.45 (s, 3H, COCH_3_), 3.67 (m, 1H, H5 pyrazoline), 3.85 (m, 1H, H_b_4 pyrazoline), 5.58 (m, 1H, H_a_4 pyrazoline), 6.25 (d, 2H, *J* = 8.5 Hz, H-pyrrole), 6.92 (d, 2H, *J* = 8.4 Hz, H-pyrrole), 7.06–7.50 (m, 8H, Ar–H). ^13^C NMR (DMSO-*d*_6_): 168.3, 162.7, 151.6, 143.6, 142.3, 130.5, 129.4, 126.2, 123.6, 122.7, 118.5, 114.9, 113.5, 111.9, 106.4, 62.5, 41.8, 23.2. MS: *m*/*z* = 347.39.

#### 1-(3-(3-(1*H*-Pyrrol-1-yl)phenyl)-5-(*m*-tolyl)-4,5-dihydro-1*H*-pyrazoline-1-yl)ethan-1-one (7c)

Yield 77%, m.p. 118–120 °C. Elemental analysis, calculated C_22_H_21_N_3_O; calcd.: % 76.94; H, 6.16; N, 12.24; found: % C 76.97; H, 6.11; N, 12.29. IR: *υ*_max._/cm^−1^ 3030 (CH aromatic), 2945 (C–H aliphatic), 1670 (C=O). ^1^H NMR (DMSO-*d*_6_): *δ* 2.24 (s, 3H, CH_3_), 2.40 (s, 3H, COCH_3_), 3.64 (m, 1H, H5 pyrazoline), 3.88 (m, 1H, H_b_4 pyrazoline), 5.51 (m, 1H, H_a_4 pyrazoline), 6.30 (d, 2H, *J* = 8.6 Hz, H-pyrrole), 6.86 (d, 2H, *J* = 8.2 Hz, H-pyrrole), 7.13–7.51 (m, 8H, Ar–H). ^13^C NMR (DMSO-*d*_6_): 168.1, 151.5, 143.4, 141.9, 138.3, 134.7, 129.1, 128.4, 127.2, 126.7, 125.0, 123.8, 123.6, 120.3, 110.8, 106.4, 66.2, 41.8, 23.4, 21.6. MS: *m*/*z* = 343.43.

### 2.2. *In vitro* cytotoxicity

Antiproliferative activity was assessed using the NCI-60 human tumor cell line panel maintained and tested by the National Cancer Institute Developmental Therapeutics Program. The panel consists of established human cancer cell lines purchased from the American Type Culture Collection (ATCC, Manassas, VA, USA). Only established commercial cell lines were used.

The NCI-60 human cancer cell lines used in this study were obtained from repositories of the National Cancer Institute Development Therapeutics Program (NCI DTP). *In vitro* tests were performed on a total of 59 human cancer cell lines using the method described by Monks *et al.*^60^ to assess the cytotoxicity of all synthesized compounds by the National Cancer Institute (NCI, USA). The antiproliferative activities of all the synthesized compounds were tested at the NCI, USA, against a panel of 59 human cancer cell lines representing nine cancer types, including leukemia, lung, colon, CNS, melanoma, ovarian, renal, prostate, and breast cancers. The assay was performed following a modified sulforhodamine B (SRB) protocol according to the method of Monks *et al.*; some major steps are mentioned here for the ease of reproducibility.^60^

Cancer cells were cultured using RPMI-1640 medium supplemented with 5% fetal bovine serum (FBS) and antibiotics as appropriate. Cells were seeded in 96-well plates, 5000–10 000 cells per well depending upon the cell line, and allowed to settle for around 24 h in a 5% CO_2_ incubator at 37 °C. The test compounds dissolved in DMSO were serially diluted in concentrations ranging from 10^−4^ to 10^−8^ M. The cells were treated in triplicate and further incubated for 48 h.

After the treatment regime, 10% cold trichloroacetic acid was added for fixing cells at 4 °C for approximately 1 h; the plates were then washed, dried, and stained with 0.4% SRB dissolved in 1% acetic acid for 30 min at room temperature. Following this, excess dye was washed off with 1% acetic acid. The dye that remained was dissolved using 10 mM Tris base (pH 10.5), and absorbance was measured at 564 nm.

### 2.3. EGFR inhibition assay

A HTRF test^51^ was used to assess the inhibitory activity of the most promising compound 6b against EGFR. Compound 6b/erlotinib was incubated with the EGFR kinase and its substrate for five minutes before adding ATP to start the enzymatic reaction. After that, the reaction was completed within 30 minutes at room temperature. An EDTA detection reagent was added after 1 hour to stop the reaction. Each concentration was tested in triplicate within an experiment, and the assay was repeated in three independent experiments (*n* = 3). IC_50_ values are expressed as the mean ± standard deviation (SD).^51^

### 2.4. Cell cycle analysis

Human breast cancer cells (MCF-7) were seeded into six-well plates at 1 × 10^5^ cells per well and allowed to attach for 24 hours at 37 °C in a humid atmosphere with 5% CO_2_ in complete culture medium supplemented with 10% FBS. After attachment, treatment with DMSO (0.1% v/v) or compound 6b (10 μM) was started. The cells were treated for 48 hours while incubation conditions remained unchanged. At the end of treatment, the cells were washed off the medium twice with PBS, followed by 70% cold ethanol addition dropwise with gentle vortexing for fixing. Fixed cells could be stored at 4 °C for at least 1 hour to maximize fixing efficacy. Before analysis, the fixed cells were washed with PBS to remove ethanol traces and stained with a PI solution in the presence of RNase A to stain the DNA specifically. The cells were incubated at 37 °C for 15 minutes to allow proper staining. Cell cycle analysis was conducted on a BD FACS Caliber flow cytometer. Data acquisition and analysis were then performed to calculate percentages of G0/G1, S, and G2/M cells. This method enabled the assessment of cell cycle arrest induced by compound 6b in MCF-7 cells. Experiments were performed in triplicate (*n* = 3).^52^

### 2.5. Apoptosis assay

To determine apoptosis induction by compound 6b, Annexin V-FITC/PI double staining was carried out using an Annexin V-FITC/PI apoptosis detection kit (BD Biosciences, San Diego, CA, USA) according to slight modifications of the manufacturer's instructions for MCF-7 cells.

MCF-7 cells were plated into T-75 flasks at a cell density of 4 × 10^6^ cells per flask and allowed to attach after overnight incubation. The following day, the cells were treated for 24 hours with compound 6b at its IC_50_ concentration of 0.225 μM in a humidified incubator with 5% CO_2_ at 37 °C. After completion of the treatment, the cells were harvested by trypsinization and pelleted by centrifugation. Around 0.5 × 10^6^ cells of all samples were then washed twice, with cold phosphate-buffered saline, and resuspended in 100 μL of 1× binding buffer. The cells were stained by adding 5 μL of Annexin V-FITC and 5 μL of propidium iodide (PI). The samples were incubated for 15 minutes at room temperature in the dark.

Following incubation, 400 μL of 1× binding buffer was added to each tube, and the samples were shortly analyzed using the BD FACS Caliber flow cytometer (BD Biosciences, San Jose, CA, USA). The data were processed to quantify the percentage of cells in the early apoptotic phase and experiments were performed in triplicate (*n* = 3).^52^

### 2.6. Caspase-7 activity

Caspase-7 activity was measured as per a previously reported method.^53^ MCF-7 cells (1 × 10^6^) at half maximal inhibitory concentrations of compound 6b for 24 h were harvested and washed with ice-cold phosphate-buffered saline (PBS). Cell pellets were lysed with 50 mM Tris–HCl (pH 7.5), 10 mM EDTA, 0.2 M NaCl, 1.5 mM PMSF, and 1% SDS. Lysates were then boiled for 10 min and centrifuged; the supernatants were stored at −20 °C. About 10 to 20 μg of protein was separated onto SDS gels and subjected to nitrocellulose membranes. Membranes were subjected to anti-caspase-7 antibodies and secondary antibodies, and chemiluminescence detection was done. The caspase-7 was compared against untreated controls.

### 2.7. Measurement of p53, BAX, and Bcl-2 expression

Levels of tumor suppressor gene p53, anti-apoptotic marker Bcl-2, and apoptotic marker BAX were evaluated, as previously reported.^40^ MCF-7 cells that were treated with compound 6b were determined by using an iScriptTM One-Step RT-PCR kit with SYBR® Green (Bio-Rad, USA). After the RNA was extracted and quantified, 1 μg of total RNA was used in each 20 μL reaction. The RT-PCR conditions were as follows: reverse transcription at 50 °C (10 min), initial denaturation at 95 °C (5 min), and 40 cycles warming up at 95 °C (10 s) and cooling down at 60 °C (30 s).

### 2.8. DNA fragmentation analysis

Using salting-out DNA isolates, agarose gel electrophoresis was performed to examine fragmentation of genomic DNA;^40^ MCF-7 cells were therefore grown with and without compound 6b. After the cells had been washed and counted, they were subjected to DNA extraction. To prepare the TE buffer, DNA samples were first meticulously resuspended. The DNA laddering was examined using an ultraviolet transilluminator and ethidium bromide staining after the material was run over a 1.5 percent TAE agarose gel.

### 2.9. Molecular modeling

#### 2.9.1. Computational tools

Computational simulations were carried out using Maestro 13.6 (Schrödinger 2023-2 version).^54^

#### 2.9.2. Crystal structures

The X-ray crystallographic structure of EGFR (active state) with erlotinib was obtained from the RCSB protein data bank (PDB ID: 1M17) with a resolution of 1.51 Å,^55^ and EGFR (inactive state) with adenylyl-imidodiphosphate (AMP-PNP) (PDB: 2GS7) with a resolution of 2.60 Å.^56^

#### 2.9.3. Protein preparation

All crystal structures were prepared using Schrödinger Maestro's protein preparation wizard tool. Structure preparation and minimization were done at pH 7.4 with corrected ionization states, polar hydrogens were added, and non-essential water molecules were removed. The entire structure was minimized and optimized with the OPLS3 force field to optimize protein energies and to remove any steric hindrance, and the default value for root mean square deviation (RMSD) of 0.30 Å for non-hydrogen atoms was used.^57^

#### 2.9.4. Ligand library preparation

Maestro Ligprep was used to prepare ligands. The structures downloaded in SDF format were converted to Maestro's 3D format. Epik was utilized to determine the optimal chirality and ionization states at a physiological pH of 7.4 ± 2.0. During this stage, several treatments were applied to the structures. Finally, the geometries were optimized using the OPLS3 force field. These conformations were used as the initial input structures for docking.^58^

#### 2.9.5. Binding pocket determination and validation of molecular docking

In Schrodinger's Maestro, the binding pocket was identified by the workspace co-crystallized inhibitor, erlotinib, and AMP-PNP. A docking grid was created using the Glide software. This grid was identified by choosing the ligand-binding pocket from the crystal structure that was co-crystallized with the inhibitor. To ensure accuracy, the co-crystallized inhibitor was redocked using the same protocol order, and the docking poses and interactions were verified using Maestro structure superimposition and RMSD calculations of the alignment.^59–61^ The receptor grids were generated using a 1.00 van der Waals (vdw) radius scaling factor and a 0.25 partial charge cutoff, and they were centered on the bound inhibitor. The binding site was enclosed within the grid box using default parameters and without constraints. Finally, the docking process was repeated and verified using three screening settings.

#### 2.9.6. Standard molecular docking (rigid)

The ligand was docked using the Glide tool without using any constraints and a 0.80 van der Waals (vdw) radius scaling factor and a 0.15 partial charge cut-off; the flexibility of the ligands was considered while the protein was considered as a rigid structure, and all other parameters were left at the default settings. GlideScore implemented in Glide was used to estimate binding affinity and rank ligands. The Pose Rank was used to select the best-docked pose for each ligand. The compounds were then analyzed in detail based on binding scores and a detailed study of all binding interactions.^62^

#### 2.9.7. Induced-fit docking (IFD) (flexible)

Induced-fit docking (IFD) is a method for modeling the conformational changes induced by ligand binding developed by Schrodinger.^63^ This protocol models induced-fit docking of one or more ligands using the steps also reported.^64^ Using the IFD tool in Maestro, the initial docking of each ligand was performed using a softened potential (van der Waals radius scaling) and flexible conformational sampling. Then, a side-chain prediction within a given distance of any ligand pose was performed. Subsequently, minimization of the same set of residues and the ligand for each protein/ligand complex pose was performed. Finally, the good binding pose was predicted based on the IFD score.

#### 2.9.8. Molecular mechanics-based re-scoring

The molecular mechanics generalized Born surface area (MM/GBSA) docking was performed to re-score the binding complexes for accurate affinity prediction.^65^ The increased accuracy of MM/GBSA is mainly given by its ability to allow flexibility of both the ligand and its receptor, creating important physiological relevance.^66^ Consequently, the intensive MM–GBSA simulation was used to rank the binding affinity of 6b against the active and inactive states of the EFGR target. To perform this task, the initial XP complexes of 6b and erlotinib were subjected to the MM/GBSA docking tool in Maestro. To impart the flexibility of both the ligand and receptor target, the distance of 6b or erlotinib and EGFR was adjusted to 5 Å. The combination of the VGSB solvation model and OPLS3 force field was utilized for this simulation.^67^

#### 2.9.9. Molecular dynamics (MD) simulations

On top of the motion effect, molecular dynamics (MD) simulation adds a critical biological factor of assessing the conformational transformations of ligand–receptor complexes over time under physiological conditions such as body temperature.^68,69^ Following the MM/GBSA docking, the complexes were further assessed under molecular dynamics simulation using the Desmond molecular dynamics platform in Maestro 13.6 (Schrodinger 2023-2 release). A specific system corresponding to each receptor–ligand complex was generated through the Desmond system builder. This system was solvated with the TIP3P solvent model in a buffer system. The orthorhombic box filled with a buffer solution was placed at 10.0 Å away from the complex.^70^ Additionally, the sodium and chloride ions were added to a final concentration of 0.15 M to mimic a physiological environment. Lastly, the solvent box volume was minimized under the OPLS4 force field making a final system with more than 55 000 atoms.^70,71^

The final system was retrieved upon executing the MD simulation. This system was relaxed under the default relaxation protocol and the simulation period was set to 100 ns. Using the Nose–Hover chain thermostat and Martyna–Tobias–Klein barostat, simulation was conducted under an isothermal–isobaric NPT entity at a temperature of 310 K and a pressure of 1.103 bar.^70,72^ The reversible reference system propagator algorithm (RESPA) integrator was used to accurately predict the molecular motions for near and far atoms using time step sizes of 2.0 fs and 6.0 fs, respectively. Furthermore, the Coulombic cutoff was selected to be 9.0 Å as per the default program. Following the simulation run, the results were loaded onto Maestro workspace through the Desmond simulation interaction diagram and analyzed.^72,73^

#### 2.9.10. *In silico* pharmacokinetic (ADMET) properties and drug-likeness predictions

The pkCSM web server was utilized^74^ to predict descriptors for both ADMET (absorption, distribution, metabolism, excretion, and toxicity) and drug-likeness properties of the finally selected probable inhibitors. A total of 12 molecular descriptors were generated for (ADMET) characterization of the probable EGFR TK inhibitor. Pfizer's rule (Lipinski's rule of five) was also employed to predict probable physicochemical properties of the potential inhibitor using essential drug-like properties (molecular weight, octanol–water partitioning coefficient (log *P*), hydrogen bond donors, and hydrogen bond acceptors).^75^

## 3. Results and discussion

### 3.1. Chemistry

**Scheme 1:**
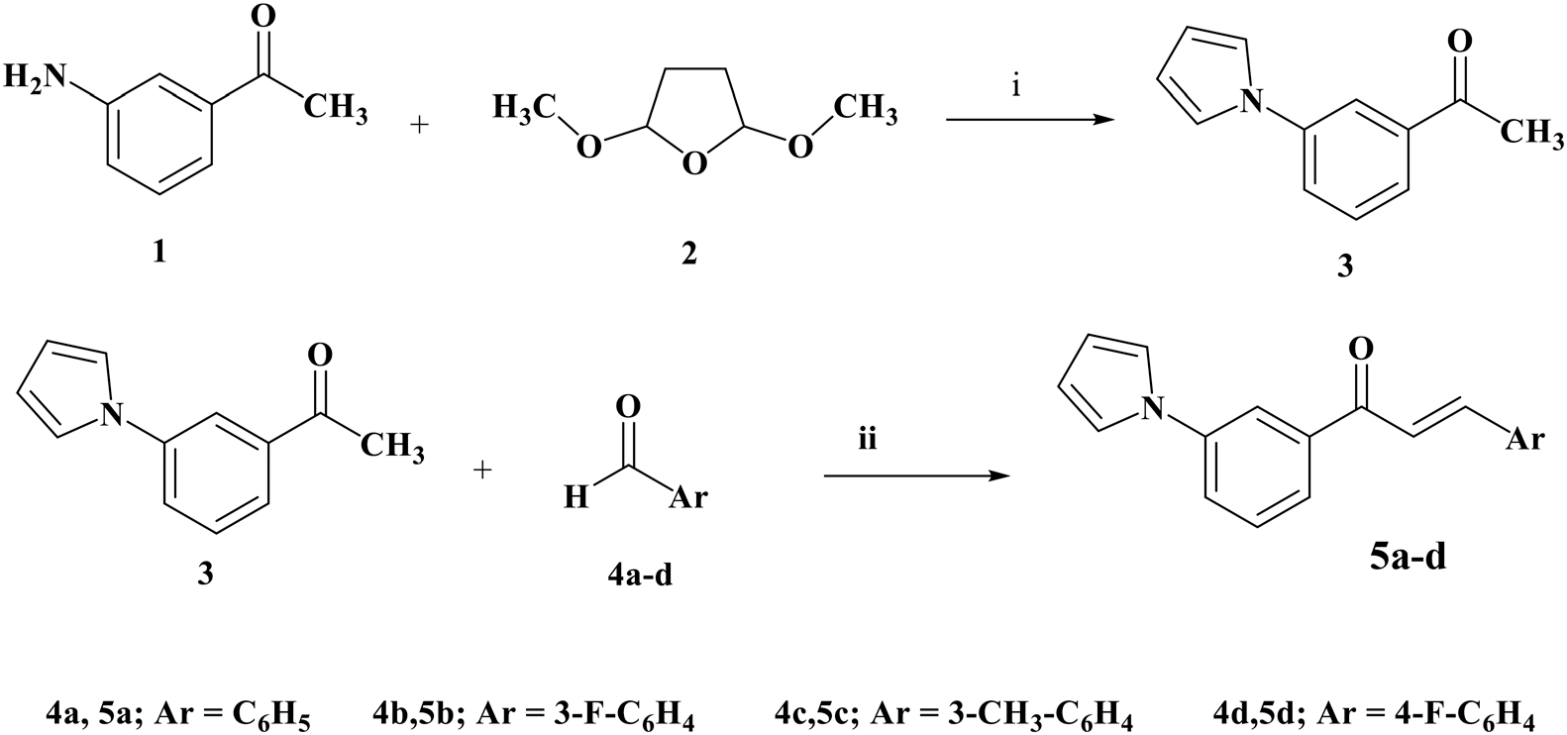
Synthesis of compounds 5a–d. Reagents and conditions: (i) glacial acetic acid, reflux 3 h. (ii) NaOH, absolute ethanol, stirring 24 h.

**Scheme 2:**
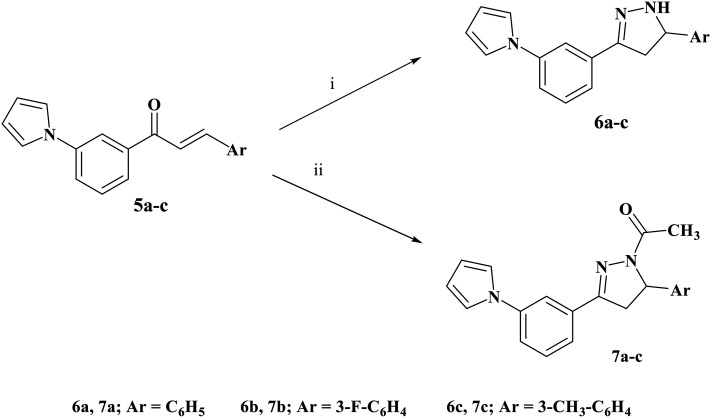
Synthesis of compounds 6a–c and 7a–c. Reagents and conditions: (i) hydrazine hydrate, absolute ethanol, reflux 16 h. (ii) Hydrazine hydrate, glacial acetic acid, reflux 20 h.

All compounds were synthesized using the protocol specified in Schemes 1 and 2. The Paal–Knorr reaction produced (3-pyrrol-1-yl)acetophenone 3 by condensing 3-amino acetophenone 1 with 2,5-dimethoxytetrahydrofuran 2. Using a sodium hydroxide catalyst, chalcones 5a–d were synthesized in ethanol by Claisen Schmidt condensation of 3 with substituted aromatic aldehydes (4a–d; Scheme 1).

The proposed structures of chalcones 5a–d were thoroughly confirmed by elemental analysis and comprehensive spectral characterization techniques. Infrared (IR) spectroscopy exhibited characteristic absorption bands for the carbonyl (C=O) group ranging from 1720 to 1725 cm^−1^, indicative of the α,β-unsaturated ketone functionality typical of chalcones. Aromatic C–H stretching vibrations appeared near 3000 cm^−1^, consistent with the aromatic rings present in the molecules. The ^1^H NMR spectra further validated the structures, showing a distinctive doublet for the olefinic proton at *δ* 6.25–6.43 ppm with a coupling constant (*J*) of approximately 15 Hz, confirming the *trans*-configuration of the double bond. The corresponding olefinic proton appeared as multiplets overlapping with aromatic proton signals, reflecting the expected spin coupling. All carbon resonances appeared at anticipated chemical shifts in the ^13^C NMR spectra, confirming the chalcone backbone and substitution pattern.

Chalcone derivatives 5a–c were reacted with hydrazine hydrate in ethanol to yield pyrazoline derivatives 6a–c (Scheme 2). Elemental and spectral analyses confirmed the formation of these products. IR spectra showed the disappearance of the carbonyl absorption bands and the emergence of broad NH stretching peaks at 3385–3390 cm^−1^, characteristic of pyrazoline ring formation. The ^1^H NMR spectra revealed the loss of olefinic doublet signals and the appearance of three characteristic doublets of doublets at *δ* 3.04–3.11 ppm, 3.23–3.28 ppm, and 4.89–4.99 ppm, corresponding to the protons on the pyrazoline ring, confirming successful cyclization.

In a parallel synthetic route, chalcones 5a–c were treated with hydrazine hydrate in the presence of glacial acetic acid to afford acetyl pyrazoline derivatives 7a–c (Scheme 2). Structural confirmation was achieved through elemental and spectral analyses. IR spectra revealed the disappearance of amino group absorption bands and the appearance of a carbonyl band at 1662–1670 cm^−1^, indicative of acetyl functionality. The ^1^H NMR spectra displayed a singlet at *δ* 2.24–2.45 ppm, attributable to the acetyl methyl protons. All carbon resonances in the ^13^C NMR spectra appeared at expected chemical shifts, consistent with the proposed acetyl pyrazoline structures.

Together, the combined elemental and spectral data provide comprehensive validation of each synthetic step—from chalcone formation to pyrazoline ring closure and acetylation. The IR, ^1^H NMR, and ^13^C NMR spectral features consistently support the structural assignments and confirm the expected chemical transformations. These detailed characterization results ensure the reliability of the synthetic methodology and the purity of the final products.

The synthetic pathway utilized simple, time-consuming, and cost-efficient methods. The starting materials are readily available and cheap materials, the reaction time is short, and the reaction conditions are regular. The products are easily collected by filtration in pure and high yield.

### 3.2. Biological activity

#### 3.2.1. Cytotoxic activity screening and SAR discussion

All three series of pyrazoline–pyrrole hybrid compounds (series 5, 6, and 7), each bearing a pyrrolo–phenyl moiety linked to chalcone, pyrazoline, or acetyl pyrazoline scaffolds, were evaluated for *in vitro* cytotoxic activity against nine human tumor cell lines at 10 μM concentration (Tables 1 and 2).

**Table 1:** *In vitro* growth promotion percentage for compounds 5a–d and 6a at 10 μM concentration against 58 cancer cell lines in the subpanel

Panel	Subpanel	5a	5b	5c	5d	6a
Leukemia	CCRF-CEM	99.55	103.36	109.74	106.60	96.14
	HL-60(TB)	101.62	84.8	86.3	80.60	98.01
	K-562	95.95	73.96	78.16	84.45	108.06
	MOLT-4	106.04	96.16	98.24	99.71	100.94
	RPMI-8226	93.26	98.23	98.38	100.89	97.32
	SR	100.51	84.3	91.71	101.36	112.65
Non-small cell lung cancer	A549/ATCC	99.88	103.58	101.05	103.38	101.42
	EKVX	100.65	102	99.36	97.17	86.87
	HOP-62	116.18	123.03	120.42	114.41	108.14
	NCI-H226	100.97	95.49	94.43	96.60	95.14
	NCI-H23	86.53	91.54	95.61	91.65	92.05
	NCI-H322M	97.56	94.21	97.88	99.28	102.59
	NCI-H460	107.98	100.61	111.92	108.03	97.69
	NCI-H522	96.97	98.49	97.49	99.04	97.86
Colon cancer	COLO 205	121.6	123.97	121.73	118.42	116.68
	HCC-2998	109.01	111.44	108.47	107.96	111.99
	HCT-116	91.57	83.81	92.77	89.12	103.48
	HCT-15	91.88	92.49	90.33	91.46	97.4
	HT29	109.21	117.46	114.39	110.73	117.43
	KM12	101.43	99.19	103.91	96.45	99.24
	SW-620	93.04	92.16	102.47	102.69	102.3
CNS cancer	SF-268	115.55	113.92	112.82	108.28	107.41
	SF-295	114.8	102.28	100.95	106.01	104.08
	SF-539	105.3	106.43	107.03	107.83	109.4
	SNB-19	94.57	93.27	97.37	97.17	99.02
	SNB-75	103.57	106.03	97.88	117.07	125.19
	U251	98.05	98	102.94	100.91	103.17
Melanoma	LOX IMVI	95.85	91.91	99.27	90.87	100.85
	MALME-3M	96.49	100.25	93.8	96.59	86.91
	M14	99.72	104.21	105.98	107.42	100.91
	MDA-MB-435	103.55	102.65	105.02	99.65	106.71
	SK-MEL-2	99.58	110.11	108.63	108.22	114.24
	SK-MEL-28	108.37	105	106.34	105.85	108.55
	SK-MEL-5	99.35	98.4	99.24	99.24	99.03
	UACC-257	114.66	111.75	107.37	109.33	100.64
	UACC-62	81.33	80.71	84.8	81.2	92.81
Ovarian cancer	IGROV1	101.47	100.15	101.83	103.23	100.68
	OVCAR-4	97.43	101.73	100.04	99.98	99.83
	OVCAR-5	106.86	105.28	107.36	109.29	108.52
	OVCAR-8	104.89	102.44	102.04	103.1	100.16
	NCI/ADR-RES	103.59	105.69	104.29	109.28	102.62
	SK-OV-3	99.71	117.26	104.31	99.41	96.9
Renal cancer	786-0	103.09	95.91	96.7	99.02	108.87
	A498	85.8	82.33	96.98	88.88	97.29
	ACHN	104.54	108.64	106.13	107.44	101.84
	CAKI-1	91.57	92.07	93.33	90.39	92.32
	RXF 393	104.79	95.74	97.24	90.04	108.78
	SN12C	101.37	101.44	108.61	104.67	109.12
	TK-10	139.02	106.25	115.09	137.51	166.31
	UO-31	**84.7**	**89.07**	**99.42**	**87.04**	**90.13**
Prostate cancer	PC-3	100.79	97.2	106.48	106.18	97.29
	DU-145	100.94	97.37	104.22	104.56	108.1
Breast cancer	MCF7	**72.19**	**73.18**	**70.69**	**73.68**	**66.8**
	MDA-MB-231/ATCC	101.42	107.66	110.69	100.79	98.02
	HS 578T	98.86	107.36	108.02	98.34	85.53
	BT-549	135.75	153.28	139.59	137.37	138.14
	T-47D	89.48	94.42	100.39	94.79	92.59
	MDA-MB-468	111.12	108.91	108.55	106.92	115.34

**Table 2:** *In vitro* growth promotion percentage for compounds 6b–c and 7a–c at 10 μM concentration against 59 cancer cell lines in the subpanel

Panel	Subpanel	6b	6c	7a	7b	7c
Leukemia	CCRF-CEM	101.59	102.71	102.64	94.39	101.01
	HL-60(TB)	108.02	115.55	96.19	107.89	93.51
	K-562	98.1	95.31	89.05	99.87	92.74
	MOLT-4	130.77	107.25	**82.81**	**90.29**	**83.73**
	RPMI-8226	**97.17**	**95.39**	90.08	93.66	99.15
	SR	NT	104.75	104.95	96.21	94.84
Non-small cell lung cancer	A549/ATCC	101.42	103.57	100.35	95.19	99.23
	EKVX	**88.07**	95.51	**88.5**	95.66	96.85
	HOP-62	106.95	127.09	108.62	109.06	113.73
	HOP-92	NT	128.05	100.77	NT	NT
	NCI-H226	90.52	99.81	92.31	94.54	89.98
	NCI-H23	90.14	**93.54**	89.89	**82.07**	**87.71**
	NCI-H322M	96.92	97.37	88.83	90.33	92.11
	NCI-H460	95.37	101.79	98.19	107.05	104.48
	NCI-H522	97.93	96.57	94.51	94.8	94.88
Colon cancer	COLO 205	111.59	117.7	98.56	103.62	110.17
	HCC-2998	108.54	110.44	112.06	105.99	107.85
	HCT-116	102.68	107.78	**95.79**	**92.49**	**93.2**
	HCT-15	99.9	101.07	98.35	92.8	97.74
	HT29	109.22	107.81	108.02	100.23	114.33
	KM12	101.61	102.17	99.46	103.04	101.25
	SW-620	**98.43**	**95.33**	96.83	95.97	93.58
CNS cancer	SF-268	109.07	112.22	102.76	112.81	107.64
	SF-295	111.41	103.52	94.8	106.74	99.31
	SF-539	104	106.8	98.35	101.44	100.42
	SNB-19	**98.66**	**91.94**	**89.69**	**88.51**	**89.71**
	SNB-75	113.78	109.48	99.16	104.44	130.8
	U251	99.84	109.34	97.89	93.41	94.98
Melanoma	LOX IMVI	94.08	108.55	91.18	95.53	95.21
	MALME-3M	**89.96**	97.08	92.4	88.26	95.69
	M14	96.45	108.88	101.87	97.61	103.59
	MDA-MB-435	107.8	106.7	102.07	102.21	107.15
	SK-MEL-2	104.61	102.93	104.26	102.79	98.43
	SK-MEL-28	110.85	105.01	105.23	103.19	105.46
	SK-MEL-5	99.5	99.03	97.02	98.73	98.62
	UACC-257	106.57	111.27	106.65	103.65	105.57
	UACC-62	**92.57**	**91.32**	**78.01**	**80.96**	**82.72**
Ovarian cancer	IGROV1	**94.42**	100.88	**90.69**	96.92	93.63
	OVCAR-4	105	106.73	93.58	100.8	107.56
	OVCAR-5	114.43	106.86	98.14	**95.78**	95.26
	OVCAR-8	99.8	103.1	95.46	98.19	**95.0**
	NCI/ADR-RES	106.04	108.51	93.57	95.89	95.12
	SK-OV-3	95.59	106.37	95.51	97.26	109.88
Renal cancer	786-0	106.41	104.05	105.86	102.06	99.85
	A498	100.85	95.54	92.64	101.26	90.98
	ACHN	100.3	106.9	93.05	101.28	93.8
	CAKI-1	88.84	97.44	81.1	86.46	88.64
	RXF 393	95.8	104.97	95.21	86.15	97.03
	SN12C	103.56	94.55	98.94	97.12	94.91
	TK-10	192.89	143.84	139.21	123.1	128.19
	UO-31	**77.59**	**89.45**	**71.06**	**75.37**	**81.23**
Prostate cancer	PC-3	95.14	96.72	83.32	84.92	85.07
	DU-145	109.19	107.36	105.52	113.63	105.64
Breast cancer	MCF7	**59.13**	**82.22**	**75.46**	**75.09**	**71.3**
	MDA-MB-231/ATCC	95.66	94.38	89.88	94.8	99.92
	HS 578T	92.58	94.29	89.37	91.01	92.8
	BT-549	144.51	128.5	131.3	125.51	129.01
	T-47D	87.95	97.86	85.39	89.02	89.59
	MDA-MB-468	110.50	118.74	79.09	90.36	93.21

In series 5, compound 5a with an unsubstituted phenyl moiety exhibited moderate growth inhibition in non-small cell lung cancer NCI-H23 (13.47%), melanoma UACC-62 (18.67%), renal cancer A498 (14.20%), renal cancer UO-31 (15.30%), and breast cancer MCF7 (27.81%). Introduction of a fluoro substituent at the 3-position in 5b enhanced antiproliferative effects across leukemia K-562 (26.04%), leukemia SR (15.70%), colon cancer HCT-116 (16.19%), melanoma UACC-62 (19.29%), renal cancer A498 (17.67%), and breast cancer MCF7 (26.82%). The 3-methylphenyl analog 5c showed activity against leukemia K-562 (21.84%), melanoma UACC-62 (15.20%), and breast cancer MCF7 (29.31%). Compound 5d, bearing a 4-fluorophenyl group, inhibited leukemia HL-60 (TB) (19.40%), leukemia K-562 (15.55%), melanoma UACC-62 (18.80%), renal cancer UO-31 (12.96%), and breast cancer MCF7 (26.32%).

In series 6, compound 6a (phenyl moiety) reduced cell growth in NSCLC EKVX (13.13%), melanoma MALME-3M (13.09%), breast cancer MCF7 (33.20%), and breast cancer HS 578T (14.47%). The 3-fluorophenyl derivative 6b exhibited enhanced inhibition in renal cancer CAKI-1 (11.16%), breast cancer MCF7 (40.87%), and breast cancer T-47D (12.05%). The 3-methylphenyl compound 6c was active against renal cancer UO-31 (10.55%) and breast cancer MCF7 (17.78%).

In series 7, compound 7a (phenyl moiety) showed growth inhibition in melanoma UACC-62 (21.99%), renal cancer CAKI-1 (18.90%), renal cancer UO-31 (28.94%), prostate cancer PC-3 (16.68%), breast cancer MCF7 (24.54%), breast cancer T-47D (14.61%), and breast cancer MDA-MB-468 (20.91%). The 3-fluorophenyl derivative 7b inhibited NSCLC NCI-H23 (17.93%), melanoma UACC-62 (19.04%), renal cancer UO-31 (24.63%), prostate cancer PC-3 (15.08%), and breast cancer MCF7 (24.91%). The 3-methylphenyl analog 7c was active against leukemia MOLT-4 (16.27%), melanoma UACC-62 (17.28%), prostate cancer PC-3 (14.93%), and breast cancer MCF7 (28.70%).

The structure–activity relationship (SAR) analysis indicates that phenyl ring substitutions significantly influence antiproliferative activity, likely *via* modulation of EGFR affinity and downstream signaling. Fluoro substituents, as in 5b, 6b, and 7b, enhance activity potentially through increased lipophilicity, metabolic stability, and electronic effects that strengthen binding interactions within the EGFR ATP-binding pocket. The electron-withdrawing nature of fluorine can optimize hydrogen bonding and van der Waals contacts with key residues, improving binding affinity and potency. Positioning of the fluoro group (*meta* or *para*) may also favor steric complementarity within the receptor site, consistent with observed higher growth inhibition, such as 40.87% inhibition of MCF7 cells by 6b.

Methyl substituents in 5c, 6c, and 7c exert distinct effects: as electron-donating groups *via* hyperconjugation, they increase electron density on the aromatic ring and promote hydrophobic interactions within EGFR's binding pockets. However, steric bulk introduced by methyl groups may influence binding orientation, accounting for varied activity profiles compared to fluoro-substituted analogs. This is reflected in significant antiproliferative effects, including 29.31% inhibition of MCF7 cells by 5c.

Overall, fluoro and methyl substituents modulate the electronic and steric environment of chalcone, pyrazoline, and acetyl pyrazoline scaffolds, impacting EGFR affinity and antiproliferative efficacy. Fluoro substituents predominantly enhance potency *via* electron-withdrawing effects and metabolic stability, while methyl groups contribute through electron donation and hydrophobic interactions, yielding complementary activity profiles across diverse cancer cell lines. A significant finding is that all tested compounds consistently exhibited their highest antiproliferative activity against MCF-7 cells, which are characterized by relatively elevated EGFR expression. Intriguingly, 7c containing a 3-methylphenyl moiety inhibited the growth of leukemia MOLT-4 (16.27%), melanoma UACC-62 (17.28%), prostate cancer PC-3 (14.93%), and breast cancer MCF7 (28.70%).

#### 3.2.2. *In vitro* EGFR assay

**Fig. 3:**
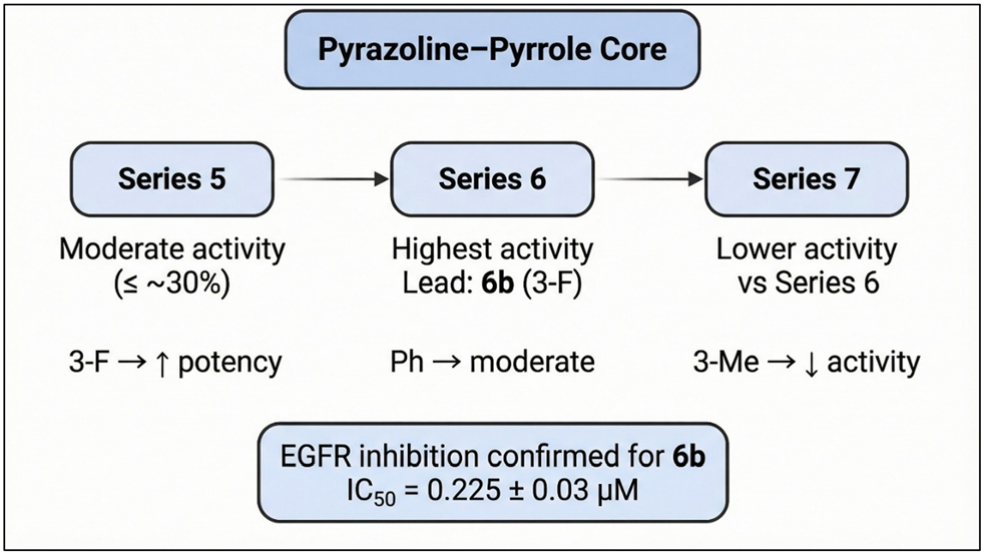
SAR summary of pyrazoline–pyrrole derivatives showing the effect of aryl substitution on antiproliferative activity and EGFR inhibition.

Compound 6b, the most active derivative in the NCI single-dose assay, was chosen to investigate its EGFR inhibitory activity. The results have been presented as a 50% concentration inhibitory value (IC_50_). Erlotinib was used in this test as a positive control (Table 3). 6b exhibited significant inhibitory activity, with an IC_50_ value of 0.225 μM compared to erlotinib's IC_50_ value of 0.198 μM (Fig. 3).

**Table 3:** IC_50_ values for compound 6b and erlotinib against EGFR. Data are presented as the mean ± SD (*n* = 3)

Compound	IC_50_ μM
6b	0.225 ± 0.03
Erlotinib	0.198 ± 0.04

#### 3.2.3. Cell cycle analysis

**Fig. 4:**
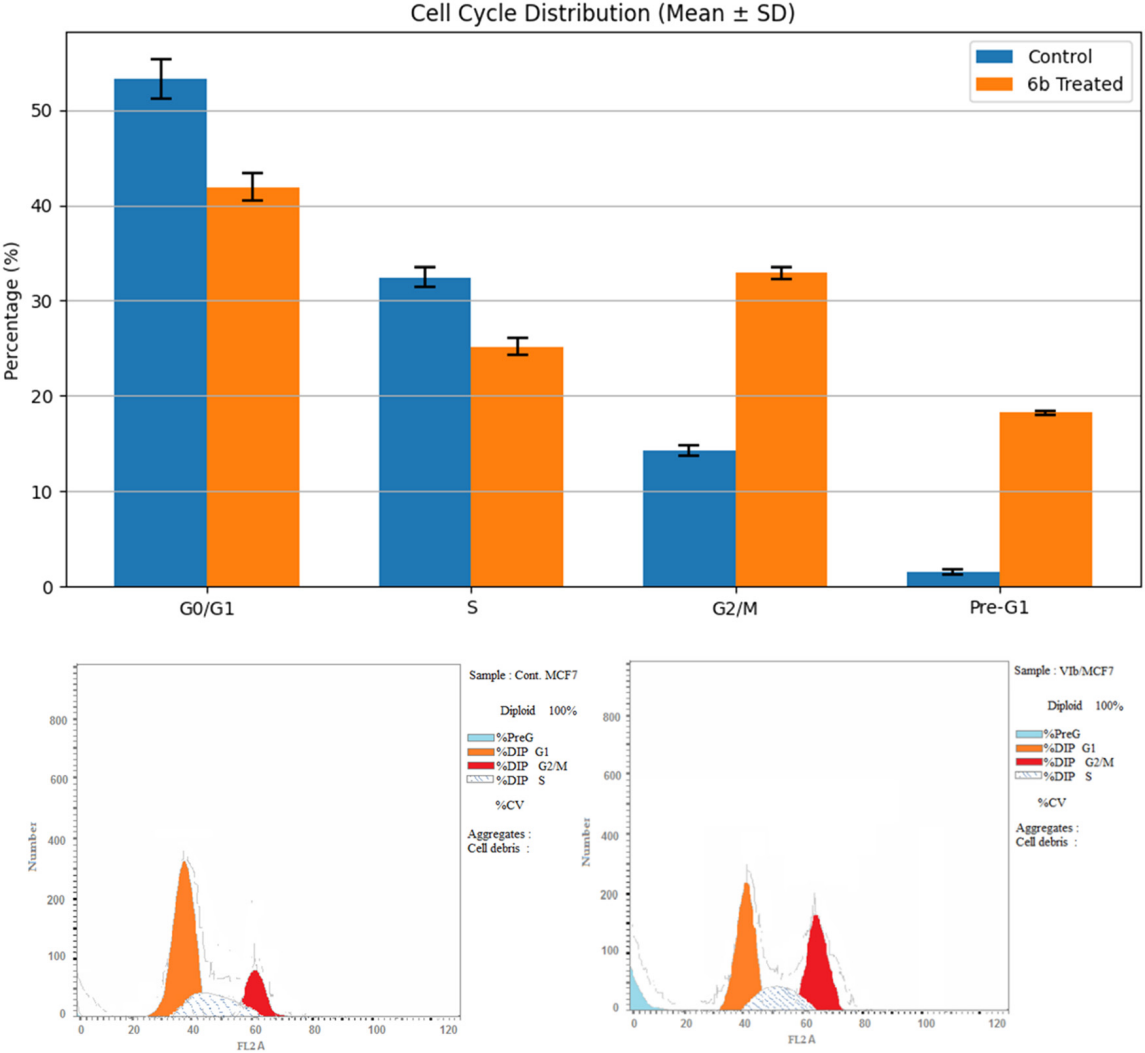
Cell cycle study and apoptotic impact of compound 6b on the MCF-7 cell line.

A flow cytometry kit was utilized to examine cells at different stages of the cell cycle, and the most potent compound 6b was tested for its ability to trigger apoptosis. MCF-7 cells were treated with 6b (10 μM), and the results are shown in Table 4 and Fig. 3. Compared with the control group, 6b markedly reduced the G0/G1 (41.92 ± 1.4% *vs.* 53.26 ± 2.1%) and S phase populations (25.16 ± 0.9% *vs.* 32.45 ± 1.0%). In contrast, a significant accumulation of cells in the G2/M phase was observed following 6b treatment (32.92 ± 0.6% *vs.* 14.29 ± 0.5%), indicating G2/M cell-cycle arrest. Moreover, 6b significantly increased the pre-G1 population (18.22 ± 0.2% *vs.* 1.57 ± 0.3%) (Fig. 4), consistent with DNA fragmentation and apoptosis induction.

**Table 4:** Effect of compound 6b on caspase-7 levels in the MCF-7 cell line

Compound	Caspase-7 conc.	FLD
6b/MCF7	396.13	7.38
Cont. MCF7	52.97	1

#### 3.2.4. Apoptosis assay

As a fundamental mechanism of tissue homeostasis, apoptosis plays an essential role in the growth and maintenance of the tissues. Deficiencies in normal apoptotic pathways contribute to the development and progression of cancer.^76,77^

**Fig. 5:**
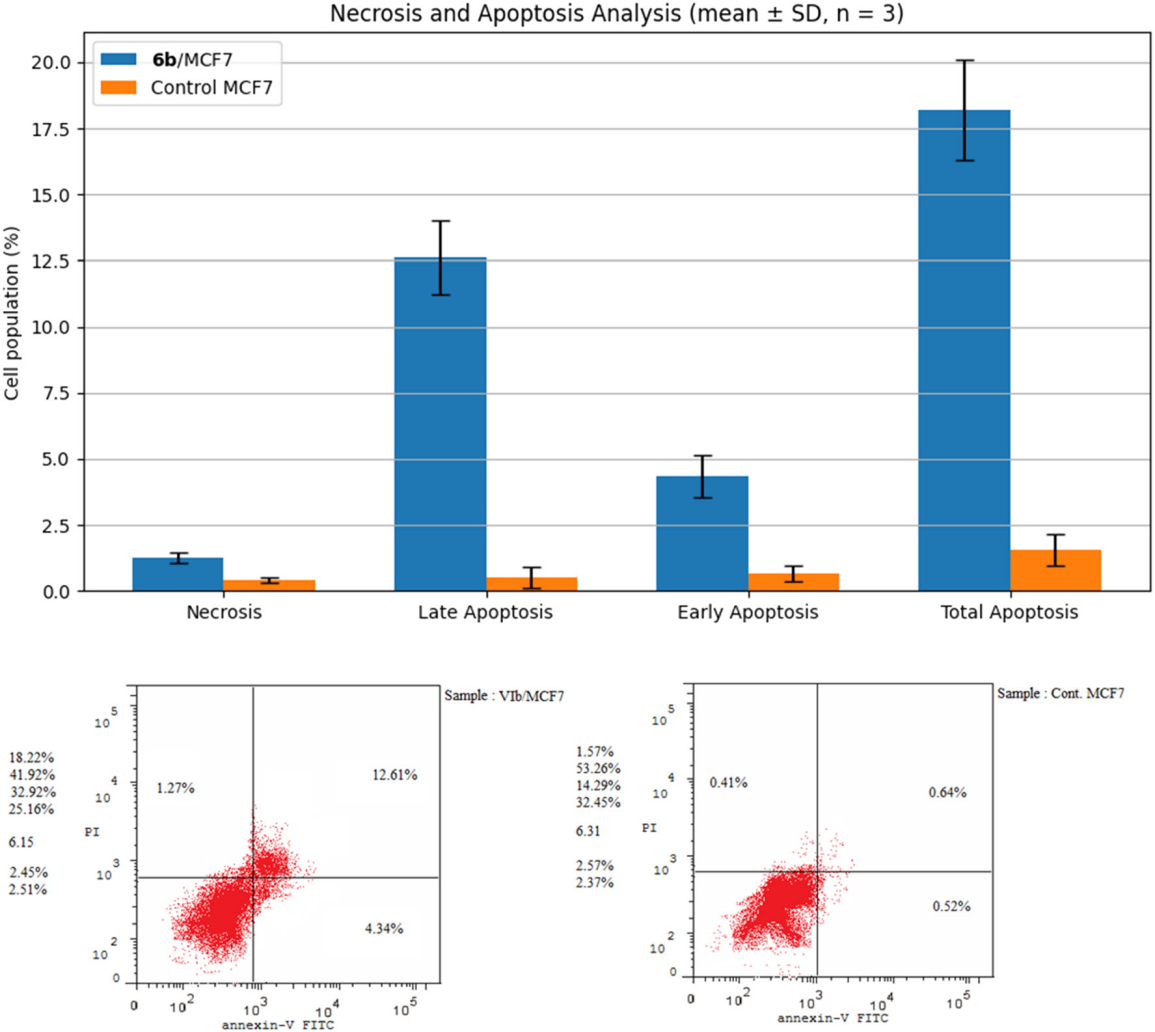
The effect of compound 6b on the % of MCF-7 cells stained with Annexin V-FITC.

Compared with the control group, compound 6b markedly increased the total apoptotic population (18.22 ± 1.9% *vs.* 1.57 ± 0.6%). This effect was primarily attributed to a pronounced elevation in late apoptosis (12.61 ± 1.4% *vs.* 0.52 ± 0.4%), while early apoptosis was also increased (4.34 ± 0.8% *vs.* 0.64 ± 0.3%). In contrast, necrotic cell death remained low and showed only a slight increase following 6b treatment (1.27 ± 0.2% *vs.* 0.41 ± 0.1%) (Fig. 5). These findings indicate that 6b predominantly induces apoptotic cell death in MCF-7 cells rather than necrosis.

#### 3.2.5. Caspase-7 analysis

**Fig. 6:**
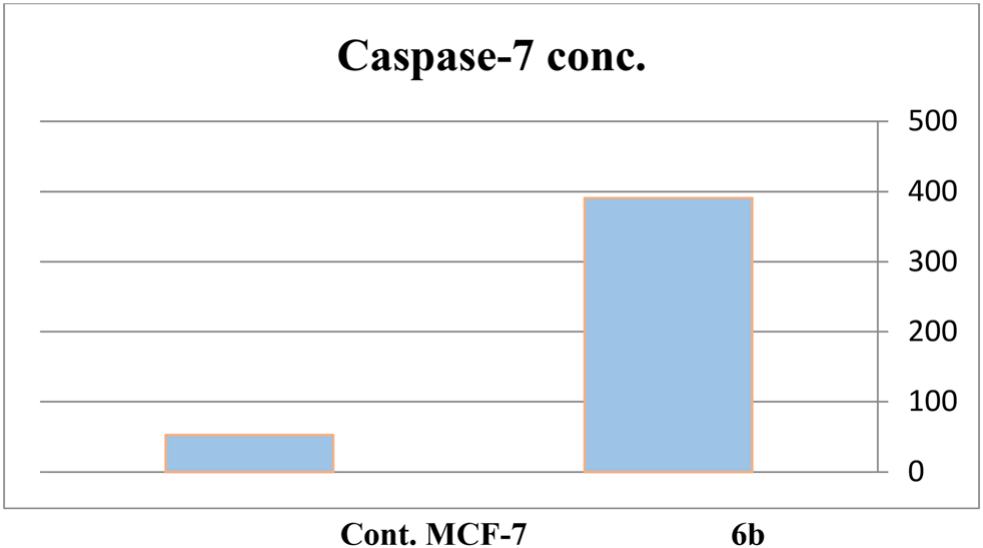
The efficacy of compound 6b on caspase-7 activity.

MCF-7, the most sensitive cell line, was evaluated for caspase-7 levels following treatment with compound 6b. Caspase-7 levels in MCF-7 cells increased 7.38 times after the treatment. Cell cycle analysis and Annexin V tests confirmed that compound 6b induced apoptosis through activation of caspase 7 (Table 4, Fig. 6).

#### 3.2.6. Bax, p53 and Bcl-2 determination

The mitochondrial apoptotic pathway is regulated by a balance between pro-apoptotic and anti-apoptotic proteins, including Bax and Bcl-2, respectively.^78,79^ As shown in Table 5, treatment of MCF-7 cells with compound 6b at 10 μM for 48 hours resulted in a significant increase in the transcription levels of Bax and p53 by 6.8- and 7.58-fold, respectively, alongside a marked decrease in Bcl-2 expression to 0.37-fold. This shift in the Bax/Bcl-2 ratio favors apoptosis induction and confirms mitochondrial involvement in cell death.

**Table 5:** Effect of compound 6b on Bax, p53 and Bcl-2 expression levels

Cod.	Bax conc.	FLD	P53 conc.	FLD	Bcl-2 conc.	FLD
6b/MCF7	408.35	6.8	425.64	7.58	2.17	0.37
Cont. MCF7	60.05	1	56.19	1	5.86	1

These apoptotic changes can be mechanistically linked to the inhibition of EGFR signaling by compound 6b, as demonstrated by docking, molecular dynamics, and cytotoxicity data. EGFR inhibition disrupts downstream pro-survival pathways such as PI3K/AKT and RAS/RAF/MEK/ERK, which normally promote cell proliferation and suppress apoptosis. The blockade of these pathways reduces the transcription and activity of anti-apoptotic proteins like Bcl-2, while enhancing pro-apoptotic factors including Bax and the tumor suppressor p53. The elevated Bax/Bcl-2 ratio increases mitochondrial outer membrane permeability, leading to cytochrome c release, caspase activation, and programmed cell death.

Therefore, the observed modulation of Bax, Bcl-2, and p53 gene expression upon 6b treatment aligns with its demonstrated EGFR inhibitory activity and provides a cohesive mechanistic link between receptor blockade and activation of the mitochondrial apoptotic pathway in MCF-7 cells (Table 5).

#### 3.2.7. DNA fragmentation assay

**Fig. 7:**
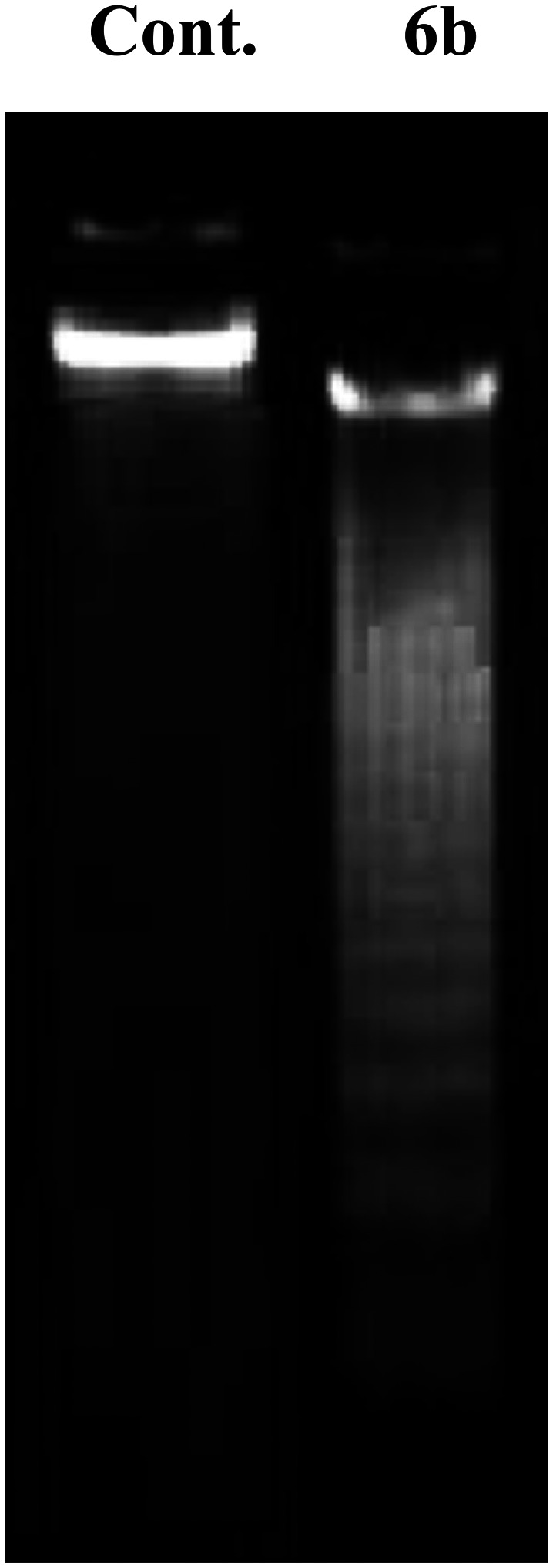
DNA fragmentation of MCF-7 cells after incubation with 6b.

Apoptosis is characterized by DNA laddering, a phenomenon in which endogenous endonucleases break down genomic DNA into fragments.^80^ A DNA ladder assay using gel electrophoresis was conducted to identify apoptotic DNA fragments mediated by 6b. In MCF-7 cells treated with 6b, DNA fragments appeared as ladders (Fig. 7). These results indicate that 6b inhibits cancer cell growth by inducing apoptosis.

### 3.3. *In silico* analysis

#### 3.3.1. Docking studies

**Fig. 8:**
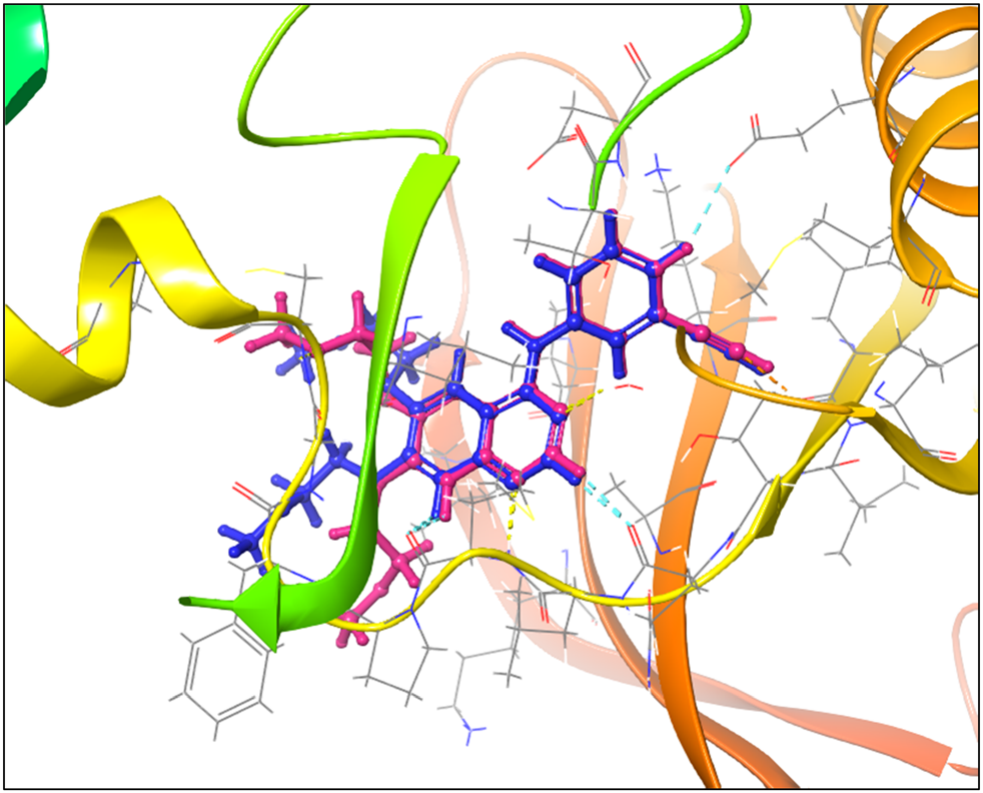
Comparative binding positions of the co-crystallized inhibitor erlotinib (blue) and the redocked inhibitor (salmon pink) within the EGFR tyrosine kinase domain (TKD) active site. The redocking validation was performed using the Glide docking protocol to assess pose prediction accuracy, yielding a root mean square deviation (RMSD) of 1.2322 Å, confirming the reliable docking methodology for subsequent compound 6b screening.

The docking binding affinities of 6b against two wild-type EGFR tyrosine kinase domains' (TKD) active and inactive states were evaluated. The EGFR tyrosine kinase inhibition profile of 6b was initially studied through docking in the binding chamber of the named target, and its binding pattern, target interactions, and binding affinities were investigated and compared to the reference erlotinib. Docking was first validated through redocking of the co-crystalline ligand erlotinib in its respective target (EGFR TKD) using the same docking procedure and protocol applied to screen 6b. This was followed by performing rigid-body superposition of the predicted lowest energy conformation for the target with its corresponding co-crystalline ligand using the structure superposition tool in Maestro. The classical root mean square deviation (RMSD) for the predicted binding poses from the co-crystalline pose was calculated. An RMSD < 2 Å cut-off value is widely regarded as the most effective threshold value for validating correctly posed molecules. The results revealed good binding mode superimposition with an RMSD of 1.2322 Å, which initially reflects the accuracy of Glide's pose prediction (Fig. 8).

**Fig. 9:**
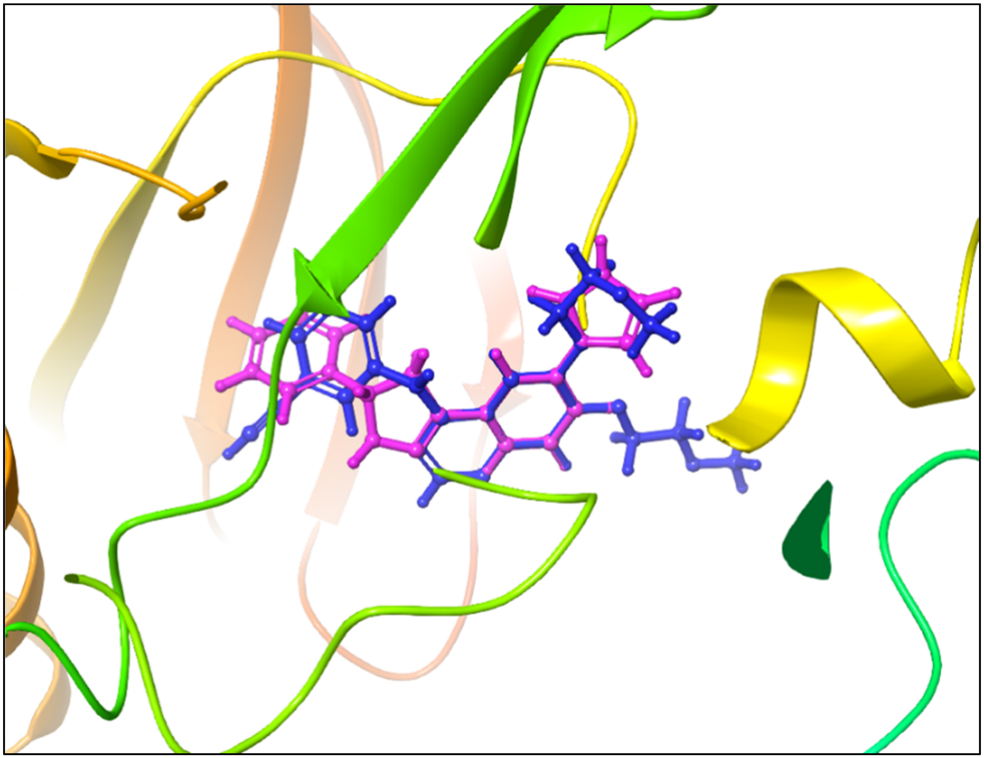
Overlay of binding poses for erlotinib (blue) and compound 6b (magenta) docked into the active site of wild-type EGFR TKD using the Glide extra precision (XP) mode. Compound 6b exhibits a highly similar binding conformation to erlotinib with an RMSD of 0.0217 Å, indicating potential affinity for the ATP-binding pocket.

6b was then docked in the wild-type EGFR TK (active state) using the extra precision mode in Glide. The results showed that 6b adapted a relatively similar docking pattern in the active site that is close to the reference drug erlotinib pose, which suggests the potential affinity of 6b to the EGFR active site (Fig. 9). The calculated RMSD for the binding pose in the reference to erlotinib was 0.0217 Å.

#### 3.3.2. Computational analysis of 6b binding to EGFR TKD (active state)

**Fig. 10:**
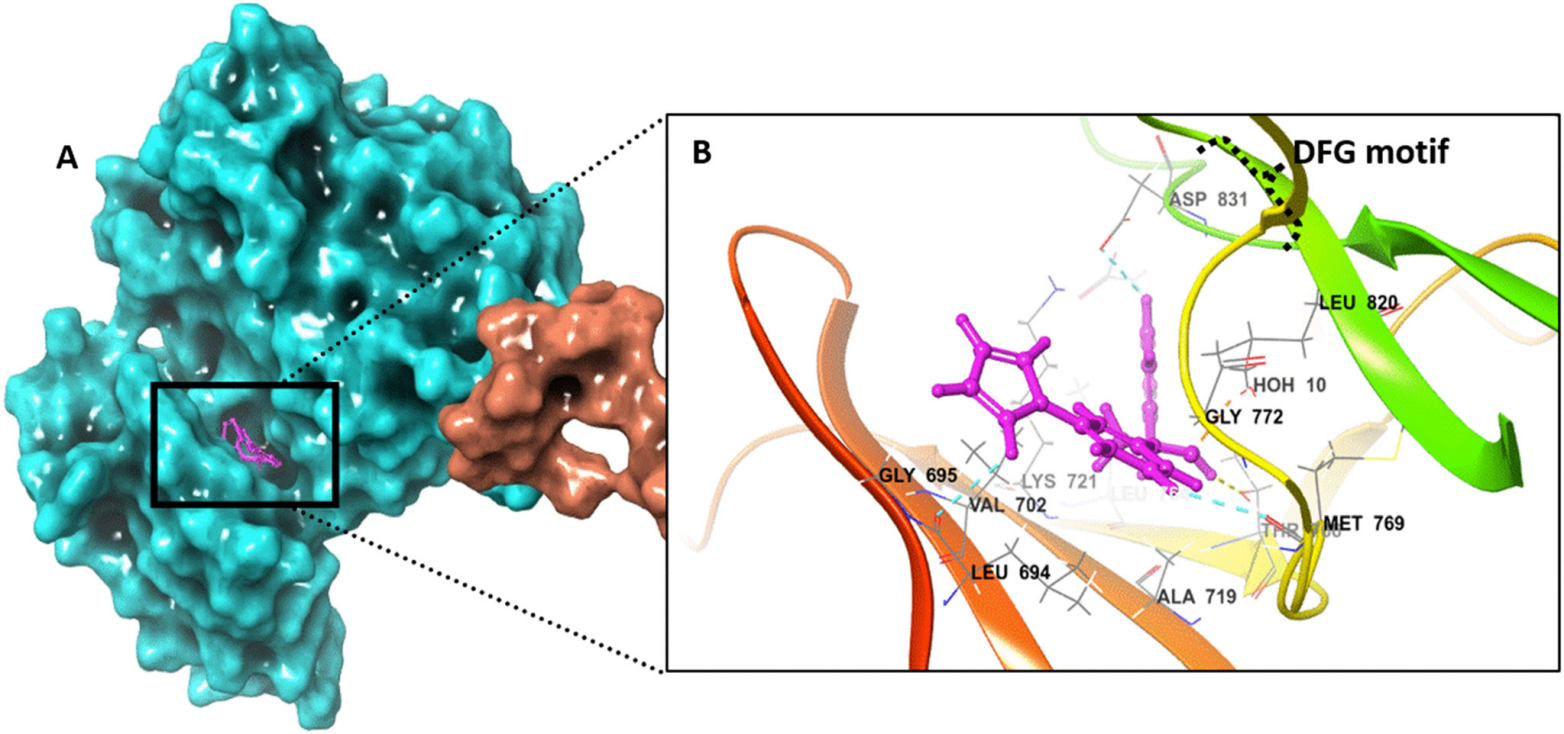
A. Three-dimensional docking pose of compound 6b within the active conformation of EGFR TKD, generated using the induced fit docking (IFD) protocol to account for receptor flexibility. B. Key interactions include ionic–π contacts with residues LYS721 and GLU738, hydrogen bonds between the pyrazoline nitrogen and residues ASP831, THR766, and MET769, and a coordinated water molecule, and hydrophobic contacts with LEU694 and VAL702. Docking was performed under standard conditions with flexible ligand and receptor side chains.

Rigid docking protocols were applied to investigate the relative binding affinities (Glide scores) of 6b to the active conformation of EGFR-TKD. Induced fit docking (IFD) was applied to generate an accurate complex structure for 6b–EGFR TK and save true binders that were poorly scored (false negatives). This can be achieved using additional receptor conformations obtained from the IFD protocol instead of screening against a single rigid conformation. 6b exhibited favorable binding-based 3D docking positions to interact with key residues within the binding pocket, as shown in Fig. 10. Interestingly, the ionic–π interactions with LYS721 and GLU738, as well as ASP831, which is the important DFG motif gatekeeper. The presence of pyrazoline nitrogen drove hydrogen bond interaction with ASP831, THR766, MET769, and one water molecule. Additionally, weak interactions with LEU694 and VAL702 were observed. The docking scores for rigid analysis were approximately 50% lower compared to the reference inhibitor erlotinib. However, flexible analysis performed better and was about 20% lower than erlotinib.

To examine the strength of the interaction between the ligand and EGFR TK protein, as well as to understand the nature of the interaction and determine the energy contribution of each ligand residue, molecular mechanics, generalized Born, and surface area solvation (MM/GBSA) free binding energy calculations were conducted.^81^ These are known for their accuracy and cost-effectiveness compared to other methods. The results showed 6b having lower binding affinity than the reference antagonist, erlotinib (Table 6).

**Table 6:** Docking scores and free binding energies (MM/GBSA) of 6b and co-crystallized reference controls at the binding site of EGFR TK (PDB entry: 1M17)

Compound	Glide score docking (rigid)^a^ kcal mol^−1^	Induced-fit docking (IFD) (flexible)^a^ kcal mol^−1^	Free binding energy (MM/GBSA)^a^ kcal mol^−1^	Ionic interaction	H-bond interaction	Hydrogen bond length (Å)	Hydrophobic interactions
6b–EGFR (active conformation)	−6.488	−8.774	−58.42	LYS721	ASP831, THR766	ASP831 = 1.87	LEU694, VAL702
				GLU738	MET769	THR766 = 1.83	
				ASP831	H_2_O (10)	MET769 = 2.96	
						H_2_O (10) = 1.86 and 2.23	
Erlotinib–EGFR (active conformation)	−9.668	−10.706	−94.21	LYS704	LYS704	LYS704 = 3.06	LEU694
				LYS721	MET769	MET769 = 1.95	VAL702
				ASP831	CYS773	CYS773 = 2.07	ALA719
					H_2_O (10)	H_2_O (10) = 1.92	LEU764
							LEU768
							MET769
							CYS773
							LEU820

a More negative values indicate higher binding interactions within the binding pocket.

The docking analysis of compound 6b within the active site of the wild-type EGFR tyrosine kinase domain (TKD) revealed a binding mode closely resembling that of erlotinib, with a low RMSD of 0.0217 Å indicating high pose accuracy. This similarity suggests that 6b can effectively occupy the ATP-binding pocket, a critical determinant of EGFR inhibition.

Key molecular interactions underpinning 6b's binding affinity include a robust hydrogen bonding network formed by the pyrazoline nitrogen atom with essential residues ASP831, THR766, and MET769, as well as coordination with a conserved water molecule. These hydrogen bonds stabilize the ligand within the binding pocket, mimicking crucial interactions observed in potent EGFR inhibitors.

In addition to hydrogen bonds, ionic–π interactions with residues LYS721 and GLU738 contribute electrostatic complementarity, facilitating proper ligand orientation and enhancing binding strength. The aromatic pyrazoline and pyrrolo–phenyl moieties engage in π–π stacking interactions with aromatic side chains lining the pocket, further stabilizing the ligand through favorable van der Waals forces and aromatic overlap.

Hydrophobic interactions with residues LEU694 and VAL702 anchor hydrophobic regions of 6b, reducing desolvation penalties and improving binding thermodynamics. These contacts are essential for maintaining the ligand's conformation and enhancing affinity.

Although rigid docking Glide scores for 6b were approximately 50% lower than those for erlotinib, induced fit docking (IFD), which accounts for receptor flexibility, improved binding scores to about 20% lower than the reference, reflecting a more accurate representation of the binding event.

Complementary MM/GBSA free energy calculations quantified the energetic contributions of these interactions, confirming the ligand's favorable binding profile despite somewhat lower affinity than erlotinib. The combined hydrogen bonding, π–π stacking, ionic–π, and hydrophobic interactions collectively rationalize 6b's observed antiproliferative activity, particularly its potent inhibition of MCF-7 cells known for elevated EGFR expression.

#### 3.3.3. Molecular dynamics simulation of 6b binding to EGFR TKD (active state)

**Fig. 11:**
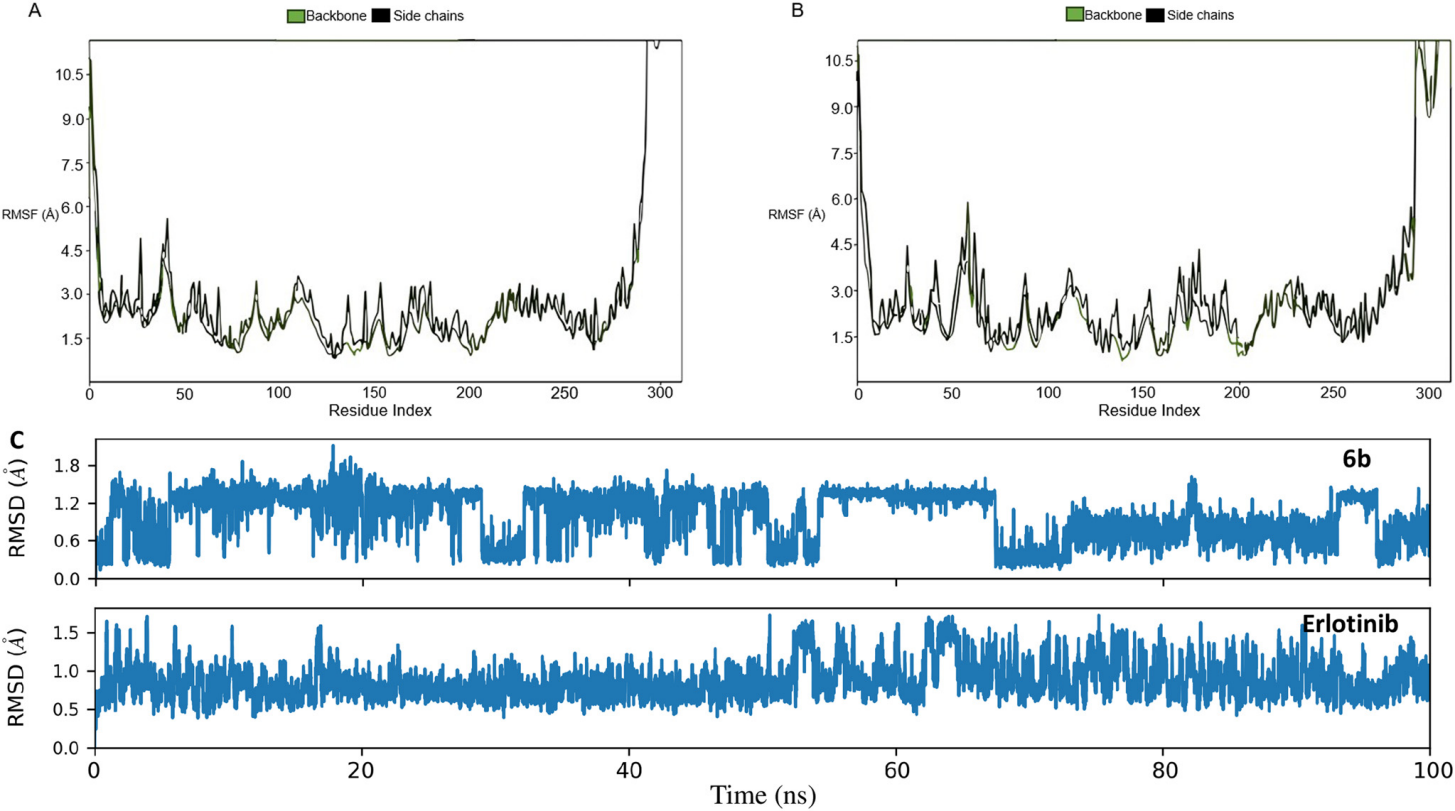
RMSF of EGFR TKD (active state) when simulated in complex with 6b (A) or erlotinib (B) as well as the individual RMSDs of 6b and erlotinib (C).

To understand the dynamic evolution of 6b binding to the active state of EGFR, the MD simulation was performed over the course of 100 ns. Using the protein backbone and its side chains, it was observed that EGFR TKD (active state) experienced minimal conformational changes with an average root mean square fluctuation (RMSF) of 3 Å (Fig. 11A). This was comparable to the RMSF of the erlotinib–EGFR TKD (active state) complex (Fig. 11B). Similarly, 6b and erlotinib had minimal changes in their bound conformations given their comparable RMSD plots (Fig. 11C). Taken together, this suggests that the 6b complex in the active state of EGFR was favorable and stable.

**Fig. 12:**
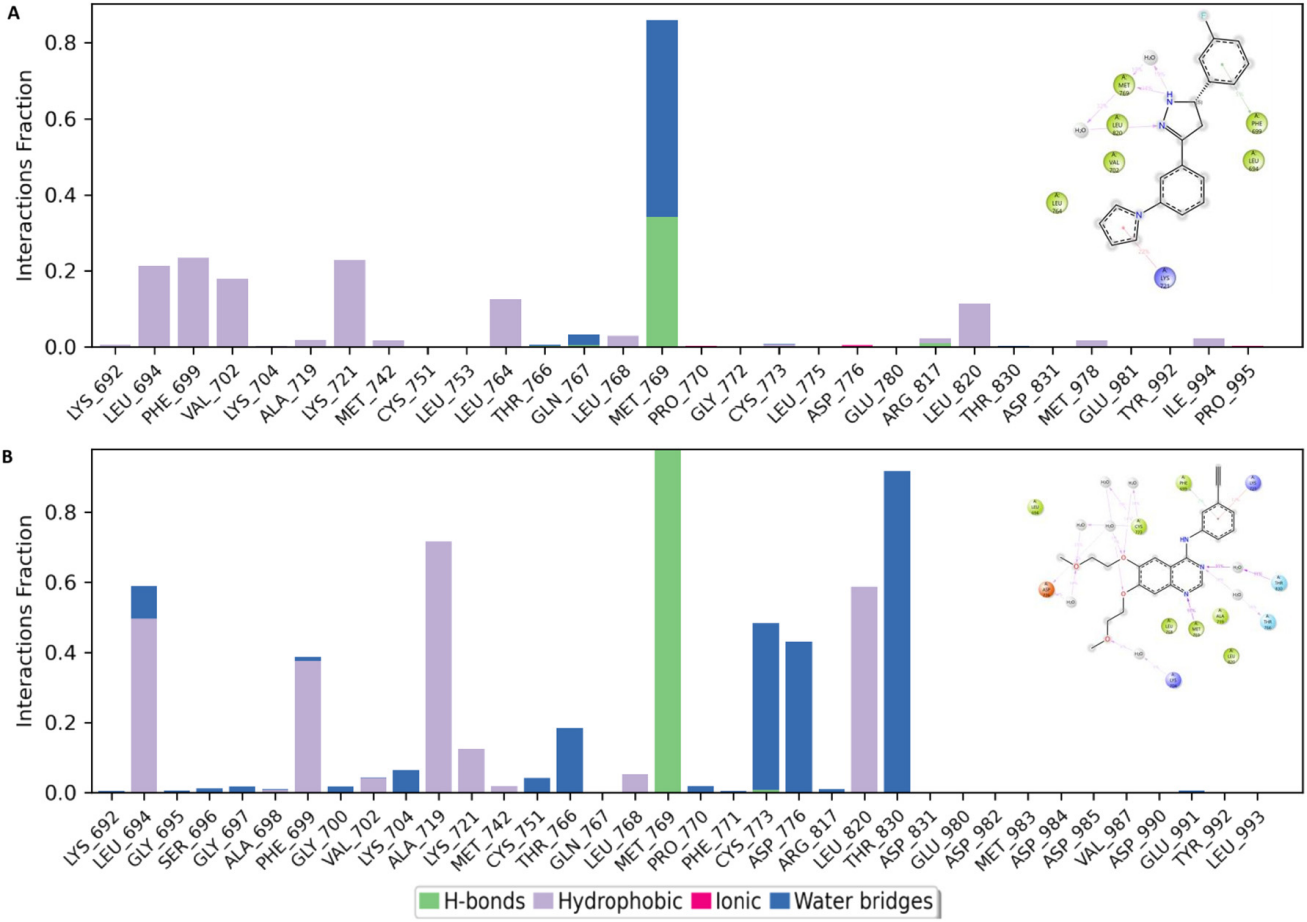
Interaction frequency analysis of individual amino acid residues within the active site of the EGFR tyrosine kinase domain (TKD) during molecular dynamics simulations with ligands 6b (A) and erlotinib (B).

Furthermore, a closer observation of the histogram of interactions revealed MET769 as the major contributor of the established binding poses in both 6b and erlotinib. This amino acid residue participated in both H-bonds and water bridges in the case of 6b while it exclusively established H-bonds in the erlotinib–EGFR TKD (active state) complex (Fig. 12A and B). 6b established various hydrophobic interactions while erlotinib had about an equal number of hydrophobic interactions and those bridged by water molecules (Fig. 12B).

#### 3.3.4. Computational analysis of 6b binding to EGFR TKD (inactive state)

**Fig. 13:**
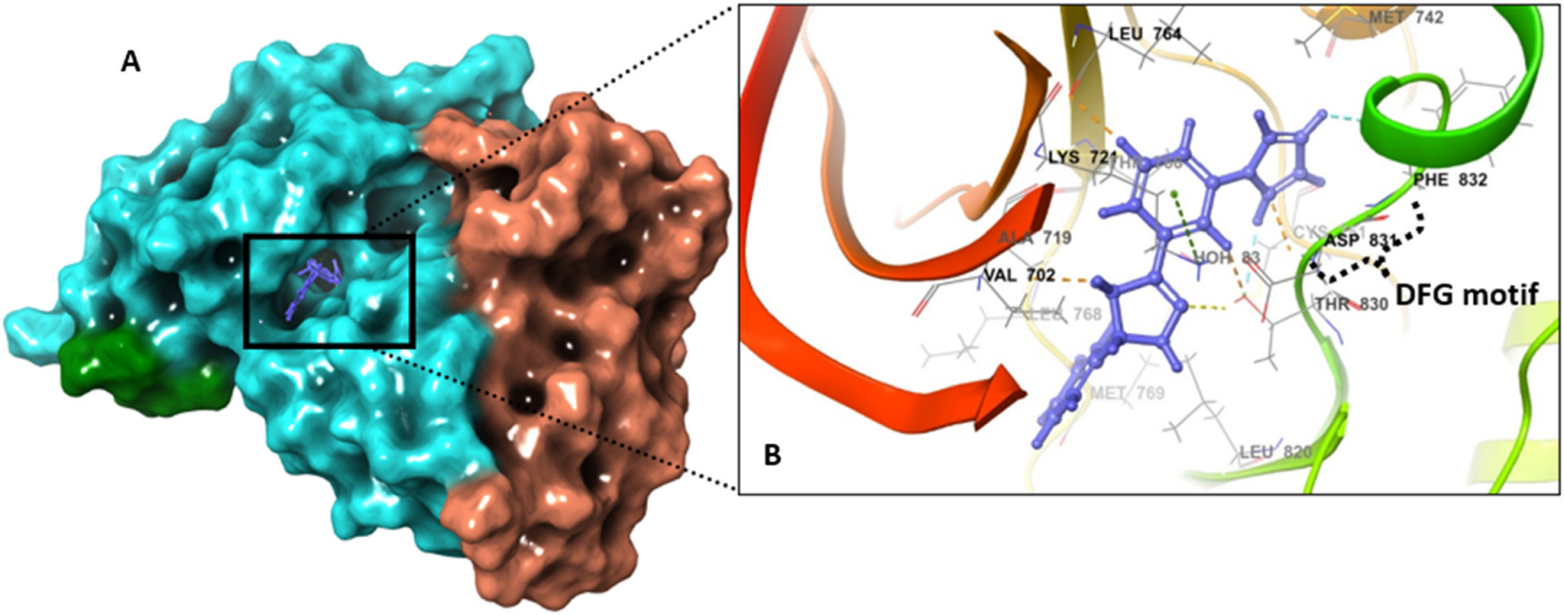
Ligand–protein binding interactions. A. Three-dimensional cartoon and surface representation of EGFR tyrosine kinase (inactive state) bound to 6b (grape) (PDB entry: 2GS7). Overall view, the X-ray crystallography structure is represented as a molecular surface. B. Focused view on the catalytic chamber showing the binding pose of 6b (ball and stick). The DFG motif was highlighted for clarity.

To evaluate the binding interaction of 6b–EGFRTKD (inactive conformation), the same computational approaches were applied to investigate whether 6b could bind to the inactive conformation of the kinase. 6b displayed a lower number of interactions compared to the active state, at least keeping the ionic–π interactions with LYS721 and ASP831 (DFG motif gatekeeper). The pyrrole formed a hydrogen bond with THR830 and the pyrazoline nitrogen with ASP831. Additionally, weak interactions formed with VAL702, LEU820, and LEU834 as displayed in Fig. 13. Interestingly, docking 6b into the inactive EGFR-TKD yielded lower docking scores of −5.987 kcal mol^−1^ and −8.223 kcal mol^−1^ for rigid and IFD-based flexible analysis, respectively. In rigid analysis, 6b had a docking score that was around 30% less than the reference inhibitor erlotinib, while in flexible analysis, it was approximately 26% lower than erlotinib. The results of the free-binding energy from MM/GMSA rescoring indicated that 6b had a lower value than the reference drug, erlotinib (Table 7).

**Table 7:** Docking scores and free binding energies (MM/GBSA) (kcal mol^−1^) of 6b and the co-crystallized reference control at the binding site of EGFR TK (PDB entry: 2GS7)

Compound	Glide score docking (rigid)^a^ kcal mol^−1^	Induced-fit docking (IFD) (flexible)^a^ kcal mol^−1^	Free binding energy (MM/GBSA)^a^ kcal mol^−1^	Ionic interaction	H-bond interaction	Hydrogen bond length (Å)	Hydrophobic interactions
6b–EGFR (inactive conformation)	−5.987	−8.223	−19.31	LYS721	THR830	THR830 = 2.80	VAL702
				ASP831	ASP831	ASP831 = 2.46	LEU820
							LEU834
Erlotinib–EGFR (inactive conformation)	−7.779	−10.409	−45.64	LYS721	MET769	MET769 = 2.15	VAL702
				ASP776	CYS773	CYS773 = 1.96	ALA719
					ASP831	ASP831 = 3.17	MET742
							LEU764
							LEU768
							MET769
							CYS773
							LEU820
							LEU834

a More negative values indicate higher binding interactions within the binding pocket.

#### 3.3.5. MD simulation of 6b binding to EGFR TKD (inactive state)

**Fig. 14:**
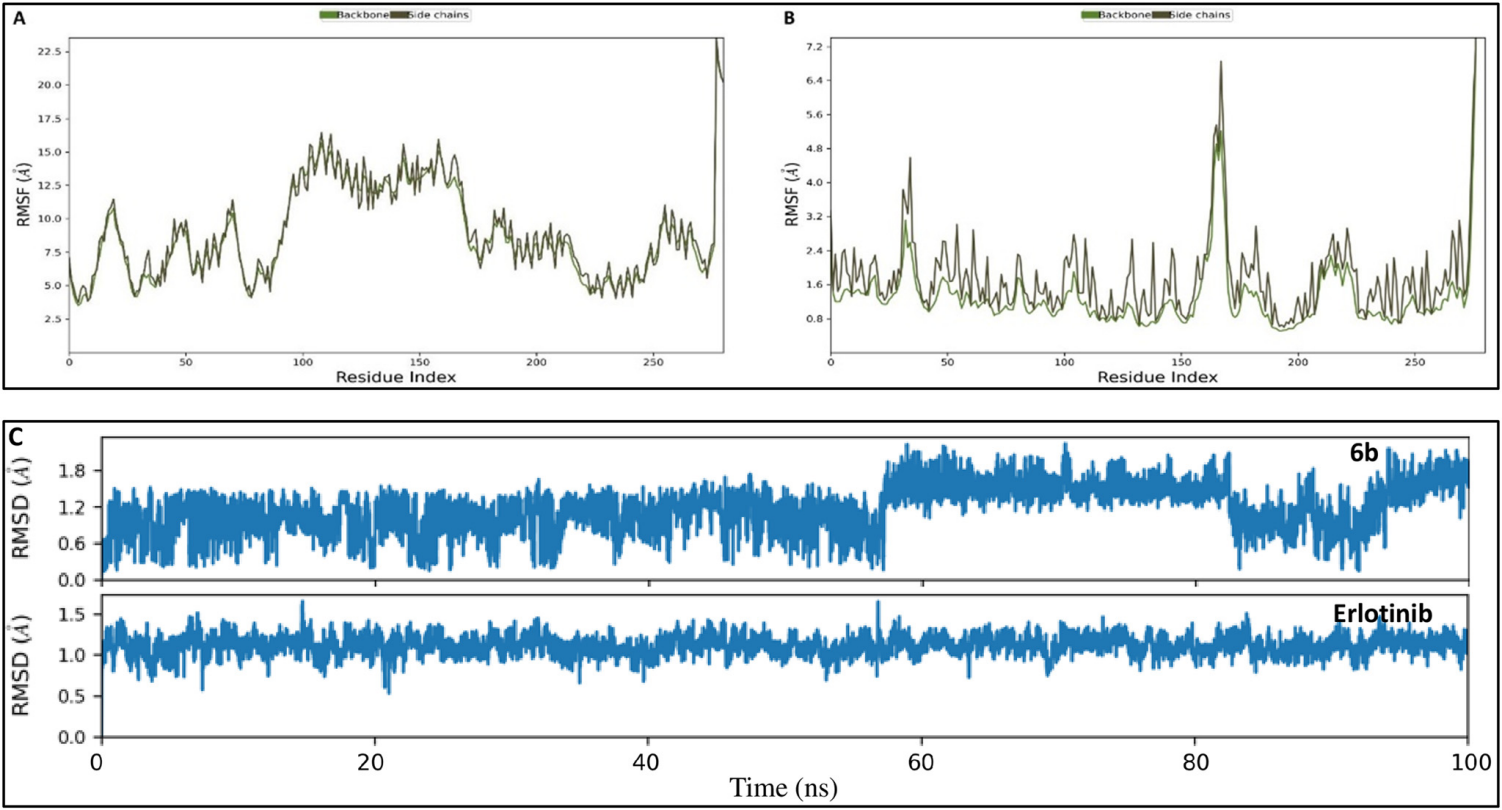
Molecular dynamics simulation analyses of EGFR TKD in the inactive conformation complexed with compound 6b and erlotinib: (A) root mean square fluctuation (RMSF) plot showing residue flexibility of EGFR TKD bound to 6b over a 100 ns simulation, indicating significant conformational changes. (B) RMSF plot for EGFR TKD bound to erlotinib under identical simulation conditions, demonstrating greater structural stability. (C) Ligand root mean square deviation (RMSD) profiles for 6b and erlotinib during the 100 ns molecular dynamics simulation, highlighting fluctuations of 6b from 57 ns onward, whereas erlotinib remains stable.

The molecular dynamics (MD) simulation results indicate that, unlike its active state, EGFR undergoes major conformational changes when bound to 6b in the inactive form (Fig. 14A). Additionally, compared to the RMSF profile obtained from the erlotinib complex (Fig. 14B), the EGFR TKD (inactive state) exhibits considerable instability in the 6b complex (Fig. 14A). Ligand RMSD analysis further supports this observation, showing fluctuations for 6b from 57 ns to the end of the simulation, whereas erlotinib remains stable throughout the simulation (Fig. 14C).

**Fig. 15:**
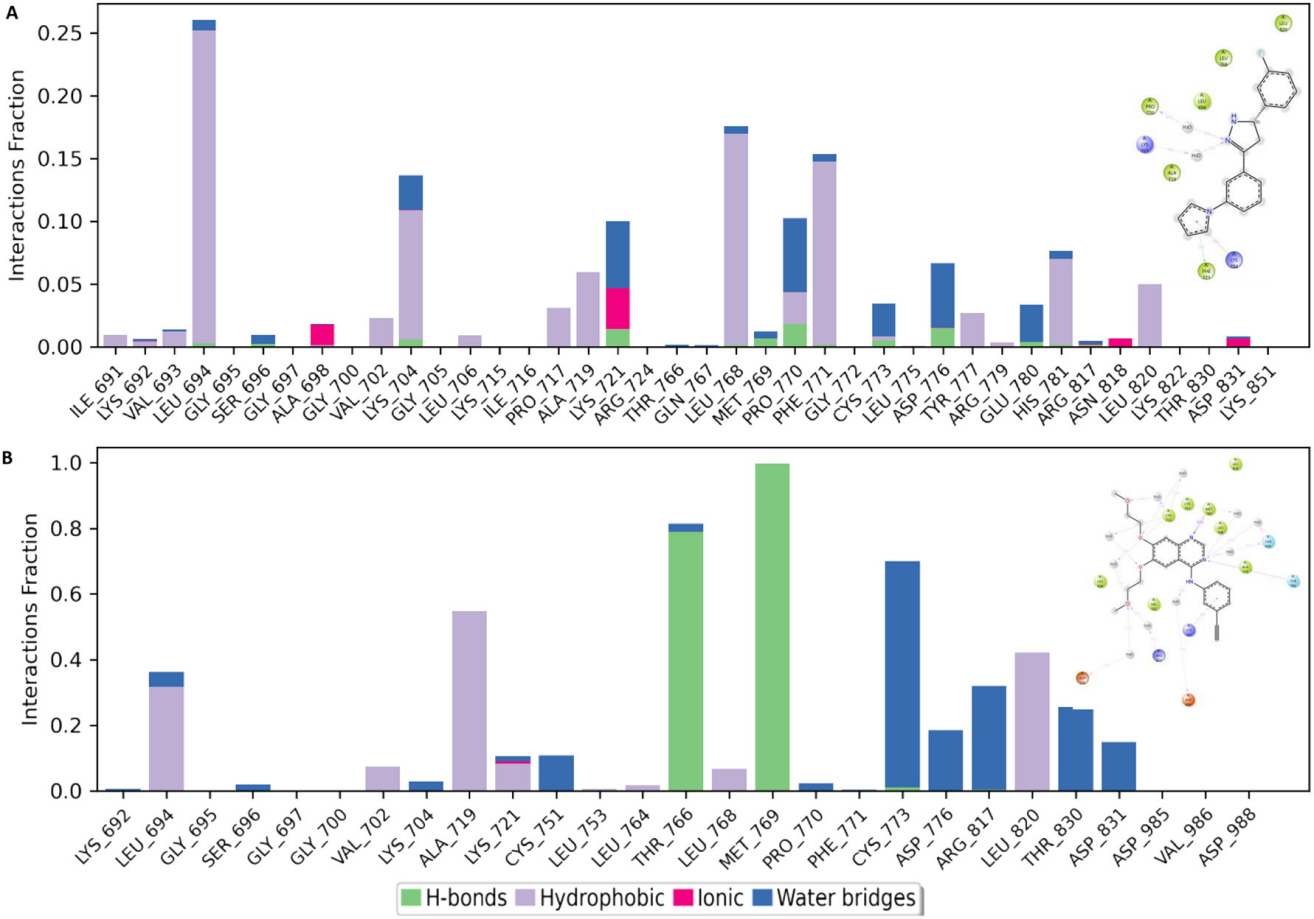
Binding interaction profiles of EGFR TKD (inactive conformation) with: (A) compound 6b, showing predominant hydrophobic interactions with residue LEU694 and minimal hydrogen bonding with MET769. (B) Erlotinib, maintaining stable hydrogen bonding interactions with MET769, critical for EGFR inhibition. Interaction frequencies were derived from 100 ns molecular dynamics trajectories analyzed using standard contact criteria.

Hydrophobic interactions with LEU694 predominate in the 6b inactive complex (Fig. 15A), while erlotinib maintains critical hydrogen bonding with MET769 (Fig. 15B), a key residue for EGFR inhibition.

These findings align with the known behavior of established EGFR inhibitors such as erlotinib, which preferentially bind to and stabilize the active conformation of EGFR TKD through persistent hydrogen bonds with residues like MET769. The relative instability and weaker interactions of 6b in the inactive state suggest reduced inhibitory potential in this conformation, consistent with the mechanism of many clinically validated EGFR inhibitors that target the active kinase state for effective inhibition.

Therefore, the MD results position 6b as a compound with binding characteristics and conformational preferences similar to known EGFR inhibitors, favoring the active TKD conformation for stable engagement. This supports the docking and binding energy data indicating 6b's potential as a competitive EGFR inhibitor with a binding profile characteristic of effective ATP-competitive inhibitors. The enhanced activity of compound 6b is attributed to the 3-fluoro substituent, which improves binding within the EGFR ATP pocket by optimizing hydrophobic contacts, maintaining the correct orientation toward the hinge residue Met793, and increasing ligand lipophilicity without steric penalty. These effects result in stronger ligand–receptor stabilization and superior biological activity relative to the phenyl and methyl analogues.

#### 3.3.6. *In silico* ADMET and drug-likeness

The assessment of ADMET properties of compounds is an essential component in the process of discovering and developing drugs. A drug candidate of high quality should exhibit satisfactory efficacy against the therapeutic target and display relevant ADMET profiling properties at therapeutic dosage.^82^ For this purpose, the 12 most relevant molecular descriptors were identified to predict the pharmacodynamic and pharmacokinetic properties of 6b in comparison with erlotinib. The molecular descriptors were employed to analyze the mode of absorption and the potential route of administration of 6b to predict bioavailability, water solubility, Caco-2 permeability, and human intestinal absorption. Table 8 presents the predicted numerical values for absorption parameters, revealing that 6b, like erlotinib, could be substantially absorbed in the intestines if administered orally. The necessary descriptors for distribution were predicted by evaluating the blood–brain barrier (BBB) and central nervous system (CNS) permeability. Based on the predicted values for both descriptors, it was determined that neither 6b nor erlotinib had the potential to permeate the CNS and BBB. Therefore, their distribution potential *via* those channels may be low. The ability of 6b to serve as both a substrate and an inhibitor to the therapeutic target was evaluated by considering CYP450 superfamily enzyme isoforms. The inhibition of CYP450 enzymes could affect drug metabolism, contraindication, and bioaccumulation, leading to an inability to facilitate the excretion of xenobiotics. 6b was not observed to inhibit CYP450 2C9 and CYP450 2D6 isoforms, as shown in Table 8. It was observed that 6b and erlotinib inhibited the CYP450 3A4 isoform, which removes around 60% of all toxic metabolites from the liver. However, both 6b and erlotinib acted positively as substrates for the enzyme isoform. Excretion is necessary to remove compounds and their metabolites from the body. Therefore, the total clearance and renal OCT2 excretory descriptors were assessed for 6b and erlotinib. The positive logarithmic values of 6b and erlotinib indicated that they could be swiftly eliminated from the system once they have completed their therapeutic effect. Conversely, 6b was recognized as a substrate for renal OCT2, a crucial component in the disposal of endogenous compounds and the renal clearance of drugs.

**Table 8:** ADMET profiling of 6b and erlotinib

ADMET parameters	Erlotinib (reference)	6b
Absorption		
Water solubility (log mol L^−1^)	−4.403	−4.977
Caco-2 permeability (log *P*_app_ in 10^−6^ cm s^−1^)	1.238	1.649
HIA (% absorbed)	95.549	96.803
Distribution		
BBB permeability (log BB)	−0.67	0.767
CNS permeability (log PS)	−3.384	−1.545
Metabolism		
CYP450 2D6 substrate (yes/no)	No	No
CYP3A4 substrate (yes/no)	Yes	Yes
CYP450 1A2 inhibitor (yes/no)	Yes	Yes
CYP450 2C19 inhibitor (yes/no)	Yes	Yes
CYP450 2C9 inhibitor	Yes	No
CYP450 2D6 inhibitor (yes/no)	No	No
CYP450 3A4 inhibitor (yes/no)	Yes	Yes
Excretion		
Total clearance (log ml min^−1^ kg^−1^)	0.591	0.385
Renal OCT2 substrate (yes/no)	No	Yes
Toxicity		
Ames toxicity (yes/no)	No	Yes
Max. tolerated dose (log mg kg^−1^ per day)	0.002	0.226
hERG1 inhibition (yes/no)	No	No
Acute oral toxicity (LD50) (mol kg^−1^)	2.368	2.17
Hepatotoxicity (yes/no)	Yes	No

Toxicological descriptors for AMES toxicity, acute oral toxicity, maximum tolerated dose, hepatotoxicity, and hERG inhibition were also computed to evaluate the pharmacodynamic properties of 6b. Interestingly, it showed favorable features to hERG inhibition and hepatotoxicity molecular descriptors, except for the AMES descriptor. Additionally, it displayed good predictive values below the acute oral toxicity test threshold. On the other hand, erlotinib was found to have a low estimated value for the toxic dose threshold of chemicals, which is consistent with other reports.^83,84^

The drug-likeness properties of 6b were assessed. Lipinski's rule of five was used in this study to determine the necessary descriptors. According to this rule, a molecule is considered a drug-like or lead contender if it has a molecular weight of less than 500 g mol^−1^. The hydrogen bond acceptor and donor number are less than 10 and 5, respectively, and the octanol–water partition coefficient (log *P*) is not greater than 5.^75^ If more than one of these properties is violated, the compound's absorption and penetration profile may not be good. 6b's drug-like profile is excellent, as shown in Table 9.

**Table 9:** Comparison of the drug-likeness properties of 6b and erlotinib

Compound	M. Wt. (K per Dalton)	Log *P*	No. H-bond donor	No. H-bond acceptor	No. of violation	Lipinski alert
6b	305.354	5.13	1	2	1	Accepted
Erlotinib (reference)	393.441	4.243	1.5	7.4	0	Accepted

## 4. Conclusion

**Fig. 16:**
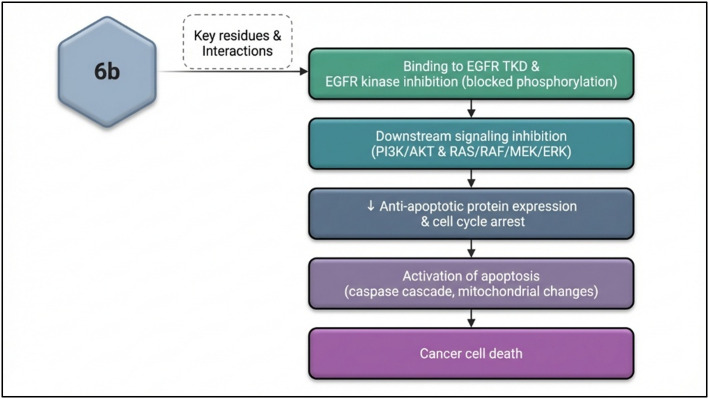
Sequential pathway of compound 6b on EGFR signaling and apoptosis induction.

This study reports the successful design and synthesis of a novel series of pyrrole–pyrazoline hybrids as potent EGFR inhibitors with promising antiproliferative activity across diverse human cancer cell lines. Compound 6b emerged as a lead candidate, demonstrating comparable EGFR enzymatic inhibition to erlotinib and significant cytotoxicity, particularly against MCF-7 breast cancer cells characterized by elevated EGFR expression (Fig. 16). Comprehensive SAR analysis revealed that fluoro substituents enhance potency through electronic and steric modulation of EGFR binding, whereas methyl groups contribute distinct hydrophobic interactions, collectively informing scaffold optimization.

Molecular docking and dynamics simulations corroborated the preferential and stable binding of 6b to the active conformation of EGFR TKD, highlighting critical hydrogen bonding, ionic–π, π–π stacking, and hydrophobic interactions that underpin its inhibitory mechanism. The observed modulation of apoptotic markers, including increased Bax/Bcl-2 ratio and p53 upregulation, establishes a mechanistic link between EGFR blockade and mitochondrial apoptosis induction.

These findings underscore the therapeutic potential of this scaffold as a basis for developing selective EGFR-targeted anticancer agents. Future work will focus on structural modifications to enhance selectivity, metabolic stability, and pharmacokinetic profiles, guided by the established SAR and computational insights, aiming to advance these hybrids toward clinical evaluation.

Not applicable. This study utilized only established cancer cell lines (NCI-58 panel), which were obtained from publicly available repositories. No human subjects or identifiable personal data were involved; therefore, Institutional Review Board (IRB) approval was not required.

## Conflicts of interest

The authors declare no conflicts of interest.

## Supplementary Material

MD-OLF-D5MD00800J-s001

## Data Availability

EGFR (active state) (PDB ID: 1M17) and EGFR (inactive state) (PDB ID: 2GS7) data for *in silico* experiments using molecular docking were obtained from the Research Collaboratory for Structural Bioinformatics (RCSB) Protein Data Bank (PDB). PDB DOI: “https://doi.org/10.2210/pdb1M17/pdb and https://doi.org/10.2210/pdb2GS7/pdb (accessed on 27 January 2024)”. The data presented in this study are available in the article and the supplementary information (SI). Supplementary information is available. See DOI: https://doi.org/10.1039/d5md00800j.
