# Supplementary material for: Design, synthesis, and anticancer evaluation of novel pyrrole–pyrazoline/chalcone hybrids: in vitro and computational insights into EGFR inhibition
Source: RSC Med Chem. 2026 Apr 27;17(5):2667–91. doi: 10.1039/d5md00800j (PMC13112205; doi:10.1039/d5md00800j)
Supplement: MD-017-D5MD00800J-s001 [file MD-017-D5MD00800J-s001.pdf]

Supplementary Information (SI) for RSC Medicinal Chemistry.
This journal is © The Royal Society of Chemistry 2026

Supplementary Materials
Design, Synthesis, and Anticancer Evaluation of Novel Pyrrole-Pyrazoline/Chalcone Hybrids: In Vitro and Computational Insights into EGFR Inhibition

Mansour S. Alturki [1]*; Marwa F. Ahmed [2]*; Abdulaziz H. Al Khzem [1]; Mohamed S. Gomaa [1]; Mohammad Sarafroz [1]; Nada Tawfeeq [1]; Mashael M. Alharbi [4]; Abdulaziz K. Al Mouslem [5]; Mohammed F. Aldawsari [6]; Wajin R. Alruwili [7]; Shah Alam Khan[8]; Radwan El-Haggar [9]; Atiah H. Almalki [2,3]*

[1] Department of Pharmaceutical Chemistry, College of Pharmacy, Imam Abdulrahman Bin Faisal University, P. O. Box 1982, Dammam 31441, Eastern Province, Kingdom of Saudi Arabia.

[2] Department of Pharmaceutical Chemistry, College of Pharmacy, Taif University, P.O. Box 11099, Taif 21944, Saudi Arabia.

[3] Addiction and Neuroscience Research Unit, Taif University, Taif 21974, Kingdom of Saudi Arabia.

[4] Department of Chemistry, College of Science, King Faisal University, Al-Ahsa 31982, Saudi Arabia.

[5] Department of Pharmaceutical Sciences, College of Clinical Pharmacy, King Faisal University, Al-Ahsa 31982, Saudi Arabia.

[6] Department of Pharmaceutics, College of Pharmacy, Prince Sattam Bin Abdulaziz University, Al-kharj-11942, Saudi Arabia.

[7] College of Pharmacy, Imam Abdulrahman Bin Faisal University, P.O. Box 1982, Dammam 31441, Saudi Arabia.

[8] Department of Pharmaceutical Chemistry, College of Pharmacy, National University of Science and Technology, PO Box 620, PC 130, Muscat, Oman

[9] Department of Pharmaceutical Chemistry, Faculty of Pharmacy, Helwan University, Cairo, 11795, Egypt.

* **Corresponding authors**:

Dr. Mansour S. Alturki, **Email:** msalturki@iau.edu.sa

Dr. Marwa F. Ahmed, **Email:** marwafarag80@yahoo.com, marwa.farg@tu.edu.sa, Dr. Atiah H. Almalki, **Email:** ahalmalki@tu.edu.sa

Key words: Pyrole; Chalcone, Pyrazoline; Antiproliferation; Epidermal growth factor receptor, Apoptosis.

Supplementary Materials

NCI data	4-23
Representative ^{1}H and ^{13}C NMR spectra for selected compounds	24-43
High-resolution mass spectrometry (HRMS) data for 6b	44

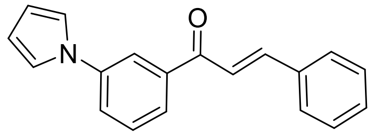
O
N

5a

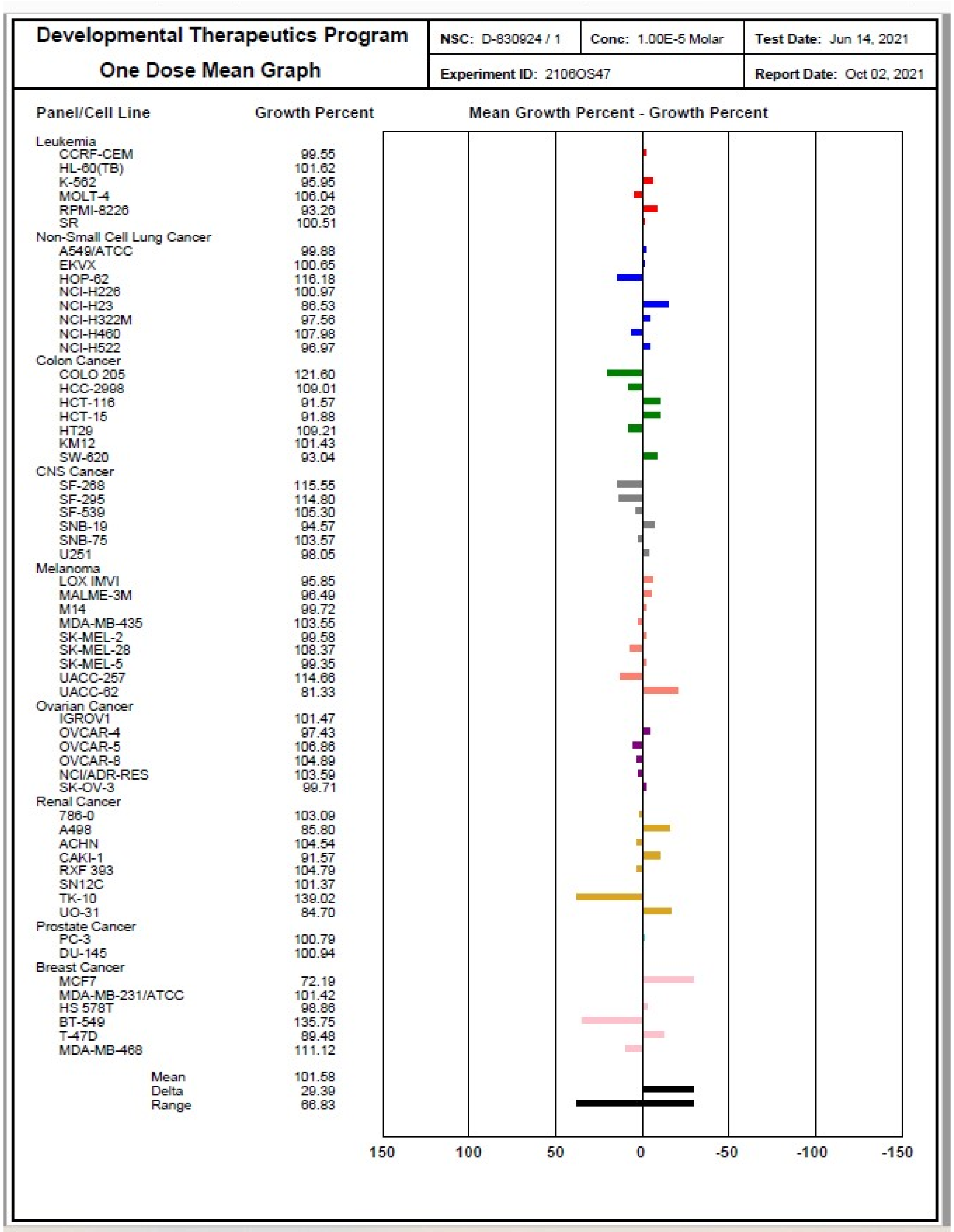
Developmental Therapeutics Program
One Dose Mean Graph
NSC: D-830924 / 1
Conc: 1.00E-5 Molar
Test Date: Jun 14, 2021
Experiment ID: 2106OS47
Report Date: Oct 02, 2021
Panel/Cell Line
Growth Percent
Mean Growth Percent - Growth Percent
Leukemia
CCRF-CEM 99.55
HL-60(TB) 101.62
K-562 95.95
MOLT-4 106.04
RPMI-8226 93.26
SR 100.51
Non-Small Cell Lung Cancer
A549/ATCC 99.88
EKVX 100.65
HOP-62 116.18
NCI-H226 100.97
NCI-H23 86.53
NCI-H322M 97.56
NCI-H460 107.98
NCI-H522 96.97
Colon Cancer
COLO 205 121.60
HCC-2998 109.01
HCT-116 91.57
HCT-15 91.88
HT29 109.21
KM12 101.43
SW-620 93.04
CNS Cancer
SF-268 115.55
SF-295 114.80
SF-539 105.30
SNB-19 94.57
SNB-75 103.57
U251 98.05
Melanoma
LOX IMVI 95.85
MALME-3M 96.49
M14 99.72
MDA-MB-435 103.55
SK-MEL-2 99.58
SK-MEL-28 108.37
SK-MEL-5 99.35
UACC-257 114.66
UACC-62 81.33
Ovarian Cancer
IGROV1 101.47
OVCAR-4 97.43
OVCAR-5 106.86
OVCAR-8 104.89
NCI/ADR-RES 103.59
SK-OV-3 99.71
Renal Cancer
786-0 103.09
A498 85.80
ACHN 104.54
CAKI-1 91.57
RXF 393 104.79
SN12C 101.37
TK-10 139.02
UO-31 84.70
Prostate Cancer
PC-3 100.79
DU-145 100.94
Breast Cancer
MCF7 72.19
MDA-MB-231/ATCC 101.42
HS 578T 98.86
BT-549 135.75
T-47D 89.48
MDA-MB-468 111.12
Mean 101.58
Delta 29.39
Range 66.83
150
100
50
0
-50
-100
-150

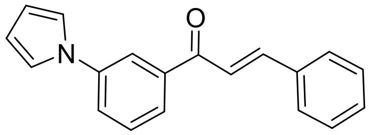

5a

A1 NSC

	A	B	C	D	E	F	G	H	I	J	K	L	M	N	O	P
1	NSC	EXPID	PLANDA	TESTSE	PREFIX	SAMPLE	DISCREI	CONC	CONCUI	CONCUI	PANELN	CELLNB	PANELN	CELLNA	GIPRCNT	
2	830924	2106OS4	Mon Jun	165	S	1	D	0.00001	M	Molar	7	3	Leukemi	CCRF-C	99.5466	
3	830924	2106OS4	Mon Jun	165	S	1	D	0.00001	M	Molar	7	8	Leukemi	HL-60(TE	101.62	
4	830924	2106OS4	Mon Jun	165	S	1	D	0.00001	M	Molar	7	5	Leukemi	K-562	95.947	
5	830924	2106OS4	Mon Jun	165	S	1	D	0.00001	M	Molar	7	6	Leukemi	MOLT-4	106.039	
6	830924	2106OS4	Mon Jun	165	S	1	D	0.00001	M	Molar	7	10	Leukemi	RPMI-82	93.2556	
7	830924	2106OS4	Mon Jun	165	S	1	D	0.00001	M	Molar	7	19	Leukemi	SR	100.515	
8	830924	2106OS4	Mon Jun	165	S	1	D	0.00001	M	Molar	1	4	Non-Sm	A549/AT	99.8814	
9	830924	2106OS4	Mon Jun	165	S	1	D	0.00001	M	Molar	1	8	Non-Sm	EKVX	100.654	
10	830924	2106OS4	Mon Jun	165	S	1	D	0.00001	M	Molar	1	26	Non-Sm	HOP-62	116.176	
11	830924	2106OS4	Mon Jun	165	S	1	D	0.00001	M	Molar	1	13	Non-Sm	NCI-H22	100.969	
12	830924	2106OS4	Mon Jun	165	S	1	D	0.00001	M	Molar	1	1	Non-Sm	NCI-H23	86.526	
13	830924	2106OS4	Mon Jun	165	S	1	D	0.00001	M	Molar	1	17	Non-Sm	NCI-H32	97.5553	
14	830924	2106OS4	Mon Jun	165	S	1	D	0.00001	M	Molar	1	21	Non-Sm	NCI-H46	107.983	
15	830924	2106OS4	Mon Jun	165	S	1	D	0.00001	M	Molar	1	3	Non-Sm	NCI-H52	96.966	
16	830924	2106OS4	Mon Jun	165	S	1	D	0.00001	M	Molar	4	10	Colon C	COLO 20	121.604	
17	830924	2106OS4	Mon Jun	165	S	1	D	0.00001	M	Molar	4	2	Colon C	HCC-29	109.007	
18	830924	2106OS4	Mon Jun	165	S	1	D	0.00001	M	Molar	4	3	Colon C	HCT-116	91.5724	
19	830924	2106OS4	Mon Jun	165	S	1	D	0.00001	M	Molar	4	15	Colon C	HCT-15	91.88	
20	830924	2106OS4	Mon Jun	165	S	1	D	0.00001	M	Molar	4	1	Colon C	HT29	109.212	
21	830924	2106OS4	Mon Jun	165	S	1	D	0.00001	M	Molar	4	17	Colon C	KM12	101.425	
22	830924	2106OS4	Mon Jun	165	S	1	D	0.00001	M	Molar	4	9	Colon C	SW-620	93.0398	
23	830924	2106OS4	Mon Jun	165	S	1	D	0.00001	M	Molar	12	14	CNS Car	SF-268	115.545	
24	830924	2106OS4	Mon Jun	165	S	1	D	0.00001	M	Molar	12	15	CNS Car	SF-295	114.796	
25	830924	2106OS4	Mon Jun	165	S	1	D	0.00001	M	Molar	12	16	CNS Car	SF-539	105.299	
26	830924	2106OS4	Mon Jun	165	S	1	D	0.00001	M	Molar	12	2	CNS Car	SNB-19	94.5725	
27	830924	2106OS4	Mon Jun	165	S	1	D	0.00001	M	Molar	12	5	CNS Car	SNB-75	103.568	
28	830924	2106OS4	Mon Jun	165	S	1	D	0.00001	M	Molar	12	9	CNS Car	U251	98.0452	
29	830924	2106OS4	Mon Jun	165	S	1	D	0.00001	M	Molar	10	1	Melanon	LOX IMV	95.8458	
30	830924	2106OS4	Mon Jun	165	S	1	D	0.00001	M	Molar	10	2	Melanon	MALME	96.4898	
31	830924	2106OS4	Mon Jun	165	S	1	D	0.00001	M	Molar	10	14	Melanon	M14	99.7188	
32	830924	2106OS4	Mon Jun	165	S	1	D	0.00001	M	Molar	5	11	Melanon	MDA-MI	103.55	
33	830924	2106OS4	Mon Jun	165	S	1	D	0.00001	M	Molar	10	5	Melanon	SK-MEL-	99.5802	
34	830924	2106OS4	Mon Jun	165	S	1	D	0.00001	M	Molar	10	8	Melanon	SK-MEL-	108.372	
35	830924	2106OS4	Mon Jun	165	S	1	D	0.00001	M	Molar	10	7	Melanon	SK-MEL-	99.3528	
36	830924	2106OS4	Mon Jun	165	S	1	D	0.00001	M	Molar	10	21	Melanon	UACC-2	114.664	
37	830924	2106OS4	Mon Jun	165	S	1	D	0.00001	M	Molar	10	20	Melanon	UACC-6	81.325	
38	830924	2106OS4	Mon Jun	165	S	1	D	0.00001	M	Molar	6	10	Ovarian (	IGROV1	101.468	
39	830924	2106OS4	Mon Jun	165	S	1	D	0.00001	M	Molar	6	2	Ovarian (	OVCAR-	97.4328	
40	830924	2106OS4	Mon Jun	165	S	1	D	0.00001	M	Molar	6	3	Ovarian (	OVCAR-	106.856	
41	830924	2106OS4	Mon Jun	165	S	1	D	0.00001	M	Molar	6	5	Ovarian (	OVCAR-	104.894	
42	830924	2106OS4	Mon Jun	165	S	1	D	0.00001	M	Molar	5	2	Ovarian (	NCI/ADF	103.59	
43	830924	2106OS4	Mon Jun	165	S	1	D	0.00001	M	Molar	6	11	Ovarian (	SK-OV-3	99.7138	
44	830924	2106OS4	Mon Jun	165	S	1	D	0.00001	M	Molar	9	18	Renal Ca	786-0	103.086	
45	830924	2106OS4	Mon Jun	165	S	1	D	0.00001	M	Molar	9	13	Renal Ca	A498	85.8043	
46	830924	2106OS4	Mon Jun	165	S	1	D	0.00001	M	Molar	9	23	Renal Ca	ACHN	104.538	
47	830924	2106OS4	Mon Jun	165	S	1	D	0.00001	M	Molar	9	15	Renal Ca	CAKI-1	91.5657	
48	830924	2106OS4	Mon Jun	165	S	1	D	0.00001	M	Molar	9	16	Renal Ca	RXF 393	104.789	
49	830924	2106OS4	Mon Jun	165	S	1	D	0.00001	M	Molar	9	8	Renal Ca	SN12C	101.368	
50	830924	2106OS4	Mon Jun	165	S	1	D	0.00001	M	Molar	9	24	Renal Ca	TK-10	139.023	
51	830924	2106OS4	Mon Jun	165	S	1	D	0.00001	M	Molar	9	4	Renal Ca	UO-31	84.7012	
52	830924	2106OS4	Mon Jun	165	S	1	D	0.00001	M	Molar	11	1	Prostate	PC-3	100.791	
53	830924	2106OS4	Mon Jun	165	S	1	D	0.00001	M	Molar	11	3	Prostate	DU-145	100.939	
54	830924	2106OS4	Mon Jun	165	S	1	D	0.00001	M	Molar	5	1	Breast C	MCF7	72.1925	
55	830924	2106OS4	Mon Jun	165	S	1	D	0.00001	M	Molar	5	5	Breast C	MDA-MI	101.425	
56	830924	2106OS4	Mon Jun	165	S	1	D	0.00001	M	Molar	5	6	Breast C	HS 578T	98.8589	
57	830924	2106OS4	Mon Jun	165	S	1	D	0.00001	M	Molar	5	13	Breast C	BT-549	135.752	
58	830924	2106OS4	Mon Jun	165	S	1	D	0.00001	M	Molar	5	14	Breast C	T-47D	89.484	
59	830924	2106OS4	Mon Jun	165	S	1	D	0.00001	M	Molar	5	18	Breast C	MDA-MI	111.122	
60																
61																
62																
63																
64																
65																
66																
67																

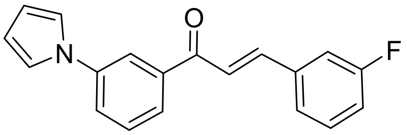

5b

Developmental Therapeutics Program

One Dose Mean Graph

**NSC:** D-830925 / 1	**Conc:** 1.00E-5 Molar	**Test Date:** Jun 14, 2021
**Experiment ID:** 2106OS47		**Report Date:** Oct 02, 2021

Panel/Cell Line	Growth Percent
Leukemia	
CCRF-CEM	103.36
HL-60(TB)	84.80
K-562	73.96
MOLT-4	96.16
RPMI-8226	98.23
SR	84.30
Non-Small Cell Lung Cancer	
A549/ATCC	103.58
EKVX	102.00
HOP-62	123.03
NCI-H226	95.49
NCI-H23	91.54
NCI-H322M	94.21
NCI-H460	100.61
NCI-H522	98.49
Colon Cancer	
COLO 205	123.97
HCC-2998	111.44
HCT-116	83.81
HCT-15	92.49
HT29	117.46
KM12	99.19
SW-620	92.16
CNS Cancer	
SF-268	113.92
SF-295	102.28
SF-539	106.43
SNB-19	93.27
SNB-75	106.03
U251	98.00
Melanoma	
LOX IMVI	91.91
MALME-3M	100.25
M14	104.21
MDA-MB-435	102.65
SK-MEL-2	110.11
SK-MEL-28	105.00
SK-MEL-5	98.40
UACC-257	111.75
UACC-62	80.71
Ovarian Cancer	
IGROV1	100.15
OVCAR-4	101.73
OVCAR-5	105.28
OVCAR-8	102.44
NCI/ADR-RES	105.69
SK-OV-3	117.26
Renal Cancer	
786-0	95.91
A498	82.33
ACHN	108.64
CAKI-1	92.07
RXF 393	95.74
SN12C	101.44
TK-10	106.25
UO-31	89.07
Prostate Cancer	
PC-3	97.20
DU-145	97.37
Breast Cancer	
MCF7	73.18
MDA-MB-231/ATCC	107.66
HS 578T	107.36
BT-549	153.28
T-47D	94.42
MDA-MB-468	108.91
Mean	100.67
Delta	27.49
Range	80.10

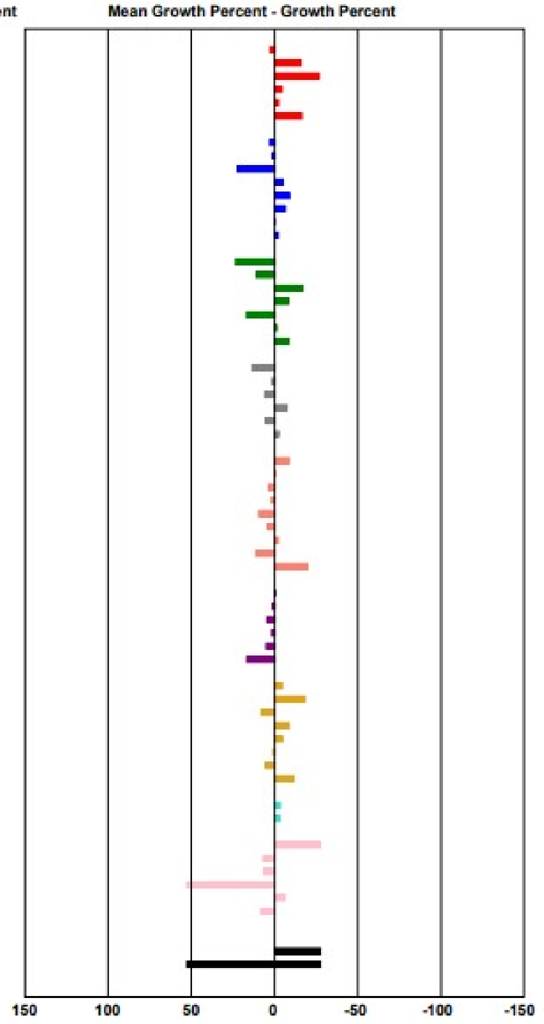

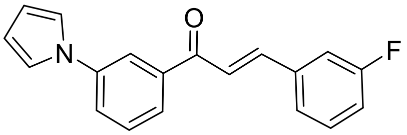

5b

A1 | NSC

1	NSC	EXPID	PLANDA	TESTSE	PREFIX	SAMPLE	DISCREI	CONC	CONCUI	CONCUI	PANELN	CELLNB	PANELN	CELLNA	GIPRCNT
2	830925	2106OS4	Mon Jun	175	S	1	D	0.00001	M	Molar	7	3	Leukemi.	CCRF-C	103.364
3	830925	2106OS4	Mon Jun	175	S	1	D	0.00001	M	Molar	7	8	Leukemi.	HL-60(TE	84.8017
4	830925	2106OS4	Mon Jun	175	S	1	D	0.00001	M	Molar	7	5	Leukemi.	K-562	73.956
5	830925	2106OS4	Mon Jun	175	S	1	D	0.00001	M	Molar	7	6	Leukemi.	MOLT-4	96.1585
6	830925	2106OS4	Mon Jun	175	S	1	D	0.00001	M	Molar	7	10	Leukemi.	RPMI-82	98.2286
7	830925	2106OS4	Mon Jun	175	S	1	D	0.00001	M	Molar	7	19	Leukemi.	SR	84.2975
8	830925	2106OS4	Mon Jun	175	S	1	D	0.00001	M	Molar	1	4	Non-Sm.	A549/AT	103.582
9	830925	2106OS4	Mon Jun	175	S	1	D	0.00001	M	Molar	1	8	Non-Sm.	EKVX	102.002
10	830925	2106OS4	Mon Jun	175	S	1	D	0.00001	M	Molar	1	26	Non-Sm.	HOP-62	123.029
11	830925	2106OS4	Mon Jun	175	S	1	D	0.00001	M	Molar	1	13	Non-Sm.	NCI-H22	95.4938
12	830925	2106OS4	Mon Jun	175	S	1	D	0.00001	M	Molar	1	1	Non-Sm.	NCI-H23	91.5374
13	830925	2106OS4	Mon Jun	175	S	1	D	0.00001	M	Molar	1	17	Non-Sm.	NCI-H32	94.213
14	830925	2106OS4	Mon Jun	175	S	1	D	0.00001	M	Molar	1	21	Non-Sm.	NCI-H46	100.606
15	830925	2106OS4	Mon Jun	175	S	1	D	0.00001	M	Molar	1	3	Non-Sm.	NCI-H52	98.4866
16	830925	2106OS4	Mon Jun	175	S	1	D	0.00001	M	Molar	4	10	Colon C.	COLO 2C	123.965
17	830925	2106OS4	Mon Jun	175	S	1	D	0.00001	M	Molar	4	2	Colon C.	HCC-29	111.437
18	830925	2106OS4	Mon Jun	175	S	1	D	0.00001	M	Molar	4	3	Colon C.	HCT-116	83.8102
19	830925	2106OS4	Mon Jun	175	S	1	D	0.00001	M	Molar	4	15	Colon C.	HCT-15	92.4915
20	830925	2106OS4	Mon Jun	175	S	1	D	0.00001	M	Molar	4	1	Colon C.	HT29	117.458
21	830925	2106OS4	Mon Jun	175	S	1	D	0.00001	M	Molar	4	17	Colon C.	KM12	99.1941
22	830925	2106OS4	Mon Jun	175	S	1	D	0.00001	M	Molar	4	9	Colon C.	SW-620	92.1633
23	830925	2106OS4	Mon Jun	175	S	1	D	0.00001	M	Molar	12	14	CNS Car	SF-268	113.924
24	830925	2106OS4	Mon Jun	175	S	1	D	0.00001	M	Molar	12	15	CNS Car	SF-295	102.284
25	830925	2106OS4	Mon Jun	175	S	1	D	0.00001	M	Molar	12	16	CNS Car	SF-539	106.434
26	830925	2106OS4	Mon Jun	175	S	1	D	0.00001	M	Molar	12	2	CNS Car	SNB-19	93.2699
27	830925	2106OS4	Mon Jun	175	S	1	D	0.00001	M	Molar	12	5	CNS Car	SNB-75	106.034
28	830925	2106OS4	Mon Jun	175	S	1	D	0.00001	M	Molar	12	9	CNS Car	U251	98.0016
29	830925	2106OS4	Mon Jun	175	S	1	D	0.00001	M	Molar	10	1	Melanon	LOX IMV	91.9082
30	830925	2106OS4	Mon Jun	175	S	1	D	0.00001	M	Molar	10	2	Melanon	MALME	100.251
31	830925	2106OS4	Mon Jun	175	S	1	D	0.00001	M	Molar	10	14	Melanon	M14	104.213
32	830925	2106OS4	Mon Jun	175	S	1	D	0.00001	M	Molar	5	11	Melanon	MDA-MI	102.654
33	830925	2106OS4	Mon Jun	175	S	1	D	0.00001	M	Molar	10	5	Melanon	SK-MEL-	110.108
34	830925	2106OS4	Mon Jun	175	S	1	D	0.00001	M	Molar	10	8	Melanon	SK-MEL-	104.997
35	830925	2106OS4	Mon Jun	175	S	1	D	0.00001	M	Molar	10	7	Melanon	SK-MEL-	98.3962
36	830925	2106OS4	Mon Jun	175	S	1	D	0.00001	M	Molar	10	21	Melanon	UACC-2!	111.747
37	830925	2106OS4	Mon Jun	175	S	1	D	0.00001	M	Molar	10	20	Melanon	UACC-6:	80.7086
38	830925	2106OS4	Mon Jun	175	S	1	D	0.00001	M	Molar	6	10	Ovarian (	IGROV1	100.152
39	830925	2106OS4	Mon Jun	175	S	1	D	0.00001	M	Molar	6	2	Ovarian (	OVCAR-	101.734
40	830925	2106OS4	Mon Jun	175	S	1	D	0.00001	M	Molar	6	3	Ovarian (	OVCAR-	105.284
41	830925	2106OS4	Mon Jun	175	S	1	D	0.00001	M	Molar	6	5	Ovarian (	OVCAR-	102.436
42	830925	2106OS4	Mon Jun	175	S	1	D	0.00001	M	Molar	5	2	Ovarian (	NCI/ADF	105.689
43	830925	2106OS4	Mon Jun	175	S	1	D	0.00001	M	Molar	6	11	Ovarian (	SK-OV-3	117.264
44	830925	2106OS4	Mon Jun	175	S	1	D	0.00001	M	Molar	9	18	Renal Ca	786-0	95.9124
45	830925	2106OS4	Mon Jun	175	S	1	D	0.00001	M	Molar	9	13	Renal Ca	A498	82.3309
46	830925	2106OS4	Mon Jun	175	S	1	D	0.00001	M	Molar	9	23	Renal Ca	ACHN	108.642
47	830925	2106OS4	Mon Jun	175	S	1	D	0.00001	M	Molar	9	15	Renal Ca	CAKI-1	92.0715
48	830925	2106OS4	Mon Jun	175	S	1	D	0.00001	M	Molar	9	16	Renal Ca	RXF 393	95.735
49	830925	2106OS4	Mon Jun	175	S	1	D	0.00001	M	Molar	9	8	Renal Ca	SN12C	101.436
50	830925	2106OS4	Mon Jun	175	S	1	D	0.00001	M	Molar	9	24	Renal Ca	TK-10	106.254
51	830925	2106OS4	Mon Jun	175	S	1	D	0.00001	M	Molar	9	4	Renal Ca	UO-31	89.0699
52	830925	2106OS4	Mon Jun	175	S	1	D	0.00001	M	Molar	11	1	Prostate	PC-3	97.1951
53	830925	2106OS4	Mon Jun	175	S	1	D	0.00001	M	Molar	11	3	Prostate	DU-145	97.37
54	830925	2106OS4	Mon Jun	175	S	1	D	0.00001	M	Molar	5	1	Breast C	MCF7	73.1769
55	830925	2106OS4	Mon Jun	175	S	1	D	0.00001	M	Molar	5	5	Breast C	MDA-MI	107.66
56	830925	2106OS4	Mon Jun	175	S	1	D	0.00001	M	Molar	5	6	Breast C	HS 578T	107.357
57	830925	2106OS4	Mon Jun	175	S	1	D	0.00001	M	Molar	5	13	Breast C	BT-549	153.283
58	830925	2106OS4	Mon Jun	175	S	1	D	0.00001	M	Molar	5	14	Breast C	T-47D	94.4161
59	830925	2106OS4	Mon Jun	175	S	1	D	0.00001	M	Molar	5	18	Breast C	MDA-MI	108.914

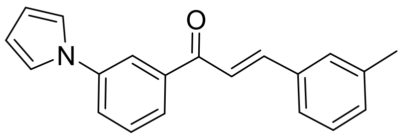

5c

Developmental Therapeutics Program<br>One Dose Mean Graph	NSC: D-830927 / 1	Conc: 1.00E-5 Molar	Test Date: Jun 14, 2021
	Experiment ID: 2106OS47		Report Date: Oct 02, 2021

Panel/Cell Line	Growth Percent
**Leukemia**	
CCRF-CEM	109.74
HL-60(TB)	86.30
K-562	78.16
MOLT-4	98.24
RPMI-8226	98.38
SR	91.71
**Non-Small Cell Lung Cancer**	
A549/ATCC	101.05
EKVX	99.36
HOP-62	120.42
NCI-H226	94.43
NCI-H23	95.61
NCI-H322M	97.88
NCI-H460	111.92
NCI-H522	97.49
**Colon Cancer**	
COLO 205	121.73
HCC-2998	108.47
HCT-116	92.77
HCT-15	90.33
HT29	114.39
KM12	103.91
SW-620	102.47
**CNS Cancer**	
SF-268	112.82
SF-295	100.95
SF-539	107.03
SNB-19	97.37
SNB-75	97.88
U251	102.94
**Melanoma**	
LOX IMVI	99.27
MALME-3M	93.80
M14	105.98
MDA-MB-435	105.02
SK-MEL-2	108.63
SK-MEL-28	106.34
SK-MEL-5	99.24
UACC-257	107.37
UACC-62	84.80
**Ovarian Cancer**	
IGROV1	101.83
OVCAR-4	100.04
OVCAR-5	107.36
OVCAR-8	102.04
NCI/ADR-RES	104.29
SK-OV-3	104.31
**Renal Cancer**	
786-0	96.70
A498	96.98
ACHN	106.13
CAKI-1	93.33
RXF 393	97.24
SN12C	108.61
TK-10	115.09
UO-31	99.42
**Prostate Cancer**	
PC-3	106.48
DU-145	104.22
**Breast Cancer**	
MCF7	70.69
MDA-MB-231/ATCC	110.69
HS 578T	108.02
BT-549	139.59
T-47D	100.39
MDA-MB-468	108.55
Mean	102.18
Delta	31.49
Range	68.90

Mean Growth Percent - Growth Percent

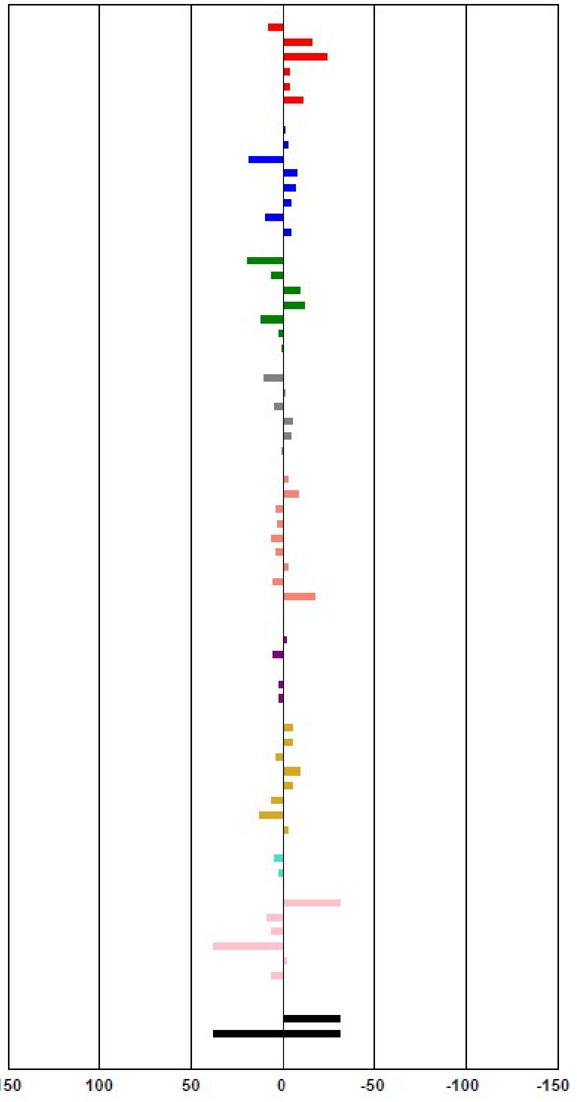

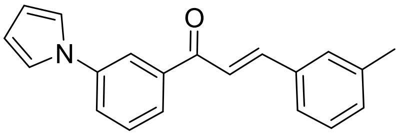

5c

A1 NSC

	NSC	EXPID	PLANDA	TESTSE	PREFIX	SAMPLE	DISCREI	CONC	CONCUI	CONCUI	PANELN	CELLNB	PANELN	CELLNA	GIPRCNT
2	830927	2106OS4	Mon Jun	195	S	1	D	0.00001	M	Molar	7	3	Leukemi	CCRF-C	109.737
3	830927	2106OS4	Mon Jun	195	S	1	D	0.00001	M	Molar	7	8	Leukemi	HL-60(TE	86.2972
4	830927	2106OS4	Mon Jun	195	S	1	D	0.00001	M	Molar	7	5	Leukemi	K-562	78.1594
5	830927	2106OS4	Mon Jun	195	S	1	D	0.00001	M	Molar	7	6	Leukemi	MOLT-4	98.2424
6	830927	2106OS4	Mon Jun	195	S	1	D	0.00001	M	Molar	7	10	Leukemi	RPMI-82	98.3812
7	830927	2106OS4	Mon Jun	195	S	1	D	0.00001	M	Molar	7	19	Leukemi	SR	91.7138
8	830927	2106OS4	Mon Jun	195	S	1	D	0.00001	M	Molar	1	4	Non-Sm	A549/AT	101.046
9	830927	2106OS4	Mon Jun	195	S	1	D	0.00001	M	Molar	1	8	Non-Sm	EKVX	99.3636
10	830927	2106OS4	Mon Jun	195	S	1	D	0.00001	M	Molar	1	26	Non-Sm	HOP-62	120.417
11	830927	2106OS4	Mon Jun	195	S	1	D	0.00001	M	Molar	1	13	Non-Sm	NCI-H22	94.4284
12	830927	2106OS4	Mon Jun	195	S	1	D	0.00001	M	Molar	1	1	Non-Sm	NCI-H23	95.613
13	830927	2106OS4	Mon Jun	195	S	1	D	0.00001	M	Molar	1	17	Non-Sm	NCI-H32	97.8806
14	830927	2106OS4	Mon Jun	195	S	1	D	0.00001	M	Molar	1	21	Non-Sm	NCI-H46	111.916
15	830927	2106OS4	Mon Jun	195	S	1	D	0.00001	M	Molar	1	3	Non-Sm	NCI-H52	97.4915
16	830927	2106OS4	Mon Jun	195	S	1	D	0.00001	M	Molar	4	10	Colon C	COLO 20	121.726
17	830927	2106OS4	Mon Jun	195	S	1	D	0.00001	M	Molar	4	2	Colon C	HCC-299	108.468
18	830927	2106OS4	Mon Jun	195	S	1	D	0.00001	M	Molar	4	3	Colon C	HCT-116	92.7678
19	830927	2106OS4	Mon Jun	195	S	1	D	0.00001	M	Molar	4	15	Colon C	HCT-15	90.3309
20	830927	2106OS4	Mon Jun	195	S	1	D	0.00001	M	Molar	4	1	Colon C	HT29	114.386
21	830927	2106OS4	Mon Jun	195	S	1	D	0.00001	M	Molar	4	17	Colon C	KM12	103.912
22	830927	2106OS4	Mon Jun	195	S	1	D	0.00001	M	Molar	4	9	Colon C	SW-620	102.467
23	830927	2106OS4	Mon Jun	195	S	1	D	0.00001	M	Molar	12	14	CNS Car	SF-268	112.824
24	830927	2106OS4	Mon Jun	195	S	1	D	0.00001	M	Molar	12	15	CNS Car	SF-295	100.949
25	830927	2106OS4	Mon Jun	195	S	1	D	0.00001	M	Molar	12	16	CNS Car	SF-539	107.027
26	830927	2106OS4	Mon Jun	195	S	1	D	0.00001	M	Molar	12	2	CNS Car	SNB-19	97.3711
27	830927	2106OS4	Mon Jun	195	S	1	D	0.00001	M	Molar	12	5	CNS Car	SNB-75	97.882
28	830927	2106OS4	Mon Jun	195	S	1	D	0.00001	M	Molar	12	9	CNS Car	U251	102.941
29	830927	2106OS4	Mon Jun	195	S	1	D	0.00001	M	Molar	10	1	Melanon	LOX IMV	99.268
30	830927	2106OS4	Mon Jun	195	S	1	D	0.00001	M	Molar	10	2	Melanon	MALME	93.7989
31	830927	2106OS4	Mon Jun	195	S	1	D	0.00001	M	Molar	10	14	Melanon	M14	105.975
32	830927	2106OS4	Mon Jun	195	S	1	D	0.00001	M	Molar	5	11	Melanon	MDA-MI	105.016
33	830927	2106OS4	Mon Jun	195	S	1	D	0.00001	M	Molar	10	5	Melanon	SK-MEL-	108.635
34	830927	2106OS4	Mon Jun	195	S	1	D	0.00001	M	Molar	10	8	Melanon	SK-MEL-	106.339
35	830927	2106OS4	Mon Jun	195	S	1	D	0.00001	M	Molar	10	7	Melanon	SK-MEL-	99.2373
36	830927	2106OS4	Mon Jun	195	S	1	D	0.00001	M	Molar	10	21	Melanon	UACC-2!	107.373
37	830927	2106OS4	Mon Jun	195	S	1	D	0.00001	M	Molar	10	20	Melanon	UACC-6:	84.8
38	830927	2106OS4	Mon Jun	195	S	1	D	0.00001	M	Molar	6	10	Ovarian (	IGROV1	101.835
39	830927	2106OS4	Mon Jun	195	S	1	D	0.00001	M	Molar	6	2	Ovarian (	OVCAR-	100.037
40	830927	2106OS4	Mon Jun	195	S	1	D	0.00001	M	Molar	6	3	Ovarian (	OVCAR-	107.356
41	830927	2106OS4	Mon Jun	195	S	1	D	0.00001	M	Molar	6	5	Ovarian (	OVCAR-	102.04
42	830927	2106OS4	Mon Jun	195	S	1	D	0.00001	M	Molar	5	2	Ovarian (	NCI/ADF	104.293
43	830927	2106OS4	Mon Jun	195	S	1	D	0.00001	M	Molar	6	11	Ovarian (	SK-OV-3	104.312
44	830927	2106OS4	Mon Jun	195	S	1	D	0.00001	M	Molar	9	18	Renal Ca	786-0	96.6957
45	830927	2106OS4	Mon Jun	195	S	1	D	0.00001	M	Molar	9	13	Renal Ca	A498	96.9801
46	830927	2106OS4	Mon Jun	195	S	1	D	0.00001	M	Molar	9	23	Renal Ca	ACHN	106.129
47	830927	2106OS4	Mon Jun	195	S	1	D	0.00001	M	Molar	9	15	Renal Ca	CAKI-1	93.3264
48	830927	2106OS4	Mon Jun	195	S	1	D	0.00001	M	Molar	9	16	Renal Ca	RXF 393	97.2359
49	830927	2106OS4	Mon Jun	195	S	1	D	0.00001	M	Molar	9	8	Renal Ca	SN12C	108.612
50	830927	2106OS4	Mon Jun	195	S	1	D	0.00001	M	Molar	9	24	Renal Ca	TK-10	115.086
51	830927	2106OS4	Mon Jun	195	S	1	D	0.00001	M	Molar	9	4	Renal Ca	UO-31	99.4246
52	830927	2106OS4	Mon Jun	195	S	1	D	0.00001	M	Molar	11	1	Prostate	PC-3	106.484
53	830927	2106OS4	Mon Jun	195	S	1	D	0.00001	M	Molar	11	3	Prostate	DU-145	104.223
54	830927	2106OS4	Mon Jun	195	S	1	D	0.00001	M	Molar	5	1	Breast C	MCF7	70.6857
55	830927	2106OS4	Mon Jun	195	S	1	D	0.00001	M	Molar	5	5	Breast C	MDA-MI	110.693
56	830927	2106OS4	Mon Jun	195	S	1	D	0.00001	M	Molar	5	6	Breast C	HS 578T	108.022
57	830927	2106OS4	Mon Jun	195	S	1	D	0.00001	M	Molar	5	13	Breast C	BT-549	139.593
58	830927	2106OS4	Mon Jun	195	S	1	D	0.00001	M	Molar	5	14	Breast C	T-47D	100.386
59	830927	2106OS4	Mon Jun	195	S	1	D	0.00001	M	Molar	5	18	Breast C	MDA-MI	108.55

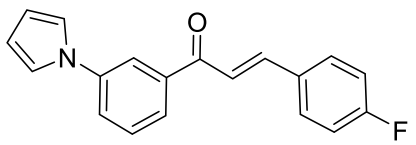

5d

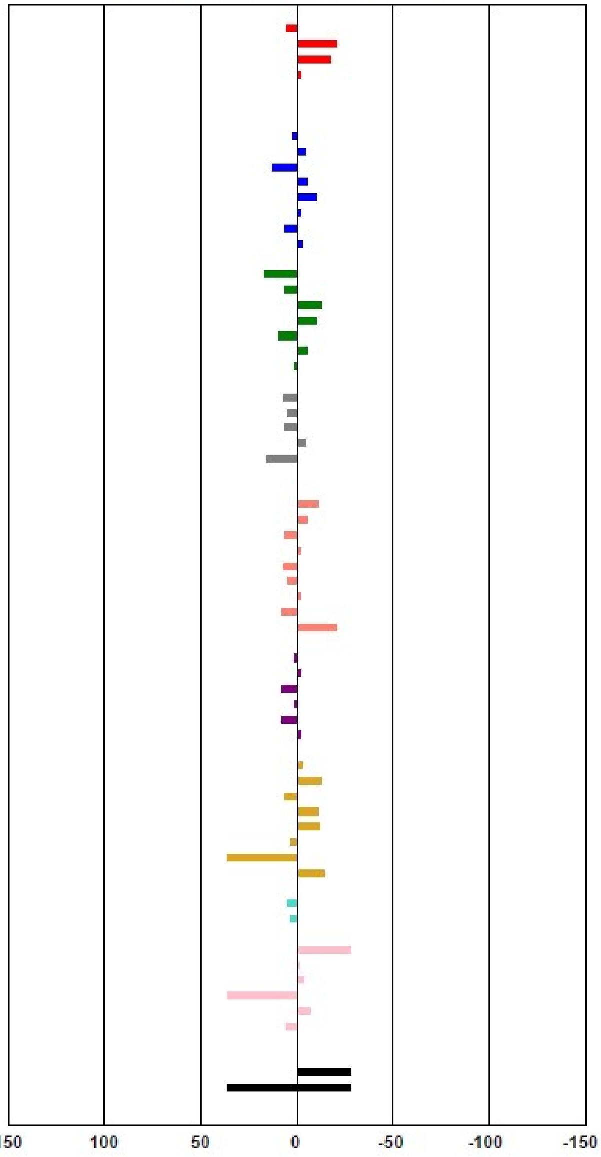

Developmental Therapeutics Program — One Dose Mean Graph

NSC: D-830926 / 1	Conc: 1.00E-5 Molar	Test Date: Jun 14, 2021
Experiment ID: 2106OS47		Report Date: Oct 02, 2021

Panel/Cell Line	Growth Percent
**Leukemia**	
CCRF-CEM	106.60
HL-60(TB)	80.60
K-562	84.45
MOLT-4	99.71
RPMI-8226	100.89
SR	101.36
**Non-Small Cell Lung Cancer**	
A549/ATCC	103.38
EKVX	97.17
HOP-62	114.41
NCI-H226	96.60
NCI-H23	91.65
NCI-H322M	99.28
NCI-H460	108.03
NCI-H522	99.04
**Colon Cancer**	
COLO 205	118.42
HCC-2998	107.96
HCT-116	89.12
HCT-15	91.46
HT29	110.73
KM12	96.45
SW-620	102.69
**CNS Cancer**	
SF-268	108.28
SF-295	106.01
SF-539	107.83
SNB-19	97.17
SNB-75	117.07
U251	100.91
**Melanoma**	
LOX IMVI	90.87
MALME-3M	96.59
M14	107.42
MDA-MB-435	99.65
SK-MEL-2	108.22
SK-MEL-28	105.85
SK-MEL-5	99.24
UACC-257	109.33
UACC-62	81.20
**Ovarian Cancer**	
IGROV1	103.23
OVCAR-4	99.98
OVCAR-5	109.29
OVCAR-8	103.10
NCI/ADR-RES	109.28
SK-OV-3	99.41
**Renal Cancer**	
786-0	99.02
A498	88.88
ACHN	107.44
CAKI-1	90.39
RXF 393	90.04
SN12C	104.67
TK-10	137.51
UO-31	87.04
**Prostate Cancer**	
PC-3	106.18
DU-145	104.56
**Breast Cancer**	
MCF7	73.68
MDA-MB-231/ATCC	100.79
HS 578T	98.34
BT-549	137.37
T-47D	94.79
MDA-MB-468	106.92
Mean	101.51
Delta	27.83
Range	63.83

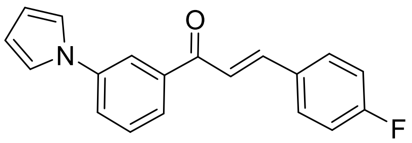

5d

A1 | *fx* | NSC

	NSC	EXPID	PLANDA	TESTSE	PREFIX	SAMPLE	DISCREI	CONC	CONCUI	CONCUI	PANELN	CELLNB	PANELN	CELLNA	GIPRCNT
2	830926	2106OS4	Mon Jun	185	S	1	D	0.00001	M	Molar	7	3	Leukemi	CCRF-C	106.604
3	830926	2106OS4	Mon Jun	185	S	1	D	0.00001	M	Molar	7	8	Leukemi	HL-60(TE	80.5999
4	830926	2106OS4	Mon Jun	185	S	1	D	0.00001	M	Molar	7	5	Leukemi	K-562	84.4545
5	830926	2106OS4	Mon Jun	185	S	1	D	0.00001	M	Molar	7	6	Leukemi	MOLT-4	99.7055
6	830926	2106OS4	Mon Jun	185	S	1	D	0.00001	M	Molar	7	10	Leukemi	RPMI-82	100.891
7	830926	2106OS4	Mon Jun	185	S	1	D	0.00001	M	Molar	7	19	Leukemi	SR	101.361
8	830926	2106OS4	Mon Jun	185	S	1	D	0.00001	M	Molar	1	4	Non-Sm	A549/AT	103.38
9	830926	2106OS4	Mon Jun	185	S	1	D	0.00001	M	Molar	1	8	Non-Sm	EKVX	97.17
10	830926	2106OS4	Mon Jun	185	S	1	D	0.00001	M	Molar	1	26	Non-Sm	HOP-62	114.414
11	830926	2106OS4	Mon Jun	185	S	1	D	0.00001	M	Molar	1	13	Non-Sm	NCI-H22	96.5951
12	830926	2106OS4	Mon Jun	185	S	1	D	0.00001	M	Molar	1	1	Non-Sm	NCI-H23	91.651
13	830926	2106OS4	Mon Jun	185	S	1	D	0.00001	M	Molar	1	17	Non-Sm	NCI-H32	99.2823
14	830926	2106OS4	Mon Jun	185	S	1	D	0.00001	M	Molar	1	21	Non-Sm	NCI-H46	108.025
15	830926	2106OS4	Mon Jun	185	S	1	D	0.00001	M	Molar	1	3	Non-Sm	NCI-H52	99.0418
16	830926	2106OS4	Mon Jun	185	S	1	D	0.00001	M	Molar	4	10	Colon C	COLO 20	118.42
17	830926	2106OS4	Mon Jun	185	S	1	D	0.00001	M	Molar	4	2	Colon C	HCC-29	107.964
18	830926	2106OS4	Mon Jun	185	S	1	D	0.00001	M	Molar	4	3	Colon C	HCT-116	89.1174
19	830926	2106OS4	Mon Jun	185	S	1	D	0.00001	M	Molar	4	15	Colon C	HCT-15	91.4582
20	830926	2106OS4	Mon Jun	185	S	1	D	0.00001	M	Molar	4	1	Colon C	HT29	110.726
21	830926	2106OS4	Mon Jun	185	S	1	D	0.00001	M	Molar	4	17	Colon C	KM12	96.4519
22	830926	2106OS4	Mon Jun	185	S	1	D	0.00001	M	Molar	4	9	Colon C	SW-620	102.687
23	830926	2106OS4	Mon Jun	185	S	1	D	0.00001	M	Molar	12	14	CNS Car	SF-268	108.285
24	830926	2106OS4	Mon Jun	185	S	1	D	0.00001	M	Molar	12	15	CNS Car	SF-295	106.005
25	830926	2106OS4	Mon Jun	185	S	1	D	0.00001	M	Molar	12	16	CNS Car	SF-539	107.835
26	830926	2106OS4	Mon Jun	185	S	1	D	0.00001	M	Molar	12	2	CNS Car	SNB-19	97.1702
27	830926	2106OS4	Mon Jun	185	S	1	D	0.00001	M	Molar	12	5	CNS Car	SNB-75	117.07
28	830926	2106OS4	Mon Jun	185	S	1	D	0.00001	M	Molar	12	9	CNS Car	U251	100.909
29	830926	2106OS4	Mon Jun	185	S	1	D	0.00001	M	Molar	10	1	Melanon	LOX IMV	90.8697
30	830926	2106OS4	Mon Jun	185	S	1	D	0.00001	M	Molar	10	2	Melanon	MALME	96.5924
31	830926	2106OS4	Mon Jun	185	S	1	D	0.00001	M	Molar	10	14	Melanon	M14	107.417
32	830926	2106OS4	Mon Jun	185	S	1	D	0.00001	M	Molar	5	11	Melanon	MDA-MI	99.6546
33	830926	2106OS4	Mon Jun	185	S	1	D	0.00001	M	Molar	10	5	Melanon	SK-MEL-	108.216
34	830926	2106OS4	Mon Jun	185	S	1	D	0.00001	M	Molar	10	8	Melanon	SK-MEL-	105.845
35	830926	2106OS4	Mon Jun	185	S	1	D	0.00001	M	Molar	10	7	Melanon	SK-MEL-	99.2398
36	830926	2106OS4	Mon Jun	185	S	1	D	0.00001	M	Molar	10	21	Melanon	UACC-2	109.334
37	830926	2106OS4	Mon Jun	185	S	1	D	0.00001	M	Molar	10	20	Melanon	UACC-6	81.1988
38	830926	2106OS4	Mon Jun	185	S	1	D	0.00001	M	Molar	6	10	Ovarian (	IGROV1	103.225
39	830926	2106OS4	Mon Jun	185	S	1	D	0.00001	M	Molar	6	2	Ovarian (	OVCAR-	99.9803
40	830926	2106OS4	Mon Jun	185	S	1	D	0.00001	M	Molar	6	3	Ovarian (	OVCAR-	109.286
41	830926	2106OS4	Mon Jun	185	S	1	D	0.00001	M	Molar	6	5	Ovarian (	OVCAR-	103.102
42	830926	2106OS4	Mon Jun	185	S	1	D	0.00001	M	Molar	5	2	Ovarian (	NCI/ADF	109.276
43	830926	2106OS4	Mon Jun	185	S	1	D	0.00001	M	Molar	6	11	Ovarian (	SK-OV-3	99.4055
44	830926	2106OS4	Mon Jun	185	S	1	D	0.00001	M	Molar	9	18	Renal Ca	786-0	99.0238
45	830926	2106OS4	Mon Jun	185	S	1	D	0.00001	M	Molar	9	13	Renal Ca	A498	88.8781
46	830926	2106OS4	Mon Jun	185	S	1	D	0.00001	M	Molar	9	23	Renal Ca	ACHN	107.435
47	830926	2106OS4	Mon Jun	185	S	1	D	0.00001	M	Molar	9	15	Renal Ca	CAKI-1	90.3852
48	830926	2106OS4	Mon Jun	185	S	1	D	0.00001	M	Molar	9	16	Renal Ca	RXF 393	90.0402
49	830926	2106OS4	Mon Jun	185	S	1	D	0.00001	M	Molar	9	8	Renal Ca	SN12C	104.673
50	830926	2106OS4	Mon Jun	185	S	1	D	0.00001	M	Molar	9	24	Renal Ca	TK-10	137.514
51	830926	2106OS4	Mon Jun	185	S	1	D	0.00001	M	Molar	9	4	Renal Ca	UO-31	87.0419
52	830926	2106OS4	Mon Jun	185	S	1	D	0.00001	M	Molar	11	1	Prostate	PC-3	106.183
53	830926	2106OS4	Mon Jun	185	S	1	D	0.00001	M	Molar	11	3	Prostate	DU-145	104.565
54	830926	2106OS4	Mon Jun	185	S	1	D	0.00001	M	Molar	5	1	Breast C	MCF7	73.6786
55	830926	2106OS4	Mon Jun	185	S	1	D	0.00001	M	Molar	5	5	Breast C	MDA-MI	100.785
56	830926	2106OS4	Mon Jun	185	S	1	D	0.00001	M	Molar	5	6	Breast C	HS 578T	98.3417
57	830926	2106OS4	Mon Jun	185	S	1	D	0.00001	M	Molar	5	13	Breast C	BT-549	137.37
58	830926	2106OS4	Mon Jun	185	S	1	D	0.00001	M	Molar	5	14	Breast C	T-47D	94.7949
59	830926	2106OS4	Mon Jun	185	S	1	D	0.00001	M	Molar	5	18	Breast C	MDA-MI	106.919

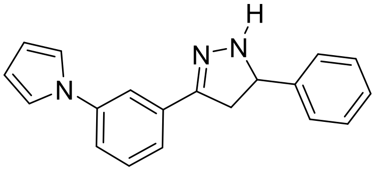
H
N
N
N

6a

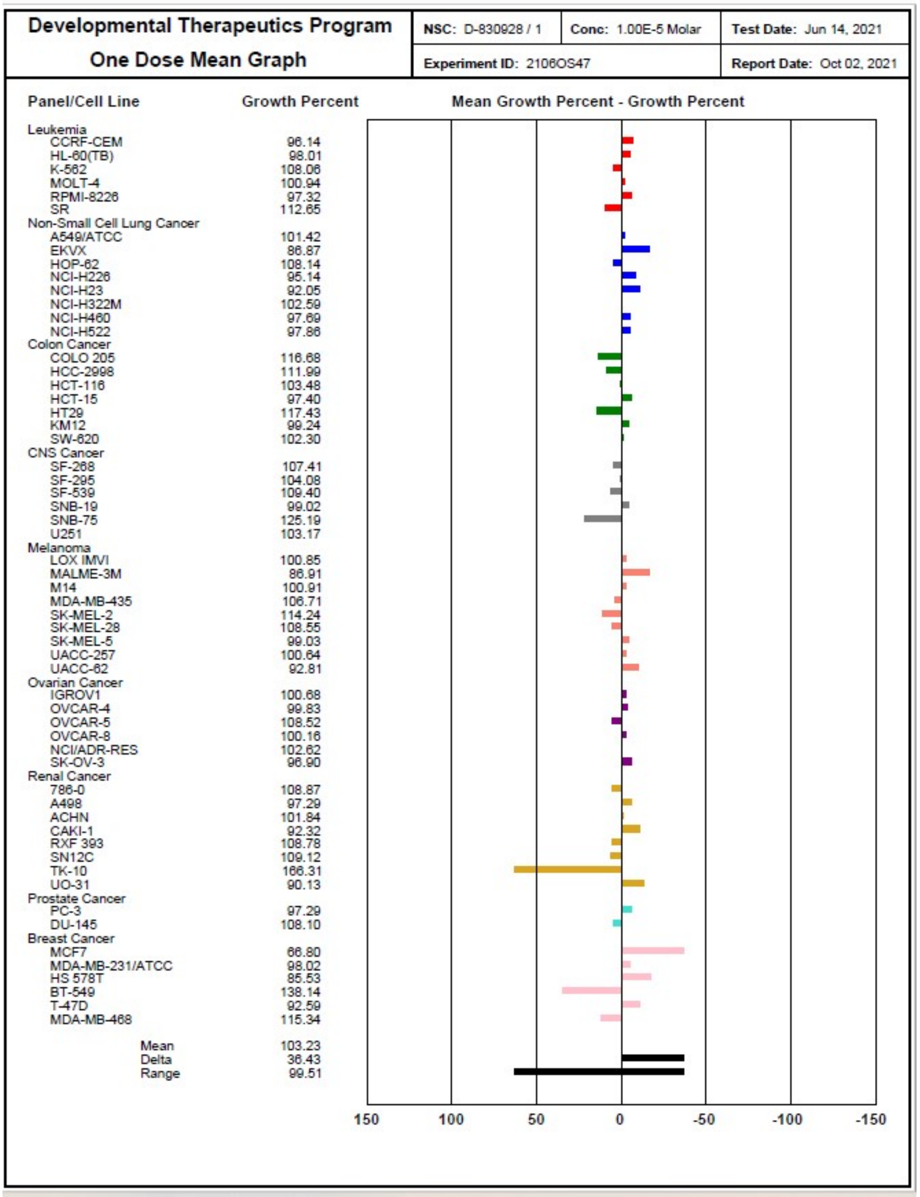
Developmental Therapeutics Program
One Dose Mean Graph
NSC: D-830928 / 1
Conc: 1.00E-5 Molar
Test Date: Jun 14, 2021
Experiment ID: 2106OS47
Report Date: Oct 02, 2021
Panel/Cell Line
Growth Percent
Mean Growth Percent - Growth Percent
Leukemia
CCRF-CEM 96.14
HL-60(TB) 98.01
K-562 108.06
MOLT-4 100.94
RPMI-8226 97.32
SR 112.65
Non-Small Cell Lung Cancer
A549/ATCC 101.42
EKVX 86.87
HOP-62 108.14
NCI-H226 95.14
NCI-H23 92.05
NCI-H322M 102.59
NCI-H460 97.69
NCI-H522 97.86
Colon Cancer
COLO 205 116.68
HCC-2998 111.99
HCT-116 103.48
HCT-15 97.40
HT29 117.43
KM12 99.24
SW-620 102.30
CNS Cancer
SF-268 107.41
SF-295 104.08
SF-539 109.40
SNB-19 99.02
SNB-75 125.19
U251 103.17
Melanoma
LOX IMVI 100.85
MALME-3M 86.91
M14 100.91
MDA-MB-435 106.71
SK-MEL-2 114.24
SK-MEL-28 108.55
SK-MEL-5 99.03
UACC-257 100.64
UACC-62 92.81
Ovarian Cancer
IGROV1 100.68
OVCAR-4 99.83
OVCAR-5 108.52
OVCAR-8 100.16
NCI/ADR-RES 102.62
SK-OV-3 96.90
Renal Cancer
786-0 108.87
A498 97.29
ACHN 101.84
CAKI-1 92.32
RXF 393 108.78
SN12C 109.12
TK-10 166.31
UO-31 90.13
Prostate Cancer
PC-3 97.29
DU-145 108.10
Breast Cancer
MCF7 66.80
MDA-MB-231/ATCC 98.02
HS 578T 85.53
BT-549 138.14
T-47D 92.59
MDA-MB-468 115.34
Mean 103.23
Delta 36.43
Range 99.51
150
100
50
0
-50
-100
-150

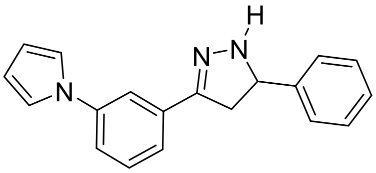

6a

A1 | NSC

	A	B	C	D	E	F	G	H	I	J	K	L	M	N	O
1	NSC	EXPID	PLANDA	TESTSE	PREFIX	SAMPLE	DISCREI	CONC	CONCUI	CONCUI	PANELN	CELLNB	PANELN	CELLNA	GIPRCNT
2	830928	2106OS4	Mon Jun	205	S	1	D	0.00001	M	Molar	7	3	Leukemi	CCRF-C	96.1415
3	830928	2106OS4	Mon Jun	205	S	1	D	0.00001	M	Molar	7	8	Leukemi	HL-60(TE	98.0138
4	830928	2106OS4	Mon Jun	205	S	1	D	0.00001	M	Molar	7	5	Leukemi	K-562	108.064
5	830928	2106OS4	Mon Jun	205	S	1	D	0.00001	M	Molar	7	6	Leukemi	MOLT-4	100.937
6	830928	2106OS4	Mon Jun	205	S	1	D	0.00001	M	Molar	7	10	Leukemi	RPMI-82	97.3225
7	830928	2106OS4	Mon Jun	205	S	1	D	0.00001	M	Molar	7	19	Leukemi	SR	112.651
8	830928	2106OS4	Mon Jun	205	S	1	D	0.00001	M	Molar	1	4	Non-Sm	A549/AT	101.416
9	830928	2106OS4	Mon Jun	205	S	1	D	0.00001	M	Molar	1	8	Non-Sm	EKVX	86.8747
10	830928	2106OS4	Mon Jun	205	S	1	D	0.00001	M	Molar	1	26	Non-Sm	HOP-62	108.135
11	830928	2106OS4	Mon Jun	205	S	1	D	0.00001	M	Molar	1	13	Non-Sm	NCI-H22	95.1444
12	830928	2106OS4	Mon Jun	205	S	1	D	0.00001	M	Molar	1	1	Non-Sm	NCI-H23	92.0535
13	830928	2106OS4	Mon Jun	205	S	1	D	0.00001	M	Molar	1	17	Non-Sm	NCI-H32	102.589
14	830928	2106OS4	Mon Jun	205	S	1	D	0.00001	M	Molar	1	21	Non-Sm	NCI-H46	97.6928
15	830928	2106OS4	Mon Jun	205	S	1	D	0.00001	M	Molar	1	3	Non-Sm	NCI-H52	97.8566
16	830928	2106OS4	Mon Jun	205	S	1	D	0.00001	M	Molar	4	10	Colon C	COLO 20	116.683
17	830928	2106OS4	Mon Jun	205	S	1	D	0.00001	M	Molar	4	2	Colon C	HCC-29	111.995
18	830928	2106OS4	Mon Jun	205	S	1	D	0.00001	M	Molar	4	3	Colon C	HCT-116	103.475
19	830928	2106OS4	Mon Jun	205	S	1	D	0.00001	M	Molar	4	15	Colon C	HCT-15	97.3988
20	830928	2106OS4	Mon Jun	205	S	1	D	0.00001	M	Molar	4	1	Colon C	HT29	117.431
21	830928	2106OS4	Mon Jun	205	S	1	D	0.00001	M	Molar	4	17	Colon C	KM12	99.2389
22	830928	2106OS4	Mon Jun	205	S	1	D	0.00001	M	Molar	4	9	Colon C	SW-620	102.303
23	830928	2106OS4	Mon Jun	205	S	1	D	0.00001	M	Molar	12	14	CNS Car	SF-268	107.407
24	830928	2106OS4	Mon Jun	205	S	1	D	0.00001	M	Molar	12	15	CNS Car	SF-295	104.081
25	830928	2106OS4	Mon Jun	205	S	1	D	0.00001	M	Molar	12	16	CNS Car	SF-539	109.399
26	830928	2106OS4	Mon Jun	205	S	1	D	0.00001	M	Molar	12	2	CNS Car	SNB-19	99.0181
27	830928	2106OS4	Mon Jun	205	S	1	D	0.00001	M	Molar	12	5	CNS Car	SNB-75	125.195
28	830928	2106OS4	Mon Jun	205	S	1	D	0.00001	M	Molar	12	9	CNS Car	U251	103.174
29	830928	2106OS4	Mon Jun	205	S	1	D	0.00001	M	Molar	10	1	Melanon	LOX IMV	100.845
30	830928	2106OS4	Mon Jun	205	S	1	D	0.00001	M	Molar	10	2	Melanon	MALME	86.9099
31	830928	2106OS4	Mon Jun	205	S	1	D	0.00001	M	Molar	10	14	Melanon	M14	100.907
32	830928	2106OS4	Mon Jun	205	S	1	D	0.00001	M	Molar	5	11	Melanon	MDA-MI	106.711
33	830928	2106OS4	Mon Jun	205	S	1	D	0.00001	M	Molar	10	5	Melanon	SK-MEL-	114.242
34	830928	2106OS4	Mon Jun	205	S	1	D	0.00001	M	Molar	10	8	Melanon	SK-MEL-	108.545
35	830928	2106OS4	Mon Jun	205	S	1	D	0.00001	M	Molar	10	7	Melanon	SK-MEL-	99.0317
36	830928	2106OS4	Mon Jun	205	S	1	D	0.00001	M	Molar	10	21	Melanon	UACC-2	100.641
37	830928	2106OS4	Mon Jun	205	S	1	D	0.00001	M	Molar	10	20	Melanon	UACC-6	92.8106
38	830928	2106OS4	Mon Jun	205	S	1	D	0.00001	M	Molar	6	10	Ovarian (	IGROV1	100.676
39	830928	2106OS4	Mon Jun	205	S	1	D	0.00001	M	Molar	6	2	Ovarian (	OVCAR-	99.8291
40	830928	2106OS4	Mon Jun	205	S	1	D	0.00001	M	Molar	6	3	Ovarian (	OVCAR-	108.524
41	830928	2106OS4	Mon Jun	205	S	1	D	0.00001	M	Molar	6	5	Ovarian (	OVCAR-	100.159
42	830928	2106OS4	Mon Jun	205	S	1	D	0.00001	M	Molar	5	2	Ovarian (	NCI/ADF	102.623
43	830928	2106OS4	Mon Jun	205	S	1	D	0.00001	M	Molar	6	11	Ovarian (	SK-OV-3	96.9025
44	830928	2106OS4	Mon Jun	205	S	1	D	0.00001	M	Molar	9	18	Renal Ca	786-0	108.867
45	830928	2106OS4	Mon Jun	205	S	1	D	0.00001	M	Molar	9	13	Renal Ca	A498	97.2903
46	830928	2106OS4	Mon Jun	205	S	1	D	0.00001	M	Molar	9	23	Renal Ca	ACHN	101.838
47	830928	2106OS4	Mon Jun	205	S	1	D	0.00001	M	Molar	9	15	Renal Ca	CAKI-1	92.3216
48	830928	2106OS4	Mon Jun	205	S	1	D	0.00001	M	Molar	9	16	Renal Ca	RXF 393	108.777
49	830928	2106OS4	Mon Jun	205	S	1	D	0.00001	M	Molar	9	8	Renal Ca	SN12C	109.118
50	830928	2106OS4	Mon Jun	205	S	1	D	0.00001	M	Molar	9	24	Renal Ca	TK-10	166.308
51	830928	2106OS4	Mon Jun	205	S	1	D	0.00001	M	Molar	9	4	Renal Ca	UO-31	90.1338
52	830928	2106OS4	Mon Jun	205	S	1	D	0.00001	M	Molar	11	1	Prostate	PC-3	97.2884
53	830928	2106OS4	Mon Jun	205	S	1	D	0.00001	M	Molar	11	3	Prostate	DU-145	108.1
54	830928	2106OS4	Mon Jun	205	S	1	D	0.00001	M	Molar	5	1	Breast C	MCF7	66.8048
55	830928	2106OS4	Mon Jun	205	S	1	D	0.00001	M	Molar	5	5	Breast C	MDA-MI	98.0153
56	830928	2106OS4	Mon Jun	205	S	1	D	0.00001	M	Molar	5	6	Breast C	HS 578T	85.5319
57	830928	2106OS4	Mon Jun	205	S	1	D	0.00001	M	Molar	5	13	Breast C	BT-549	138.142
58	830928	2106OS4	Mon Jun	205	S	1	D	0.00001	M	Molar	5	14	Breast C	T-47D	92.5903
59	830928	2106OS4	Mon Jun	205	S	1	D	0.00001	M	Molar	5	18	Breast C	MDA-MI	115.337
60															
61															
62															
63															
64															
65															
66															
67															

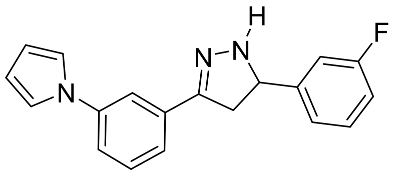

6b

Developmental Therapeutics Program

One Dose Mean Graph

NSC: D-830929 / 1 | **Conc:** 1.00E-5 Molar | **Test Date:** Jun 14, 2021

Experiment ID: 2106OS47 | **Report Date:** Oct 02, 2021

Panel/Cell Line	Growth Percent
Leukemia	
CCRF-CEM	101.59
HL-60(TB)	108.02
K-562	98.10
MOLT-4	130.77
RPMI-8226	97.17
Non-Small Cell Lung Cancer	
A549/ATCC	101.42
EKVX	88.07
HOP-62	106.95
NCI-H226	90.52
NCI-H23	90.14
NCI-H322M	96.92
NCI-H460	95.37
NCI-H522	97.93
Colon Cancer	
COLO 205	111.59
HCC-2998	108.54
HCT-116	102.68
HCT-15	99.90
HT29	109.22
KM12	101.61
SW-620	98.43
CNS Cancer	
SF-268	109.07
SF-295	111.41
SF-539	104.00
SNB-19	98.66
SNB-75	113.78
U251	99.84
Melanoma	
LOX IMVI	94.08
MALME-3M	89.96
M14	96.45
MDA-MB-435	107.80
SK-MEL-2	104.61
SK-MEL-28	110.85
SK-MEL-5	99.50
UACC-257	106.57
UACC-62	92.57
Ovarian Cancer	
IGROV1	94.42
OVCAR-4	105.00
OVCAR-5	114.43
OVCAR-8	99.80
NCI/ADR-RES	106.04
SK-OV-3	95.59
Renal Cancer	
786-0	106.41
A498	100.85
ACHN	100.30
CAKI-1	88.84
RXF 393	95.80
SN12C	103.56
TK-10	192.89
UO-31	77.59
Prostate Cancer	
PC-3	95.14
DU-145	109.19
Breast Cancer	
MCF7	59.13
MDA-MB-231/ATCC	95.66
HS 578T	92.58
BT-549	144.51
T-47D	87.95
MDA-MB-468	110.50
Mean	102.64
Delta	43.51
Range	133.76

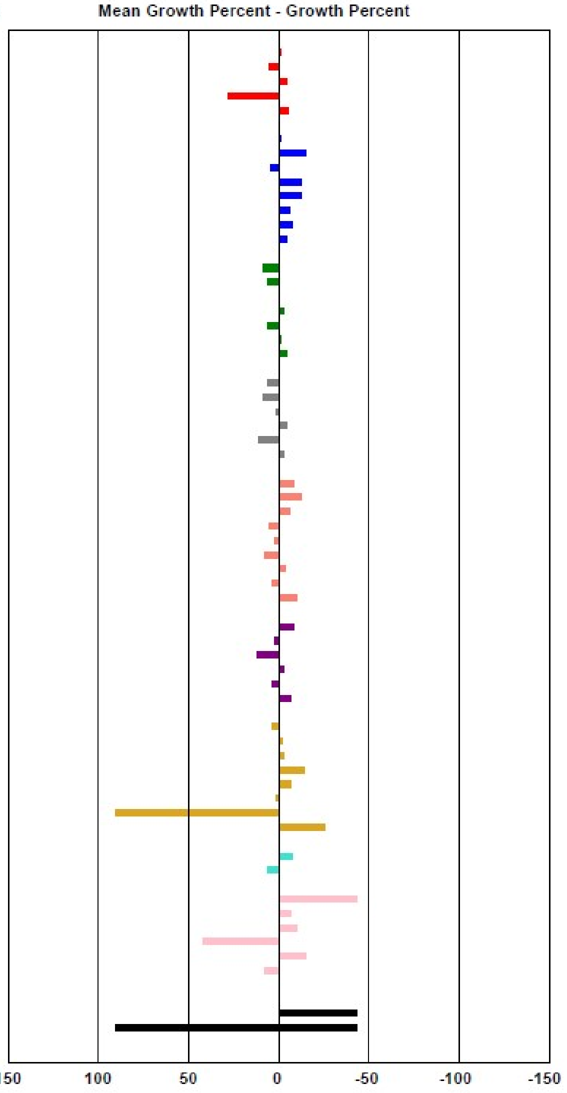

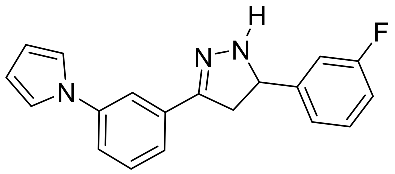

6b

A1 NSC

	A	B	C	D	E	F	G	H	I	J	K	L	M	N	O	P
1	NSC	EXPID	PLANDA	TESTSE	PREFIX	SAMPLE	DISCREI	CONC	CONCUI	CONCUI	PANELN	CELLNB	PANELN	CELLNA	GIPRCNT	
2	830929	2106OS4	Mon Jun	215	S	1	D	0.00001	M	Molar	7	3	Leukemi	CCRF-C	101.591	
3	830929	2106OS4	Mon Jun	215	S	1	D	0.00001	M	Molar	7	8	Leukemi	HL-60(TE	108.02	
4	830929	2106OS4	Mon Jun	215	S	1	D	0.00001	M	Molar	7	5	Leukemi	K-562	98.0972	
5	830929	2106OS4	Mon Jun	215	S	1	D	0.00001	M	Molar	7	6	Leukemi	MOLT-4	130.77	
6	830929	2106OS4	Mon Jun	215	S	1	D	0.00001	M	Molar	7	10	Leukemi	RPMI-82	97.1704	
7	830929	2106OS4	Mon Jun	215	S	1	D	0.00001	M	Molar	1	4	Non-Sm	A549/AT	101.418	
8	830929	2106OS4	Mon Jun	215	S	1	D	0.00001	M	Molar	1	8	Non-Sm	EKVX	88.0705	
9	830929	2106OS4	Mon Jun	215	S	1	D	0.00001	M	Molar	1	26	Non-Sm	HOP-62	106.952	
10	830929	2106OS4	Mon Jun	215	S	1	D	0.00001	M	Molar	1	13	Non-Sm	NCI-H22	90.5163	
11	830929	2106OS4	Mon Jun	215	S	1	D	0.00001	M	Molar	1	1	Non-Sm	NCI-H23	90.143	
12	830929	2106OS4	Mon Jun	215	S	1	D	0.00001	M	Molar	1	17	Non-Sm	NCI-H32	96.9243	
13	830929	2106OS4	Mon Jun	215	S	1	D	0.00001	M	Molar	1	21	Non-Sm	NCI-H46	95.3749	
14	830929	2106OS4	Mon Jun	215	S	1	D	0.00001	M	Molar	1	3	Non-Sm	NCI-H52	97.9319	
15	830929	2106OS4	Mon Jun	215	S	1	D	0.00001	M	Molar	4	10	Colon C	COLO 20	111.592	
16	830929	2106OS4	Mon Jun	215	S	1	D	0.00001	M	Molar	4	2	Colon C	HCC-29	108.537	
17	830929	2106OS4	Mon Jun	215	S	1	D	0.00001	M	Molar	4	3	Colon C	HCT-116	102.679	
18	830929	2106OS4	Mon Jun	215	S	1	D	0.00001	M	Molar	4	15	Colon C	HCT-15	99.8955	
19	830929	2106OS4	Mon Jun	215	S	1	D	0.00001	M	Molar	4	1	Colon C	HT29	109.216	
20	830929	2106OS4	Mon Jun	215	S	1	D	0.00001	M	Molar	4	17	Colon C	KM12	101.607	
21	830929	2106OS4	Mon Jun	215	S	1	D	0.00001	M	Molar	4	9	Colon C	SW-620	98.4254	
22	830929	2106OS4	Mon Jun	215	S	1	D	0.00001	M	Molar	12	14	CNS Car	SF-268	109.066	
23	830929	2106OS4	Mon Jun	215	S	1	D	0.00001	M	Molar	12	15	CNS Car	SF-295	111.414	
24	830929	2106OS4	Mon Jun	215	S	1	D	0.00001	M	Molar	12	16	CNS Car	SF-539	104.004	
25	830929	2106OS4	Mon Jun	215	S	1	D	0.00001	M	Molar	12	2	CNS Car	SNB-19	98.6598	
26	830929	2106OS4	Mon Jun	215	S	1	D	0.00001	M	Molar	12	5	CNS Car	SNB-75	113.781	
27	830929	2106OS4	Mon Jun	215	S	1	D	0.00001	M	Molar	12	9	CNS Car	U251	99.8386	
28	830929	2106OS4	Mon Jun	215	S	1	D	0.00001	M	Molar	10	1	Melanon	LOX IMV	94.0776	
29	830929	2106OS4	Mon Jun	215	S	1	D	0.00001	M	Molar	10	2	Melanon	MALME	89.959	
30	830929	2106OS4	Mon Jun	215	S	1	D	0.00001	M	Molar	10	14	Melanon	M14	96.4473	
31	830929	2106OS4	Mon Jun	215	S	1	D	0.00001	M	Molar	5	11	Melanon	MDA-MI	107.805	
32	830929	2106OS4	Mon Jun	215	S	1	D	0.00001	M	Molar	10	5	Melanon	SK-MEL-	104.612	
33	830929	2106OS4	Mon Jun	215	S	1	D	0.00001	M	Molar	10	8	Melanon	SK-MEL-	110.854	
34	830929	2106OS4	Mon Jun	215	S	1	D	0.00001	M	Molar	10	7	Melanon	SK-MEL-	99.4993	
35	830929	2106OS4	Mon Jun	215	S	1	D	0.00001	M	Molar	10	21	Melanon	UACC-2	106.575	
36	830929	2106OS4	Mon Jun	215	S	1	D	0.00001	M	Molar	10	20	Melanon	UACC-6	92.5692	
37	830929	2106OS4	Mon Jun	215	S	1	D	0.00001	M	Molar	6	10	Ovarian (	IGROV1	94.4243	
38	830929	2106OS4	Mon Jun	215	S	1	D	0.00001	M	Molar	6	2	Ovarian (	OVCAR-	104.997	
39	830929	2106OS4	Mon Jun	215	S	1	D	0.00001	M	Molar	6	3	Ovarian (	OVCAR-	114.425	
40	830929	2106OS4	Mon Jun	215	S	1	D	0.00001	M	Molar	6	5	Ovarian (	OVCAR-	99.8042	
41	830929	2106OS4	Mon Jun	215	S	1	D	0.00001	M	Molar	5	2	Ovarian (	NCI/ADF	106.044	
42	830929	2106OS4	Mon Jun	215	S	1	D	0.00001	M	Molar	6	11	Ovarian (	SK-OV-3	95.5867	
43	830929	2106OS4	Mon Jun	215	S	1	D	0.00001	M	Molar	9	18	Renal Ca	786-0	106.412	
44	830929	2106OS4	Mon Jun	215	S	1	D	0.00001	M	Molar	9	13	Renal Ca	A498	100.849	
45	830929	2106OS4	Mon Jun	215	S	1	D	0.00001	M	Molar	9	23	Renal Ca	ACHN	100.299	
46	830929	2106OS4	Mon Jun	215	S	1	D	0.00001	M	Molar	9	15	Renal Ca	CAKI-1	88.8371	
47	830929	2106OS4	Mon Jun	215	S	1	D	0.00001	M	Molar	9	16	Renal Ca	RXF 393	95.7984	
48	830929	2106OS4	Mon Jun	215	S	1	D	0.00001	M	Molar	9	8	Renal Ca	SN12C	103.561	
49	830929	2106OS4	Mon Jun	215	S	1	D	0.00001	M	Molar	9	24	Renal Ca	TK-10	192.894	
50	830929	2106OS4	Mon Jun	215	S	1	D	0.00001	M	Molar	9	4	Renal Ca	UO-31	77.5936	
51	830929	2106OS4	Mon Jun	215	S	1	D	0.00001	M	Molar	11	1	Prostate	PC-3	95.1358	
52	830929	2106OS4	Mon Jun	215	S	1	D	0.00001	M	Molar	11	3	Prostate	DU-145	109.192	
53	830929	2106OS4	Mon Jun	215	S	1	D	0.00001	M	Molar	5	1	Breast C	MCF7	59.1268	
54	830929	2106OS4	Mon Jun	215	S	1	D	0.00001	M	Molar	5	5	Breast C	MDA-MI	95.6632	
55	830929	2106OS4	Mon Jun	215	S	1	D	0.00001	M	Molar	5	6	Breast C	HS 578T	92.5782	
56	830929	2106OS4	Mon Jun	215	S	1	D	0.00001	M	Molar	5	13	Breast C	BT-549	144.513	
57	830929	2106OS4	Mon Jun	215	S	1	D	0.00001	M	Molar	5	14	Breast C	T-47D	87.9525	
58	830929	2106OS4	Mon Jun	215	S	1	D	0.00001	M	Molar	5	18	Breast C	MDA-MI	110.502	
59																
60																
61																
62																
63																
64																
65																
66																

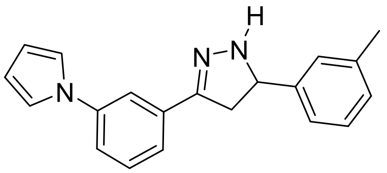

6c

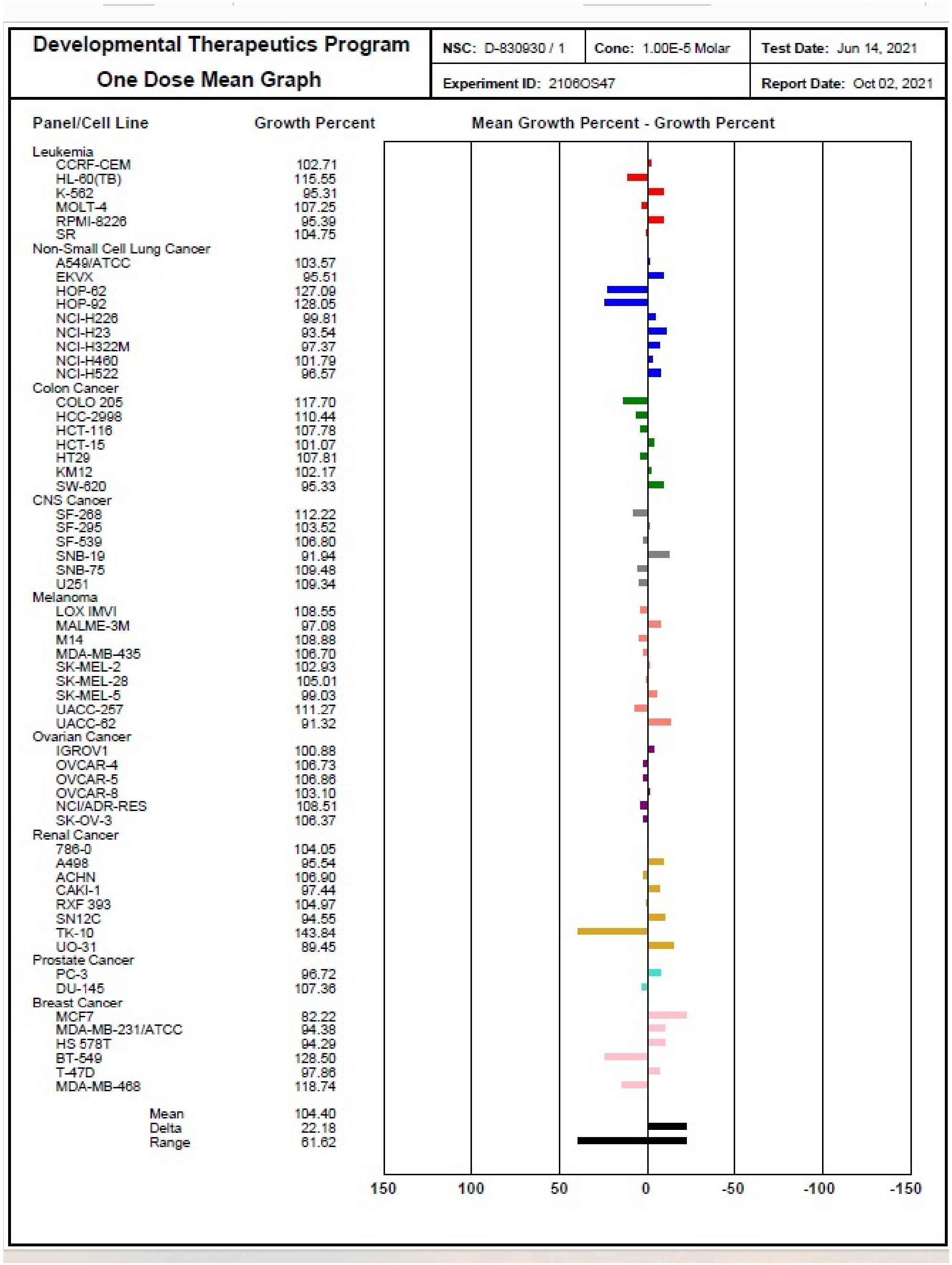

Developmental Therapeutics Program

One Dose Mean Graph

NSC: D-830930 / 1 | Conc: 1.00E-5 Molar | Test Date: Jun 14, 2021

Experiment ID: 2106OS47 | Report Date: Oct 02, 2021

Panel/Cell Line	Growth Percent
Leukemia	
CCRF-CEM	102.71
HL-60(TB)	115.55
K-562	95.31
MOLT-4	107.25
RPMI-8226	95.39
SR	104.75
Non-Small Cell Lung Cancer	
A549/ATCC	103.57
EKVX	95.51
HOP-62	127.09
HOP-92	128.05
NCI-H226	99.81
NCI-H23	93.54
NCI-H322M	97.37
NCI-H460	101.79
NCI-H522	96.57
Colon Cancer	
COLO 205	117.70
HCC-2998	110.44
HCT-116	107.78
HCT-15	101.07
HT29	107.81
KM12	102.17
SW-620	95.33
CNS Cancer	
SF-268	112.22
SF-295	103.52
SF-539	106.80
SNB-19	91.94
SNB-75	109.48
U251	109.34
Melanoma	
LOX IMVI	108.55
MALME-3M	97.08
M14	108.88
MDA-MB-435	106.70
SK-MEL-2	102.93
SK-MEL-28	105.01
SK-MEL-5	99.03
UACC-257	111.27
UACC-62	91.32
Ovarian Cancer	
IGROV1	100.88
OVCAR-4	106.73
OVCAR-5	106.86
OVCAR-8	103.10
NCI/ADR-RES	108.51
SK-OV-3	106.37
Renal Cancer	
786-0	104.05
A498	95.54
ACHN	106.90
CAKI-1	97.44
RXF 393	104.97
SN12C	94.55
TK-10	143.84
UO-31	89.45
Prostate Cancer	
PC-3	96.72
DU-145	107.36
Breast Cancer	
MCF7	82.22
MDA-MB-231/ATCC	94.38
HS 578T	94.29
BT-549	128.50
T-47D	97.86
MDA-MB-468	118.74
Mean	104.40
Delta	22.18
Range	61.62

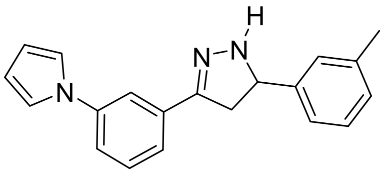

6c

	NSC	EXPID	PLANDA	TESTSE	PREFIX	SAMPLE	DISCREI	CONC	CONCUI	CONCUI	PANELN	CELLNB	PANELN	CELLNA	GIPRCNT
2	830930	2106OS4	Mon Jun	225	S	1	D	0.00001	M	Molar	7	3	Leukemi	CCRF-C	102.71
3	830930	2106OS4	Mon Jun	225	S	1	D	0.00001	M	Molar	7	8	Leukemi	HL-60(TE	115.551
4	830930	2106OS4	Mon Jun	225	S	1	D	0.00001	M	Molar	7	5	Leukemi	K-562	95.3082
5	830930	2106OS4	Mon Jun	225	S	1	D	0.00001	M	Molar	7	6	Leukemi	MOLT-4	107.247
6	830930	2106OS4	Mon Jun	225	S	1	D	0.00001	M	Molar	7	10	Leukemi	RPMI-82	95.3874
7	830930	2106OS4	Mon Jun	225	S	1	D	0.00001	M	Molar	7	19	Leukemi	SR	104.747
8	830930	2106OS4	Mon Jun	225	S	1	D	0.00001	M	Molar	1	4	Non-Sm	A549/AT	103.569
9	830930	2106OS4	Mon Jun	225	S	1	D	0.00001	M	Molar	1	8	Non-Sm	EKVX	95.5112
10	830930	2106OS4	Mon Jun	225	S	1	D	0.00001	M	Molar	1	26	Non-Sm	HOP-62	127.088
11	830930	2106OS4	Mon Jun	225	S	1	D	0.00001	M	Molar	1	29	Non-Sm	HOP-92	128.047
12	830930	2106OS4	Mon Jun	225	S	1	D	0.00001	M	Molar	1	13	Non-Sm	NCI-H22	99.8102
13	830930	2106OS4	Mon Jun	225	S	1	D	0.00001	M	Molar	1	1	Non-Sm	NCI-H23	93.5414
14	830930	2106OS4	Mon Jun	225	S	1	D	0.00001	M	Molar	1	17	Non-Sm	NCI-H32	97.3654
15	830930	2106OS4	Mon Jun	225	S	1	D	0.00001	M	Molar	1	21	Non-Sm	NCI-H46	101.789
16	830930	2106OS4	Mon Jun	225	S	1	D	0.00001	M	Molar	1	3	Non-Sm	NCI-H52	96.566
17	830930	2106OS4	Mon Jun	225	S	1	D	0.00001	M	Molar	4	10	Colon C	COLO 20	117.702
18	830930	2106OS4	Mon Jun	225	S	1	D	0.00001	M	Molar	4	2	Colon C	HCC-29	110.439
19	830930	2106OS4	Mon Jun	225	S	1	D	0.00001	M	Molar	4	3	Colon C	HCT-116	107.779
20	830930	2106OS4	Mon Jun	225	S	1	D	0.00001	M	Molar	4	15	Colon C	HCT-15	101.075
21	830930	2106OS4	Mon Jun	225	S	1	D	0.00001	M	Molar	4	1	Colon C	HT29	107.811
22	830930	2106OS4	Mon Jun	225	S	1	D	0.00001	M	Molar	4	17	Colon C	KM12	102.172
23	830930	2106OS4	Mon Jun	225	S	1	D	0.00001	M	Molar	4	9	Colon C	SW-620	95.3302
24	830930	2106OS4	Mon Jun	225	S	1	D	0.00001	M	Molar	12	14	CNS Car	SF-268	112.222
25	830930	2106OS4	Mon Jun	225	S	1	D	0.00001	M	Molar	12	15	CNS Car	SF-295	103.52
26	830930	2106OS4	Mon Jun	225	S	1	D	0.00001	M	Molar	12	16	CNS Car	SF-539	106.803
27	830930	2106OS4	Mon Jun	225	S	1	D	0.00001	M	Molar	12	2	CNS Car	SNB-19	91.9357
28	830930	2106OS4	Mon Jun	225	S	1	D	0.00001	M	Molar	12	5	CNS Car	SNB-75	109.48
29	830930	2106OS4	Mon Jun	225	S	1	D	0.00001	M	Molar	12	9	CNS Car	U251	109.342
30	830930	2106OS4	Mon Jun	225	S	1	D	0.00001	M	Molar	10	1	Melanon	LOX IMV	108.552
31	830930	2106OS4	Mon Jun	225	S	1	D	0.00001	M	Molar	10	2	Melanon	MALME	97.076
32	830930	2106OS4	Mon Jun	225	S	1	D	0.00001	M	Molar	10	14	Melanon	M14	108.876
33	830930	2106OS4	Mon Jun	225	S	1	D	0.00001	M	Molar	5	11	Melanon	MDA-MI	106.698
34	830930	2106OS4	Mon Jun	225	S	1	D	0.00001	M	Molar	10	5	Melanon	SK-MEL-	102.926
35	830930	2106OS4	Mon Jun	225	S	1	D	0.00001	M	Molar	10	8	Melanon	SK-MEL-	105.014
36	830930	2106OS4	Mon Jun	225	S	1	D	0.00001	M	Molar	10	7	Melanon	SK-MEL-	99.026
37	830930	2106OS4	Mon Jun	225	S	1	D	0.00001	M	Molar	10	21	Melanon	UACC-2	111.269
38	830930	2106OS4	Mon Jun	225	S	1	D	0.00001	M	Molar	10	20	Melanon	UACC-6	91.3178
39	830930	2106OS4	Mon Jun	225	S	1	D	0.00001	M	Molar	6	10	Ovarian (	IGROV1	100.882
40	830930	2106OS4	Mon Jun	225	S	1	D	0.00001	M	Molar	6	2	Ovarian (	OVCAR-	106.726
41	830930	2106OS4	Mon Jun	225	S	1	D	0.00001	M	Molar	6	3	Ovarian (	OVCAR-	106.862
42	830930	2106OS4	Mon Jun	225	S	1	D	0.00001	M	Molar	6	5	Ovarian (	OVCAR-	103.097
43	830930	2106OS4	Mon Jun	225	S	1	D	0.00001	M	Molar	5	2	Ovarian (	NCI/ADF	108.515
44	830930	2106OS4	Mon Jun	225	S	1	D	0.00001	M	Molar	6	11	Ovarian (	SK-OV-3	106.372
45	830930	2106OS4	Mon Jun	225	S	1	D	0.00001	M	Molar	9	18	Renal Ca	786-0	104.053
46	830930	2106OS4	Mon Jun	225	S	1	D	0.00001	M	Molar	9	13	Renal Ca	A498	95.5414
47	830930	2106OS4	Mon Jun	225	S	1	D	0.00001	M	Molar	9	23	Renal Ca	ACHN	106.903
48	830930	2106OS4	Mon Jun	225	S	1	D	0.00001	M	Molar	9	15	Renal Ca	CAKI-1	97.4382
49	830930	2106OS4	Mon Jun	225	S	1	D	0.00001	M	Molar	9	16	Renal Ca	RXF 393	104.967
50	830930	2106OS4	Mon Jun	225	S	1	D	0.00001	M	Molar	9	8	Renal Ca	SN12C	94.5453
51	830930	2106OS4	Mon Jun	225	S	1	D	0.00001	M	Molar	9	24	Renal Ca	TK-10	143.836
52	830930	2106OS4	Mon Jun	225	S	1	D	0.00001	M	Molar	9	4	Renal Ca	UO-31	89.4528
53	830930	2106OS4	Mon Jun	225	S	1	D	0.00001	M	Molar	11	1	Prostate	PC-3	96.7161
54	830930	2106OS4	Mon Jun	225	S	1	D	0.00001	M	Molar	11	3	Prostate	DU-145	107.355
55	830930	2106OS4	Mon Jun	225	S	1	D	0.00001	M	Molar	5	1	Breast C	MCF7	82.217
56	830930	2106OS4	Mon Jun	225	S	1	D	0.00001	M	Molar	5	5	Breast C	MDA-MI	94.3783
57	830930	2106OS4	Mon Jun	225	S	1	D	0.00001	M	Molar	5	6	Breast C	HS 578T	94.2876
58	830930	2106OS4	Mon Jun	225	S	1	D	0.00001	M	Molar	5	13	Breast C	BT-549	128.503
59	830930	2106OS4	Mon Jun	225	S	1	D	0.00001	M	Molar	5	14	Breast C	T-47D	97.8554
60	830930	2106OS4	Mon Jun	225	S	1	D	0.00001	M	Molar	5	18	Breast C	MDA-MI	118.737

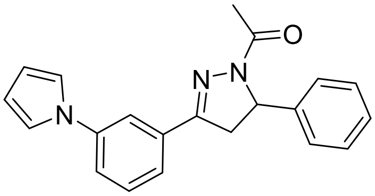

7a

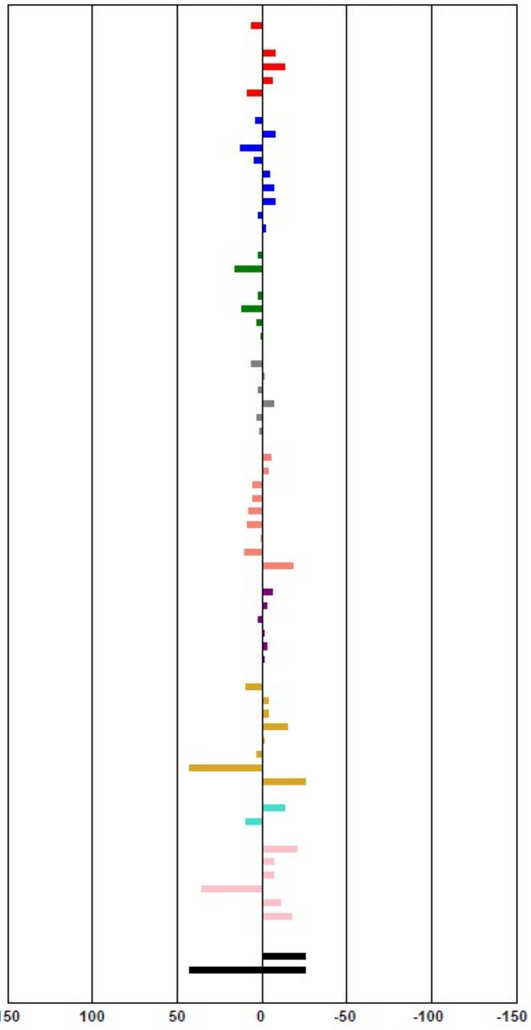

Developmental Therapeutics Program

One Dose Mean Graph

**NSC:** D-830931 / 1	**Conc:** 1.00E-5 Molar
**Test Date:** Jun 14, 2021	**Experiment ID:** 2106OS47
**Report Date:** Oct 02, 2021	

Panel/Cell Line	Growth Percent
**Leukemia**	
CCRF-CEM	102.64
HL-60(TB)	96.19
K-562	89.05
MOLT-4	82.81
RPMI-8226	90.08
SR	104.95
**Non-Small Cell Lung Cancer**	
A549/ATCC	100.35
EKVX	88.50
HOP-62	108.62
HOP-92	100.77
NCI-H226	92.31
NCI-H23	89.89
NCI-H322M	88.83
NCI-H460	98.19
NCI-H522	94.51
**Colon Cancer**	
COLO 205	98.56
HCC-2998	112.06
HCT-116	95.79
HCT-15	98.35
HT29	108.02
KM12	99.46
SW-620	96.83
**CNS Cancer**	
SF-268	102.76
SF-295	94.80
SF-539	98.35
SNB-19	89.69
SNB-75	99.16
U251	97.89
**Melanoma**	
LOX IMVI	91.18
MALME-3M	92.40
M14	101.87
MDA-MB-435	102.07
SK-MEL-2	104.26
SK-MEL-28	105.23
SK-MEL-5	97.02
UACC-257	106.65
UACC-62	78.01
**Ovarian Cancer**	
IGROV1	90.69
OVCAR-4	93.58
OVCAR-5	98.14
OVCAR-8	95.46
NCI/ADR-RES	93.57
SK-OV-3	95.51
**Renal Cancer**	
786-0	105.86
A498	92.64
ACHN	93.05
CAKI-1	81.10
RXF 393	95.21
SN12C	98.94
TK-10	139.21
UO-31	71.06
**Prostate Cancer**	
PC-3	83.32
DU-145	105.52
**Breast Cancer**	
MCF7	75.46
MDA-MB-231/ATCC	89.88
HS 578T	89.37
BT-549	131.30
T-47D	85.39
MDA-MB-468	79.09
Mean	96.30
Delta	25.24
Range	68.15

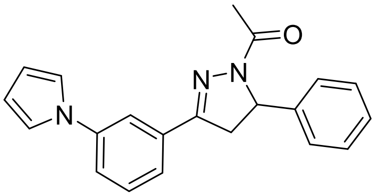

7a

A1 NSC

	A	B	C	D	E	F	G	H	I	J	K	L	M	N	O
1	NSC	EXPID	PLANDA	TESTSE	PREFIX	SAMPLE	DISCREI	CONC	CONCUI	CONCUI	PANELN	CELLNB	PANELN	CELLNA	GIPRCNT
2	830931	2106OS4	Mon Jun	235	S	1	D	0.00001	M	Molar	7	3	Leukemi.	CCRF-C	102.637
3	830931	2106OS4	Mon Jun	235	S	1	D	0.00001	M	Molar	7	8	Leukemi.	HL-60(TE	96.1929
4	830931	2106OS4	Mon Jun	235	S	1	D	0.00001	M	Molar	7	5	Leukemi.	K-562	89.0519
5	830931	2106OS4	Mon Jun	235	S	1	D	0.00001	M	Molar	7	6	Leukemi.	MOLT-4	82.8141
6	830931	2106OS4	Mon Jun	235	S	1	D	0.00001	M	Molar	7	10	Leukemi.	RPMI-82	90.0774
7	830931	2106OS4	Mon Jun	235	S	1	D	0.00001	M	Molar	7	19	Leukemi.	SR	104.948
8	830931	2106OS4	Mon Jun	235	S	1	D	0.00001	M	Molar	1	4	Non-Sm.	A549/AT	100.346
9	830931	2106OS4	Mon Jun	235	S	1	D	0.00001	M	Molar	1	8	Non-Sm.	EKVX	88.5021
10	830931	2106OS4	Mon Jun	235	S	1	D	0.00001	M	Molar	1	26	Non-Sm.	HOP-62	108.617
11	830931	2106OS4	Mon Jun	235	S	1	D	0.00001	M	Molar	1	29	Non-Sm.	HOP-92	100.773
12	830931	2106OS4	Mon Jun	235	S	1	D	0.00001	M	Molar	1	13	Non-Sm.	NCI-H22	92.314
13	830931	2106OS4	Mon Jun	235	S	1	D	0.00001	M	Molar	1	1	Non-Sm.	NCI-H23	89.8904
14	830931	2106OS4	Mon Jun	235	S	1	D	0.00001	M	Molar	1	17	Non-Sm.	NCI-H32	88.8322
15	830931	2106OS4	Mon Jun	235	S	1	D	0.00001	M	Molar	1	21	Non-Sm.	NCI-H46	98.1865
16	830931	2106OS4	Mon Jun	235	S	1	D	0.00001	M	Molar	1	3	Non-Sm.	NCI-H52	94.5114
17	830931	2106OS4	Mon Jun	235	S	1	D	0.00001	M	Molar	4	10	Colon C.	COLO 2C	98.5644
18	830931	2106OS4	Mon Jun	235	S	1	D	0.00001	M	Molar	4	2	Colon C.	HCC-29	112.063
19	830931	2106OS4	Mon Jun	235	S	1	D	0.00001	M	Molar	4	3	Colon C.	HCT-116	95.7905
20	830931	2106OS4	Mon Jun	235	S	1	D	0.00001	M	Molar	4	15	Colon C.	HCT-15	98.3518
21	830931	2106OS4	Mon Jun	235	S	1	D	0.00001	M	Molar	4	1	Colon C.	HT29	108.022
22	830931	2106OS4	Mon Jun	235	S	1	D	0.00001	M	Molar	4	17	Colon C.	KM12	99.462
23	830931	2106OS4	Mon Jun	235	S	1	D	0.00001	M	Molar	4	9	Colon C.	SW-620	96.8321
24	830931	2106OS4	Mon Jun	235	S	1	D	0.00001	M	Molar	12	14	CNS Car	SF-268	102.759
25	830931	2106OS4	Mon Jun	235	S	1	D	0.00001	M	Molar	12	15	CNS Car	SF-295	94.8007
26	830931	2106OS4	Mon Jun	235	S	1	D	0.00001	M	Molar	12	16	CNS Car	SF-539	98.3508
27	830931	2106OS4	Mon Jun	235	S	1	D	0.00001	M	Molar	12	2	CNS Car	SNB-19	89.6868
28	830931	2106OS4	Mon Jun	235	S	1	D	0.00001	M	Molar	12	5	CNS Car	SNB-75	99.1579
29	830931	2106OS4	Mon Jun	235	S	1	D	0.00001	M	Molar	12	9	CNS Car	U251	97.8933
30	830931	2106OS4	Mon Jun	235	S	1	D	0.00001	M	Molar	10	1	Melanon	LOX IMV	91.1841
31	830931	2106OS4	Mon Jun	235	S	1	D	0.00001	M	Molar	10	2	Melanon	MALME	92.3963
32	830931	2106OS4	Mon Jun	235	S	1	D	0.00001	M	Molar	10	14	Melanon	M14	101.868
33	830931	2106OS4	Mon Jun	235	S	1	D	0.00001	M	Molar	5	11	Melanon	MDA-MI	102.072
34	830931	2106OS4	Mon Jun	235	S	1	D	0.00001	M	Molar	10	5	Melanon	SK-MEL-	104.262
35	830931	2106OS4	Mon Jun	235	S	1	D	0.00001	M	Molar	10	8	Melanon	SK-MEL-	105.232
36	830931	2106OS4	Mon Jun	235	S	1	D	0.00001	M	Molar	10	7	Melanon	SK-MEL-	97.0216
37	830931	2106OS4	Mon Jun	235	S	1	D	0.00001	M	Molar	10	21	Melanon	UACC-2	106.65
38	830931	2106OS4	Mon Jun	235	S	1	D	0.00001	M	Molar	10	20	Melanon	UACC-6	78.0123
39	830931	2106OS4	Mon Jun	235	S	1	D	0.00001	M	Molar	6	10	Ovarian (	IGROV1	90.694
40	830931	2106OS4	Mon Jun	235	S	1	D	0.00001	M	Molar	6	2	Ovarian (	OVCAR-	93.5758
41	830931	2106OS4	Mon Jun	235	S	1	D	0.00001	M	Molar	6	3	Ovarian (	OVCAR-	98.1398
42	830931	2106OS4	Mon Jun	235	S	1	D	0.00001	M	Molar	6	5	Ovarian (	OVCAR-	95.4595
43	830931	2106OS4	Mon Jun	235	S	1	D	0.00001	M	Molar	5	2	Ovarian (	NCI/ADF	93.5657
44	830931	2106OS4	Mon Jun	235	S	1	D	0.00001	M	Molar	6	11	Ovarian (	SK-OV-3	95.5087
45	830931	2106OS4	Mon Jun	235	S	1	D	0.00001	M	Molar	9	18	Renal Ca	786-0	105.864
46	830931	2106OS4	Mon Jun	235	S	1	D	0.00001	M	Molar	9	13	Renal Ca	A498	92.643
47	830931	2106OS4	Mon Jun	235	S	1	D	0.00001	M	Molar	9	23	Renal Ca	ACHN	93.053
48	830931	2106OS4	Mon Jun	235	S	1	D	0.00001	M	Molar	9	15	Renal Ca	CAKI-1	81.099
49	830931	2106OS4	Mon Jun	235	S	1	D	0.00001	M	Molar	9	16	Renal Ca	RXF 393	95.2109
50	830931	2106OS4	Mon Jun	235	S	1	D	0.00001	M	Molar	9	8	Renal Ca	SN12C	98.9355
51	830931	2106OS4	Mon Jun	235	S	1	D	0.00001	M	Molar	9	24	Renal Ca	TK-10	139.212
52	830931	2106OS4	Mon Jun	235	S	1	D	0.00001	M	Molar	9	4	Renal Ca	UO-31	71.0602
53	830931	2106OS4	Mon Jun	235	S	1	D	0.00001	M	Molar	11	1	Prostate	PC-3	83.3161
54	830931	2106OS4	Mon Jun	235	S	1	D	0.00001	M	Molar	11	3	Prostate	DU-145	105.521
55	830931	2106OS4	Mon Jun	235	S	1	D	0.00001	M	Molar	5	1	Breast C	MCF7	75.4562
56	830931	2106OS4	Mon Jun	235	S	1	D	0.00001	M	Molar	5	5	Breast C	MDA-MI	89.8794
57	830931	2106OS4	Mon Jun	235	S	1	D	0.00001	M	Molar	5	6	Breast C	HS 578T	89.3731
58	830931	2106OS4	Mon Jun	235	S	1	D	0.00001	M	Molar	5	13	Breast C	BT-549	131.3
59	830931	2106OS4	Mon Jun	235	S	1	D	0.00001	M	Molar	5	14	Breast C	T-47D	85.3929
60	830931	2106OS4	Mon Jun	235	S	1	D	0.00001	M	Molar	5	18	Breast C	MDA-MI	79.089

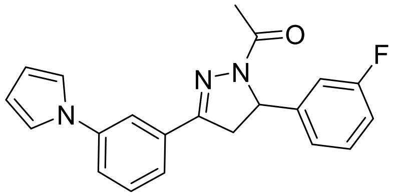

7b

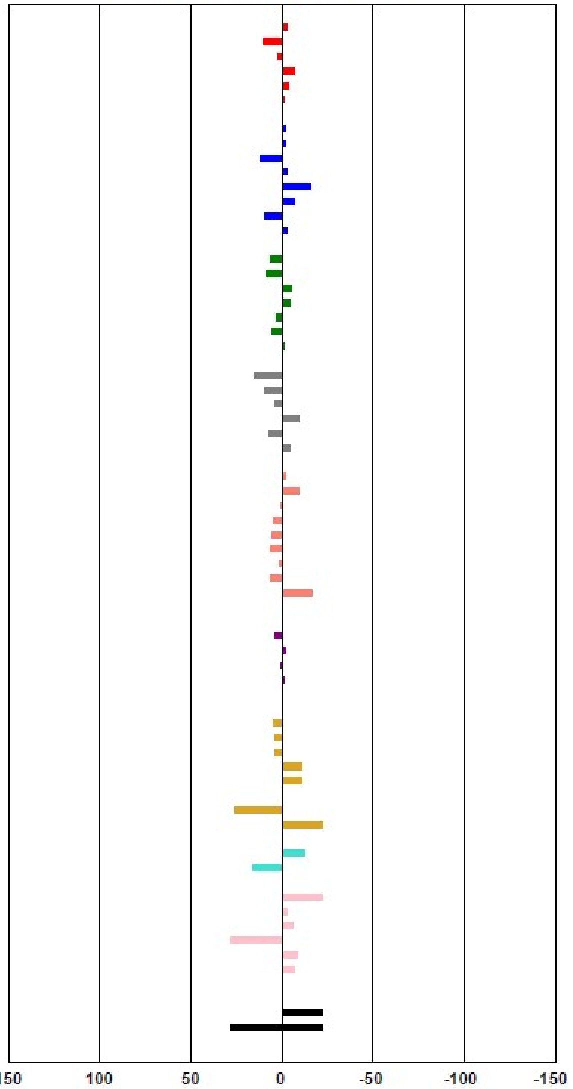

Developmental Therapeutics Program
One Dose Mean Graph

NSC: D-830932 / 1 | Conc: 1.00E-5 Molar | Test Date: Jun 14, 2021
Experiment ID: 2106OS47 | Report Date: Oct 02, 2021

Panel/Cell Line	Growth Percent
Leukemia	
CCRF-CEM	94.39
HL-60(TB)	107.89
K-562	99.87
MOLT-4	90.29
RPMI-8226	93.66
SR	96.21
Non-Small Cell Lung Cancer	
A549/ATCC	95.19
EKVX	95.66
HOP-62	109.06
NCI-H226	94.54
NCI-H23	82.07
NCI-H322M	90.33
NCI-H460	107.05
NCI-H522	94.80
Colon Cancer	
COLO 205	103.62
HCC-2998	105.99
HCT-116	92.49
HCT-15	92.80
HT29	100.23
KM12	103.04
SW-620	95.97
CNS Cancer	
SF-268	112.81
SF-295	106.74
SF-539	101.44
SNB-19	88.51
SNB-75	104.44
U251	93.41
Melanoma	
LOX IMVI	95.53
MALME-3M	88.26
M14	97.61
MDA-MB-435	102.21
SK-MEL-2	102.79
SK-MEL-28	103.19
SK-MEL-5	98.73
UACC-257	103.65
UACC-62	80.96
Ovarian Cancer	
IGROV1	96.92
OVCAR-4	100.80
OVCAR-5	95.78
OVCAR-8	98.19
NCI/ADR-RES	95.89
SK-OV-3	97.26
Renal Cancer	
786-0	102.06
A498	101.26
ACHN	101.28
CAKI-1	86.46
RXF 393	86.15
SN12C	97.12
TK-10	123.10
UO-31	75.37
Prostate Cancer	
PC-3	84.92
DU-145	113.63
Breast Cancer	
MCF7	75.09
MDA-MB-231/ATCC	94.80
HS 578T	91.01
BT-549	125.51
T-47D	89.02
MDA-MB-468	90.36
Mean	97.37
Delta	22.28
Range	50.42

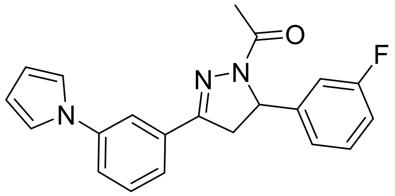

7b

NSC	EXPID	PLANDA	TESTSE	PREFIX	SAMPLE	DISCREI	CONC	CONCUI	CONCUI	PANELN	CELLNB	PANELN	CELLNA	GIPRCNT
830932	2106OS4	Mon Jun	166	S	1	D	0.00001	M	Molar	7	3	Leukemi	CCRF-C	94.3866
830932	2106OS4	Mon Jun	166	S	1	D	0.00001	M	Molar	7	8	Leukemi	HL-60(TE	107.889
830932	2106OS4	Mon Jun	166	S	1	D	0.00001	M	Molar	7	5	Leukemi	K-562	99.8666
830932	2106OS4	Mon Jun	166	S	1	D	0.00001	M	Molar	7	6	Leukemi	MOLT-4	90.2875
830932	2106OS4	Mon Jun	166	S	1	D	0.00001	M	Molar	7	10	Leukemi	RPMI-82	93.6624
830932	2106OS4	Mon Jun	166	S	1	D	0.00001	M	Molar	7	19	Leukemi	SR	96.2066
830932	2106OS4	Mon Jun	166	S	1	D	0.00001	M	Molar	1	4	Non-Sm	A549/AT	95.1897
830932	2106OS4	Mon Jun	166	S	1	D	0.00001	M	Molar	1	8	Non-Sm	EKVX	95.661
830932	2106OS4	Mon Jun	166	S	1	D	0.00001	M	Molar	1	26	Non-Sm	HOP-62	109.064
830932	2106OS4	Mon Jun	166	S	1	D	0.00001	M	Molar	1	13	Non-Sm	NCI-H22	94.5365
830932	2106OS4	Mon Jun	166	S	1	D	0.00001	M	Molar	1	1	Non-Sm	NCI-H23	82.0678
830932	2106OS4	Mon Jun	166	S	1	D	0.00001	M	Molar	1	17	Non-Sm	NCI-H32	90.3277
830932	2106OS4	Mon Jun	166	S	1	D	0.00001	M	Molar	1	21	Non-Sm	NCI-H46	107.053
830932	2106OS4	Mon Jun	166	S	1	D	0.00001	M	Molar	1	3	Non-Sm	NCI-H52	94.7992
830932	2106OS4	Mon Jun	166	S	1	D	0.00001	M	Molar	4	10	Colon C	COLO 20	103.623
830932	2106OS4	Mon Jun	166	S	1	D	0.00001	M	Molar	4	2	Colon C	HCC-29	105.994
830932	2106OS4	Mon Jun	166	S	1	D	0.00001	M	Molar	4	3	Colon C	HCT-116	92.4882
830932	2106OS4	Mon Jun	166	S	1	D	0.00001	M	Molar	4	15	Colon C	HCT-15	92.8002
830932	2106OS4	Mon Jun	166	S	1	D	0.00001	M	Molar	4	1	Colon C	HT29	100.234
830932	2106OS4	Mon Jun	166	S	1	D	0.00001	M	Molar	4	17	Colon C	KM12	103.04
830932	2106OS4	Mon Jun	166	S	1	D	0.00001	M	Molar	4	9	Colon C	SW-620	95.9743
830932	2106OS4	Mon Jun	166	S	1	D	0.00001	M	Molar	12	14	CNS Car	SF-268	112.807
830932	2106OS4	Mon Jun	166	S	1	D	0.00001	M	Molar	12	15	CNS Car	SF-295	106.743
830932	2106OS4	Mon Jun	166	S	1	D	0.00001	M	Molar	12	16	CNS Car	SF-539	101.437
830932	2106OS4	Mon Jun	166	S	1	D	0.00001	M	Molar	12	2	CNS Car	SNB-19	88.5105
830932	2106OS4	Mon Jun	166	S	1	D	0.00001	M	Molar	12	5	CNS Car	SNB-75	104.44
830932	2106OS4	Mon Jun	166	S	1	D	0.00001	M	Molar	12	9	CNS Car	U251	93.407
830932	2106OS4	Mon Jun	166	S	1	D	0.00001	M	Molar	10	1	Melanon	LOX IMV	95.5346
830932	2106OS4	Mon Jun	166	S	1	D	0.00001	M	Molar	10	2	Melanon	MALME	88.262
830932	2106OS4	Mon Jun	166	S	1	D	0.00001	M	Molar	10	14	Melanon	M14	97.6074
830932	2106OS4	Mon Jun	166	S	1	D	0.00001	M	Molar	5	11	Melanon	MDA-MI	102.206
830932	2106OS4	Mon Jun	166	S	1	D	0.00001	M	Molar	10	5	Melanon	SK-MEL-	102.787
830932	2106OS4	Mon Jun	166	S	1	D	0.00001	M	Molar	10	8	Melanon	SK-MEL-	103.191
830932	2106OS4	Mon Jun	166	S	1	D	0.00001	M	Molar	10	7	Melanon	SK-MEL-	98.726
830932	2106OS4	Mon Jun	166	S	1	D	0.00001	M	Molar	10	21	Melanon	UACC-2	103.645
830932	2106OS4	Mon Jun	166	S	1	D	0.00001	M	Molar	10	20	Melanon	UACC-6	80.9626
830932	2106OS4	Mon Jun	166	S	1	D	0.00001	M	Molar	6	10	Ovarian (	IGROV1	96.9201
830932	2106OS4	Mon Jun	166	S	1	D	0.00001	M	Molar	6	2	Ovarian (	OVCAR-	100.796
830932	2106OS4	Mon Jun	166	S	1	D	0.00001	M	Molar	6	3	Ovarian (	OVCAR-	95.784
830932	2106OS4	Mon Jun	166	S	1	D	0.00001	M	Molar	6	5	Ovarian (	OVCAR-	98.1929
830932	2106OS4	Mon Jun	166	S	1	D	0.00001	M	Molar	5	2	Ovarian (	NCI/ADF	95.8905
830932	2106OS4	Mon Jun	166	S	1	D	0.00001	M	Molar	6	11	Ovarian (	SK-OV-3	97.2551
830932	2106OS4	Mon Jun	166	S	1	D	0.00001	M	Molar	9	18	Renal Ca	786-0	102.062
830932	2106OS4	Mon Jun	166	S	1	D	0.00001	M	Molar	9	13	Renal Ca	A498	101.26
830932	2106OS4	Mon Jun	166	S	1	D	0.00001	M	Molar	9	23	Renal Ca	ACHN	101.278
830932	2106OS4	Mon Jun	166	S	1	D	0.00001	M	Molar	9	15	Renal Ca	CAKI-1	86.4643
830932	2106OS4	Mon Jun	166	S	1	D	0.00001	M	Molar	9	16	Renal Ca	RXF 393	86.1479
830932	2106OS4	Mon Jun	166	S	1	D	0.00001	M	Molar	9	8	Renal Ca	SN12C	97.1243
830932	2106OS4	Mon Jun	166	S	1	D	0.00001	M	Molar	9	24	Renal Ca	TK-10	123.097
830932	2106OS4	Mon Jun	166	S	1	D	0.00001	M	Molar	9	4	Renal Ca	UO-31	75.3746
830932	2106OS4	Mon Jun	166	S	1	D	0.00001	M	Molar	11	1	Prostate	PC-3	84.9163
830932	2106OS4	Mon Jun	166	S	1	D	0.00001	M	Molar	11	3	Prostate	DU-145	113.628
830932	2106OS4	Mon Jun	166	S	1	D	0.00001	M	Molar	5	1	Breast C	MCF7	75.0937
830932	2106OS4	Mon Jun	166	S	1	D	0.00001	M	Molar	5	5	Breast C	MDA-MI	94.7989
830932	2106OS4	Mon Jun	166	S	1	D	0.00001	M	Molar	5	6	Breast C	HS 578T	91.0116
830932	2106OS4	Mon Jun	166	S	1	D	0.00001	M	Molar	5	13	Breast C	BT-549	125.507
830932	2106OS4	Mon Jun	166	S	1	D	0.00001	M	Molar	5	14	Breast C	T-47D	89.0178
830932	2106OS4	Mon Jun	166	S	1	D	0.00001	M	Molar	5	18	Breast C	MDA-MI	90.3603

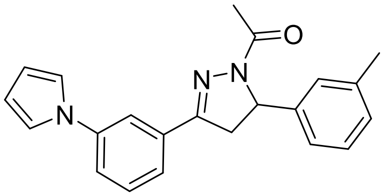

O
N
N
N

7c

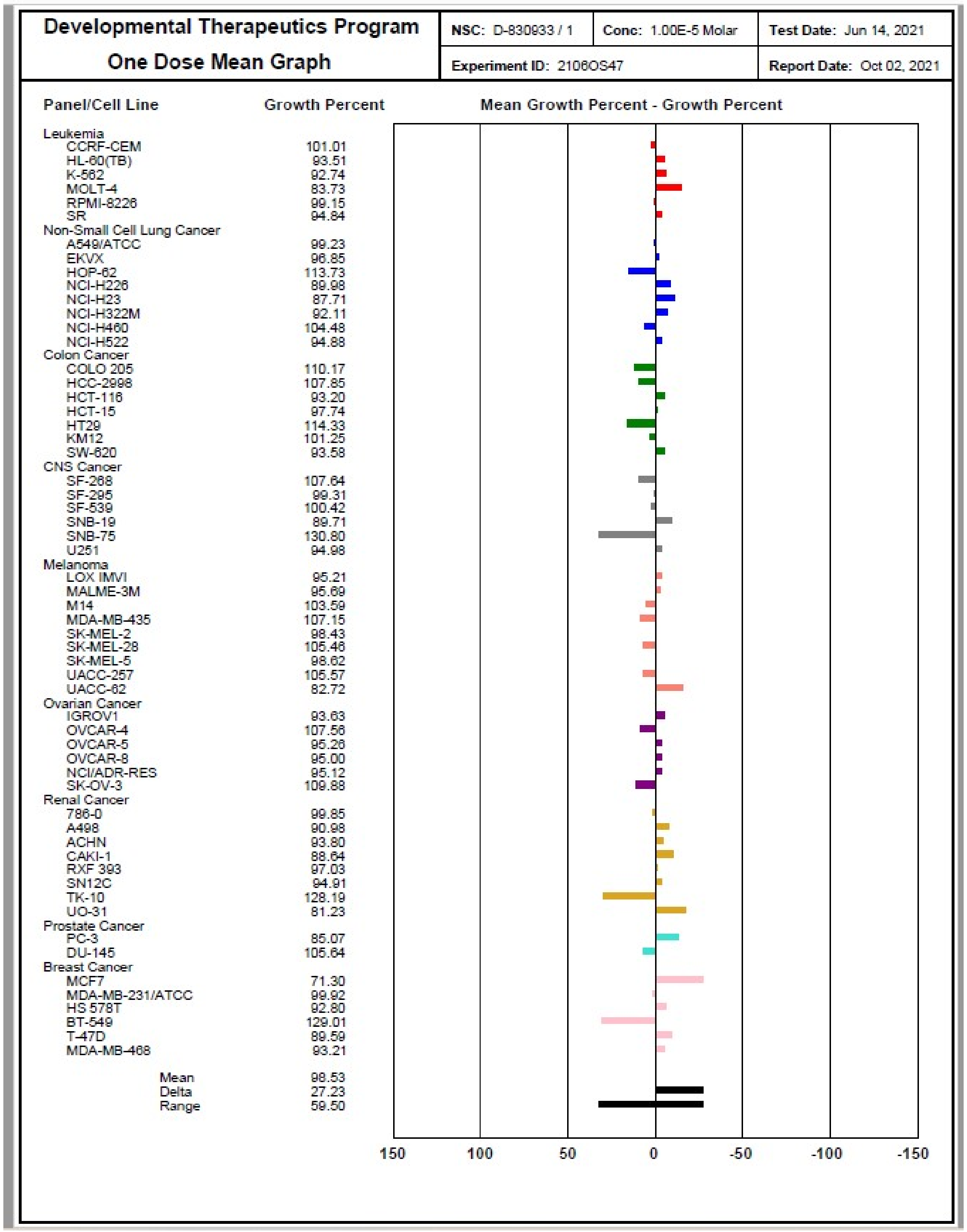

Developmental Therapeutics Program
One Dose Mean Graph
NSC: D-830933 / 1
Conc: 1.00E-5 Molar
Test Date: Jun 14, 2021
Experiment ID: 2106OS47
Report Date: Oct 02, 2021
Panel/Cell Line
Growth Percent
Mean Growth Percent - Growth Percent
Leukemia
CCRF-CEM 101.01
HL-60(TB) 93.51
K-562 92.74
MOLT-4 83.73
RPMI-8226 99.15
SR 94.84
Non-Small Cell Lung Cancer
A549/ATCC 99.23
EKVX 96.85
HOP-62 113.73
NCI-H226 89.98
NCI-H23 87.71
NCI-H322M 92.11
NCI-H460 104.48
NCI-H522 94.88
Colon Cancer
COLO 205 110.17
HCC-2998 107.85
HCT-116 93.20
HCT-15 97.74
HT29 114.33
KM12 101.25
SW-620 93.58
CNS Cancer
SF-268 107.64
SF-295 99.31
SF-539 100.42
SNB-19 89.71
SNB-75 130.80
U251 94.98
Melanoma
LOX IMVI 95.21
MALME-3M 95.69
M14 103.59
MDA-MB-435 107.15
SK-MEL-2 98.43
SK-MEL-28 105.46
SK-MEL-5 98.62
UACC-257 105.57
UACC-62 82.72
Ovarian Cancer
IGROV1 93.63
OVCAR-4 107.56
OVCAR-5 95.26
OVCAR-8 95.00
NCI/ADR-RES 95.12
SK-OV-3 109.88
Renal Cancer
786-0 99.85
A498 90.98
ACHN 93.80
CAKI-1 88.64
RXF 393 97.03
SN12C 94.91
TK-10 128.19
UO-31 81.23
Prostate Cancer
PC-3 85.07
DU-145 105.64
Breast Cancer
MCF7 71.30
MDA-MB-231/ATCC 99.92
HS 578T 92.80
BT-549 129.01
T-47D 89.59
MDA-MB-468 93.21
Mean 98.53
Delta 27.23
Range 59.50
150 100 50 0 -50 -100 -150

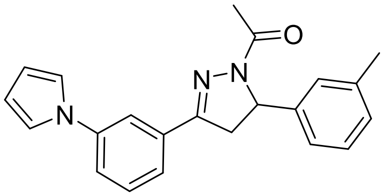

7c

A1 | NSC

	A	B	C	D	E	F	G	H	I	J	K	L	M	N	O
1	NSC	EXPID	PLANDA	TESTSE	PREFIX	SAMPLE	DISCREI	CONC	CONCUI	CONCUI	PANELN	CELLNB	PANELN	CELLNA	GIPRCNT
2	830933	2106OS4	Mon Jun	176	S	1	D	0.00001	M	Molar	7	3	Leukemi	CCRF-C	101.014
3	830933	2106OS4	Mon Jun	176	S	1	D	0.00001	M	Molar	7	8	Leukemi	HL-60(TE	93.508
4	830933	2106OS4	Mon Jun	176	S	1	D	0.00001	M	Molar	7	5	Leukemi	K-562	92.736
5	830933	2106OS4	Mon Jun	176	S	1	D	0.00001	M	Molar	7	6	Leukemi	MOLT-4	83.7307
6	830933	2106OS4	Mon Jun	176	S	1	D	0.00001	M	Molar	7	10	Leukemi	RPMI-82	99.152
7	830933	2106OS4	Mon Jun	176	S	1	D	0.00001	M	Molar	7	19	Leukemi	SR	94.8418
8	830933	2106OS4	Mon Jun	176	S	1	D	0.00001	M	Molar	1	4	Non-Sm	A549/AT	99.2272
9	830933	2106OS4	Mon Jun	176	S	1	D	0.00001	M	Molar	1	8	Non-Sm	EKVX	96.8486
10	830933	2106OS4	Mon Jun	176	S	1	D	0.00001	M	Molar	1	26	Non-Sm	HOP-62	113.735
11	830933	2106OS4	Mon Jun	176	S	1	D	0.00001	M	Molar	1	13	Non-Sm	NCI-H22	89.9803
12	830933	2106OS4	Mon Jun	176	S	1	D	0.00001	M	Molar	1	1	Non-Sm	NCI-H23	87.7086
13	830933	2106OS4	Mon Jun	176	S	1	D	0.00001	M	Molar	1	17	Non-Sm	NCI-H32	92.1063
14	830933	2106OS4	Mon Jun	176	S	1	D	0.00001	M	Molar	1	21	Non-Sm	NCI-H46	104.481
15	830933	2106OS4	Mon Jun	176	S	1	D	0.00001	M	Molar	1	3	Non-Sm	NCI-H52	94.88
16	830933	2106OS4	Mon Jun	176	S	1	D	0.00001	M	Molar	4	10	Colon C	COLO 20	110.165
17	830933	2106OS4	Mon Jun	176	S	1	D	0.00001	M	Molar	4	2	Colon C	HCC-29	107.85
18	830933	2106OS4	Mon Jun	176	S	1	D	0.00001	M	Molar	4	3	Colon C	HCT-116	93.2047
19	830933	2106OS4	Mon Jun	176	S	1	D	0.00001	M	Molar	4	15	Colon C	HCT-15	97.7396
20	830933	2106OS4	Mon Jun	176	S	1	D	0.00001	M	Molar	4	1	Colon C	HT29	114.332
21	830933	2106OS4	Mon Jun	176	S	1	D	0.00001	M	Molar	4	17	Colon C	KM12	101.246
22	830933	2106OS4	Mon Jun	176	S	1	D	0.00001	M	Molar	4	9	Colon C	SW-620	93.5781
23	830933	2106OS4	Mon Jun	176	S	1	D	0.00001	M	Molar	12	14	CNS Car	SF-268	107.638
24	830933	2106OS4	Mon Jun	176	S	1	D	0.00001	M	Molar	12	15	CNS Car	SF-295	99.3118
25	830933	2106OS4	Mon Jun	176	S	1	D	0.00001	M	Molar	12	16	CNS Car	SF-539	100.421
26	830933	2106OS4	Mon Jun	176	S	1	D	0.00001	M	Molar	12	2	CNS Car	SNB-19	89.7084
27	830933	2106OS4	Mon Jun	176	S	1	D	0.00001	M	Molar	12	5	CNS Car	SNB-75	130.805
28	830933	2106OS4	Mon Jun	176	S	1	D	0.00001	M	Molar	12	9	CNS Car	U251	94.9809
29	830933	2106OS4	Mon Jun	176	S	1	D	0.00001	M	Molar	10	1	Melanon	LOX IMV	95.2119
30	830933	2106OS4	Mon Jun	176	S	1	D	0.00001	M	Molar	10	2	Melanon	MALME	95.688
31	830933	2106OS4	Mon Jun	176	S	1	D	0.00001	M	Molar	10	14	Melanon	M14	103.591
32	830933	2106OS4	Mon Jun	176	S	1	D	0.00001	M	Molar	5	11	Melanon	MDA-MI	107.152
33	830933	2106OS4	Mon Jun	176	S	1	D	0.00001	M	Molar	10	5	Melanon	SK-MEL-	98.4263
34	830933	2106OS4	Mon Jun	176	S	1	D	0.00001	M	Molar	10	8	Melanon	SK-MEL-	105.462
35	830933	2106OS4	Mon Jun	176	S	1	D	0.00001	M	Molar	10	7	Melanon	SK-MEL-	98.6203
36	830933	2106OS4	Mon Jun	176	S	1	D	0.00001	M	Molar	10	21	Melanon	UACC-2	105.57
37	830933	2106OS4	Mon Jun	176	S	1	D	0.00001	M	Molar	10	20	Melanon	UACC-6	82.7174
38	830933	2106OS4	Mon Jun	176	S	1	D	0.00001	M	Molar	6	10	Ovarian (	IGROV1	93.6344
39	830933	2106OS4	Mon Jun	176	S	1	D	0.00001	M	Molar	6	2	Ovarian (	OVCAR-	107.556
40	830933	2106OS4	Mon Jun	176	S	1	D	0.00001	M	Molar	6	3	Ovarian (	OVCAR-	95.2616
41	830933	2106OS4	Mon Jun	176	S	1	D	0.00001	M	Molar	6	5	Ovarian (	OVCAR-	95.0001
42	830933	2106OS4	Mon Jun	176	S	1	D	0.00001	M	Molar	5	2	Ovarian (	NCI/ADF	95.1208
43	830933	2106OS4	Mon Jun	176	S	1	D	0.00001	M	Molar	6	11	Ovarian (	SK-OV-3	109.88
44	830933	2106OS4	Mon Jun	176	S	1	D	0.00001	M	Molar	9	18	Renal Ca	786-0	99.8529
45	830933	2106OS4	Mon Jun	176	S	1	D	0.00001	M	Molar	9	13	Renal Ca	A498	90.9825
46	830933	2106OS4	Mon Jun	176	S	1	D	0.00001	M	Molar	9	23	Renal Ca	ACHN	93.799
47	830933	2106OS4	Mon Jun	176	S	1	D	0.00001	M	Molar	9	15	Renal Ca	CAKI-1	88.6422
48	830933	2106OS4	Mon Jun	176	S	1	D	0.00001	M	Molar	9	16	Renal Ca	RXF 393	97.0323
49	830933	2106OS4	Mon Jun	176	S	1	D	0.00001	M	Molar	9	8	Renal Ca	SN12C	94.9147
50	830933	2106OS4	Mon Jun	176	S	1	D	0.00001	M	Molar	9	24	Renal Ca	TK-10	128.185
51	830933	2106OS4	Mon Jun	176	S	1	D	0.00001	M	Molar	9	4	Renal Ca	UO-31	81.2316
52	830933	2106OS4	Mon Jun	176	S	1	D	0.00001	M	Molar	11	1	Prostate	PC-3	85.0688
53	830933	2106OS4	Mon Jun	176	S	1	D	0.00001	M	Molar	11	3	Prostate	DU-145	105.639
54	830933	2106OS4	Mon Jun	176	S	1	D	0.00001	M	Molar	5	1	Breast C	MCF7	71.299
55	830933	2106OS4	Mon Jun	176	S	1	D	0.00001	M	Molar	5	5	Breast C	MDA-MI	99.9207
56	830933	2106OS4	Mon Jun	176	S	1	D	0.00001	M	Molar	5	6	Breast C	HS 578T	92.7988
57	830933	2106OS4	Mon Jun	176	S	1	D	0.00001	M	Molar	5	13	Breast C	BT-549	129.005
58	830933	2106OS4	Mon Jun	176	S	1	D	0.00001	M	Molar	5	14	Breast C	T-47D	89.594
59	830933	2106OS4	Mon Jun	176	S	1	D	0.00001	M	Molar	5	18	Breast C	MDA-MI	93.2117

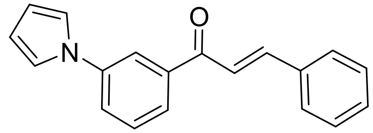
O
N

5a

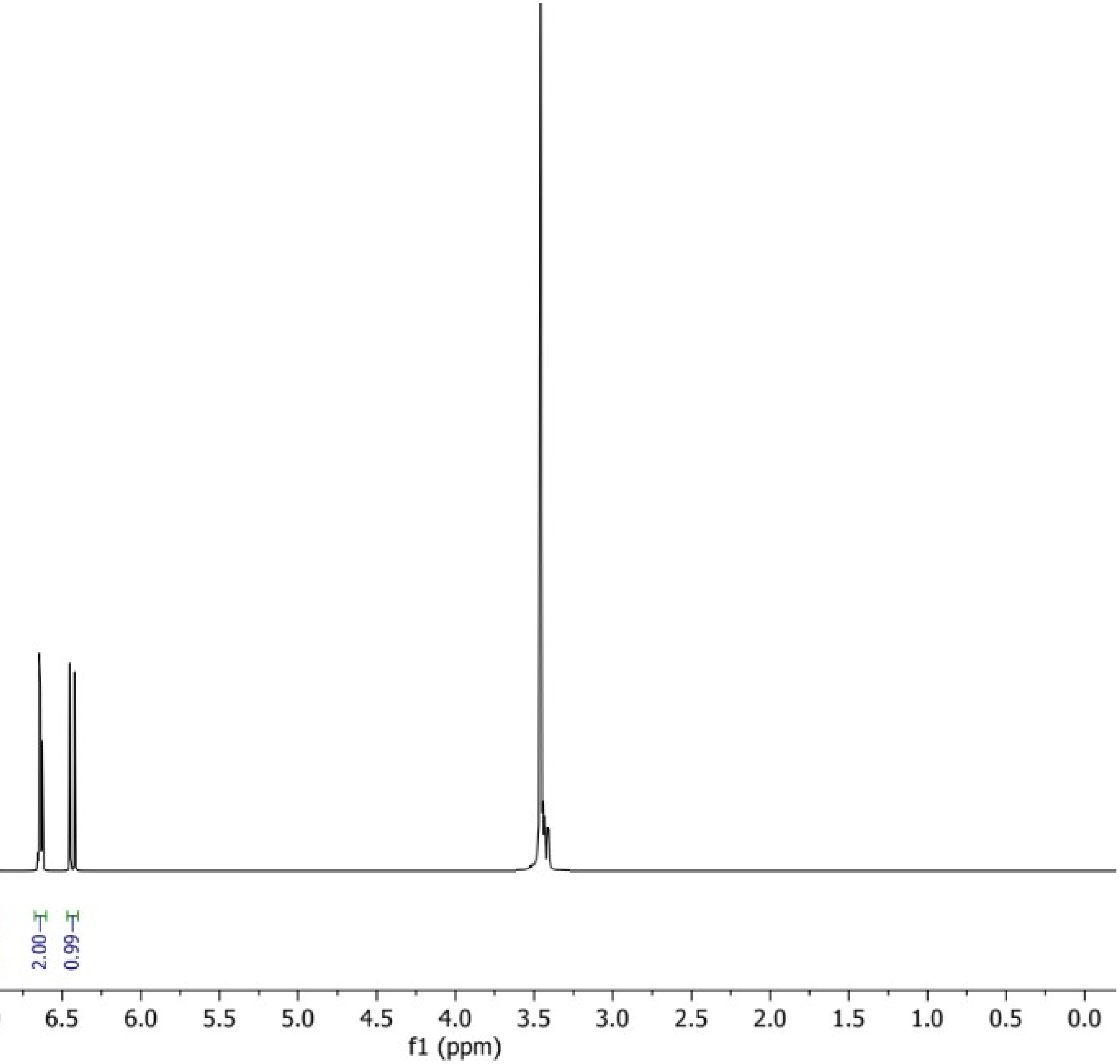
0.99
1.01
2.00
1.63
1.37
1.01
2.01
2.00
2.00
0.99
9.5
9.0
8.5
8.0
7.5
7.0
6.5
6.0
5.5
5.0
4.5
4.0
3.5
3.0
2.5
2.0
1.5
1.0
0.5
0.0
f1 (ppm)

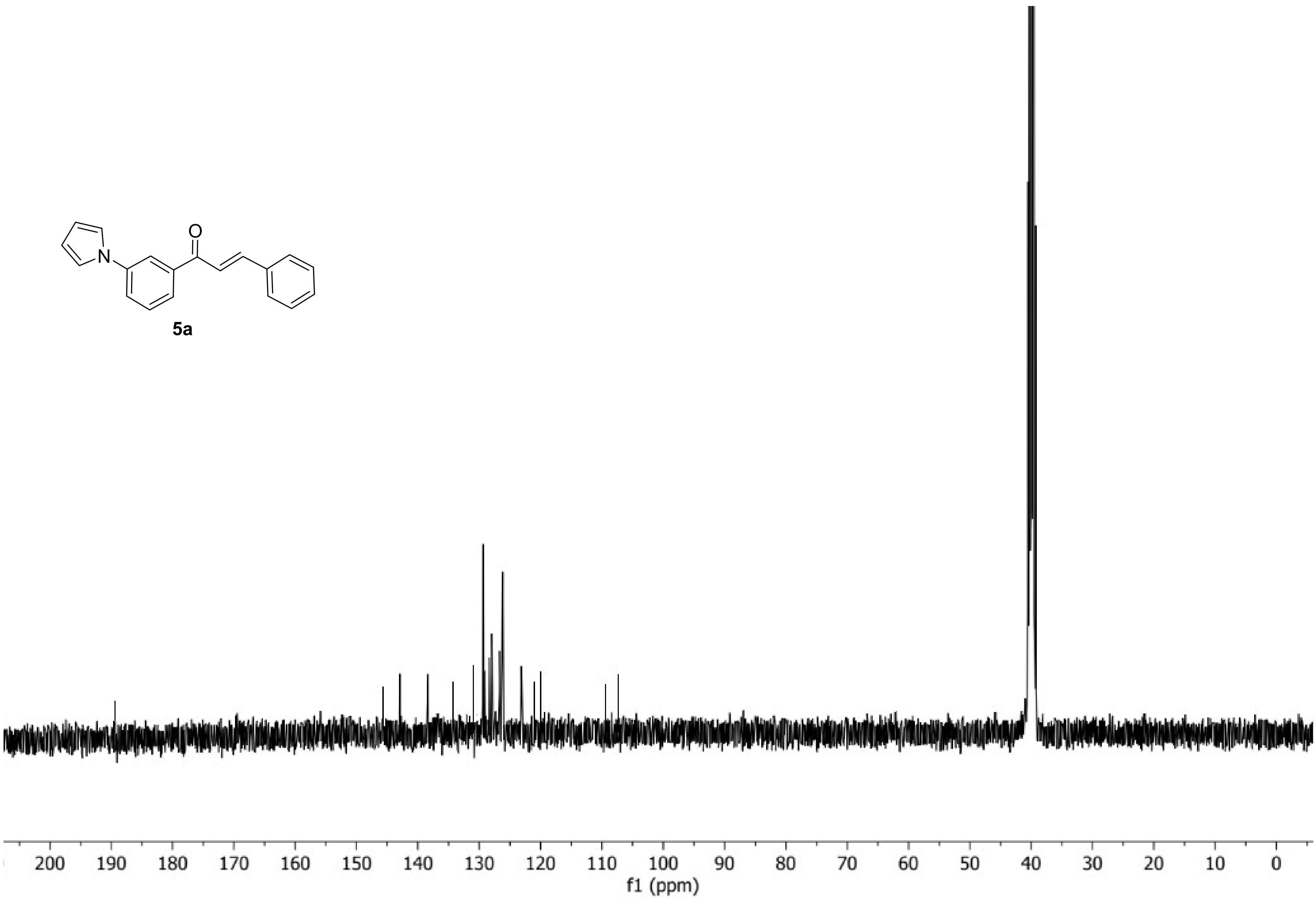

5a
200 190 180 170 160 150 140 130 120 110 100 90 80 70 60 50 40 30 20 10 0
f1 (ppm)

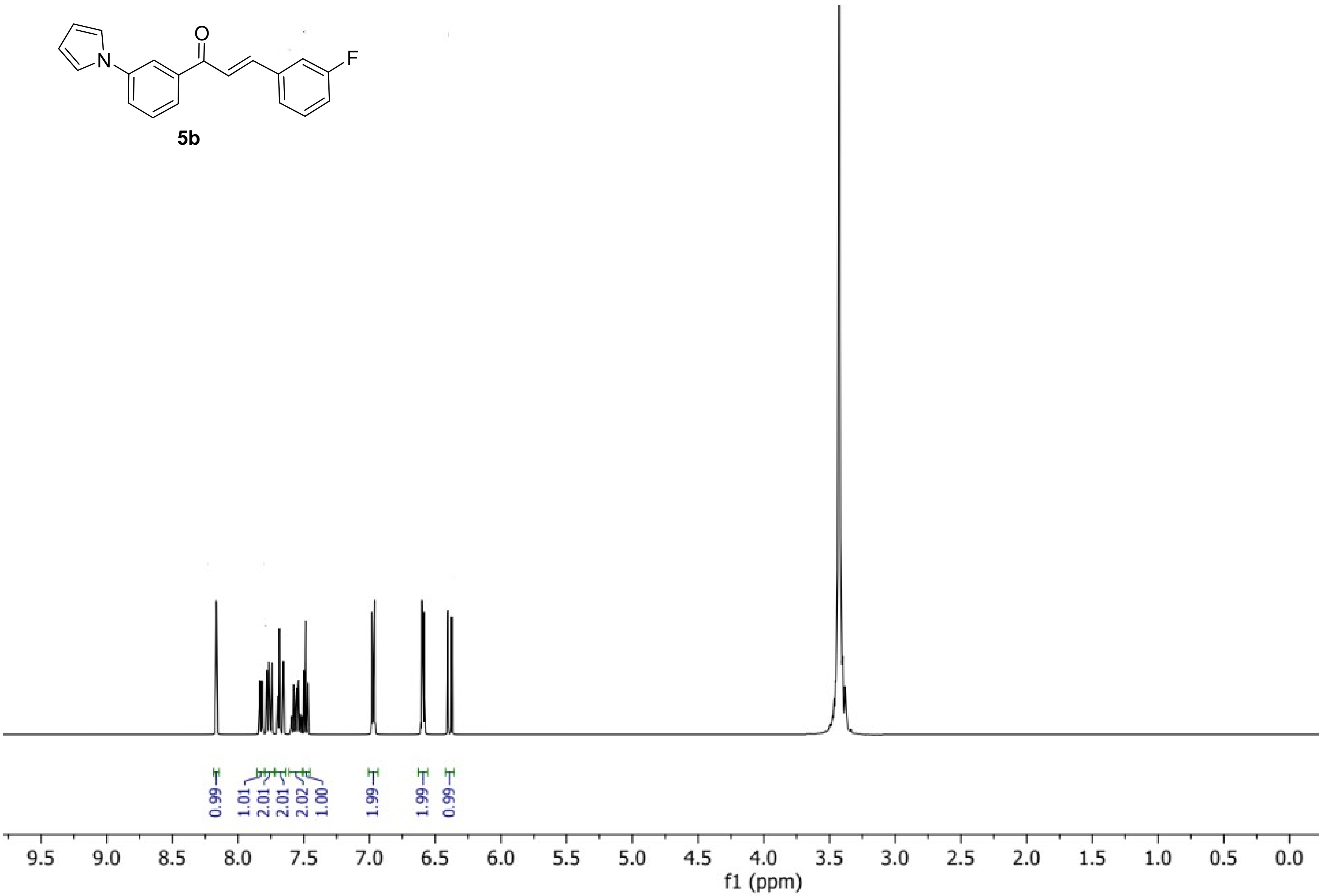
O
N
F
5b
0.99
1.01
2.01
2.01
2.02
1.00
1.99
1.99
0.99
9.5 9.0 8.5 8.0 7.5 7.0 6.5 6.0 5.5 5.0 4.5 4.0 3.5 3.0 2.5 2.0 1.5 1.0 0.5 0.0
f1 (ppm)

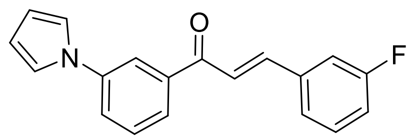

O
N
F

5b

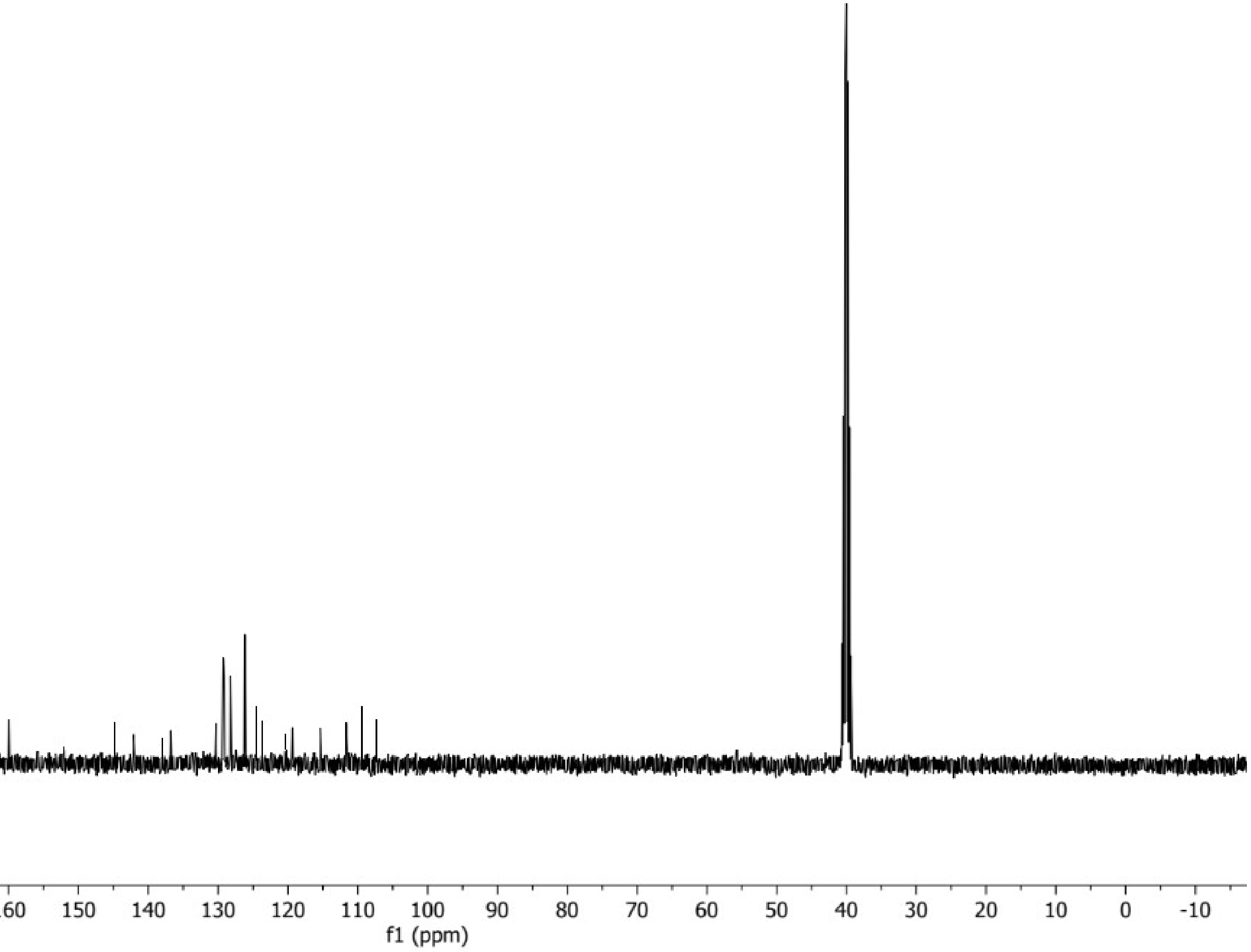

210 200 190 180 170 160 150 140 130 120 110 100 90 80 70 60 50 40 30 20 10 0 -10
f1 (ppm)

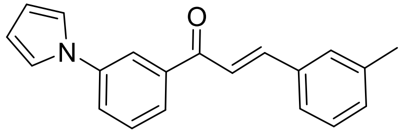

O
N

5c

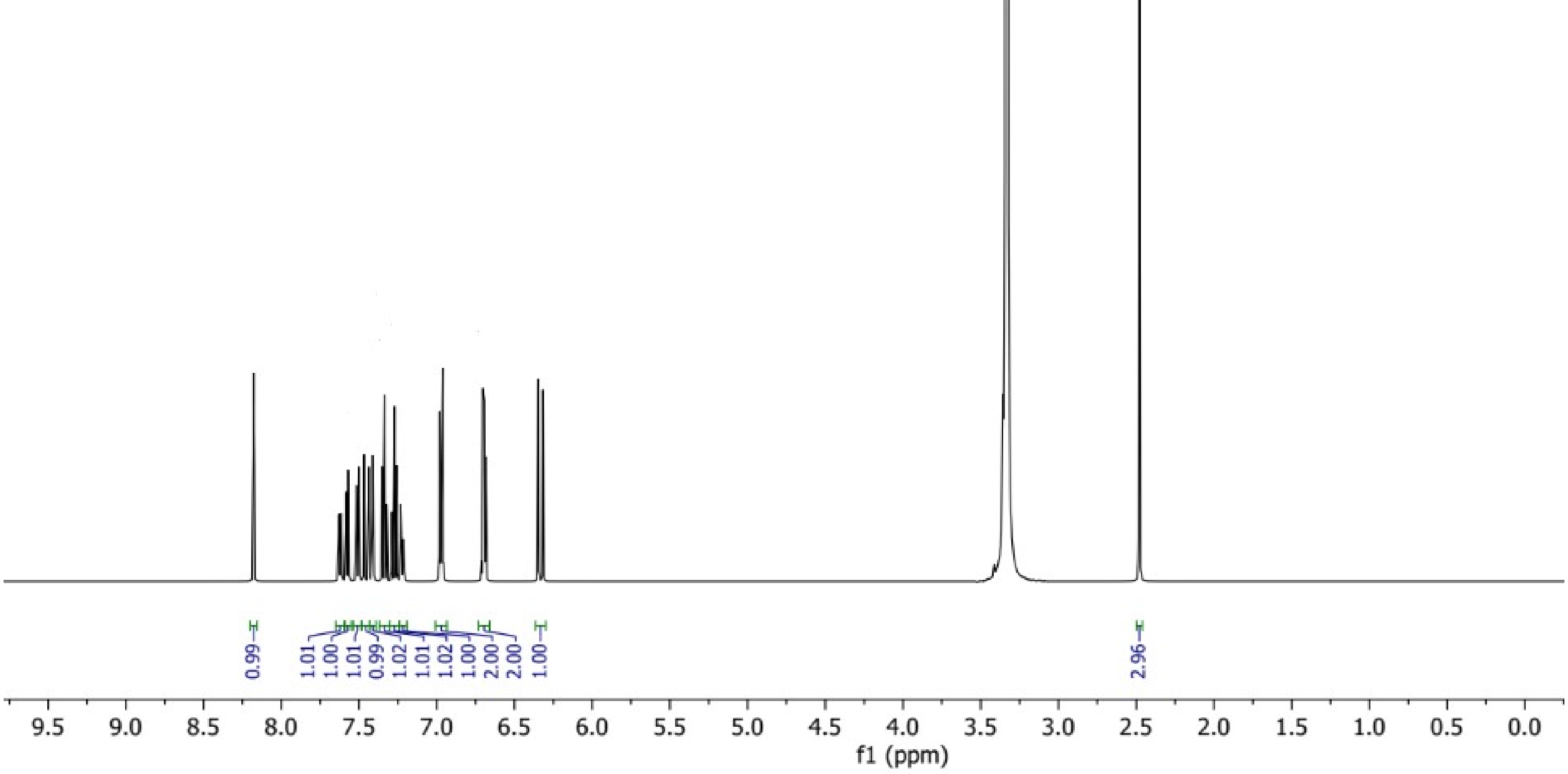

0.99
1.01
1.00
1.01
0.99
1.02
1.01
1.02
1.00
2.00
2.00
1.00
2.96
9.5 9.0 8.5 8.0 7.5 7.0 6.5 6.0 5.5 5.0 4.5 4.0 3.5 3.0 2.5 2.0 1.5 1.0 0.5 0.0
f1 (ppm)

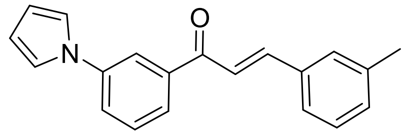

O
N

5c

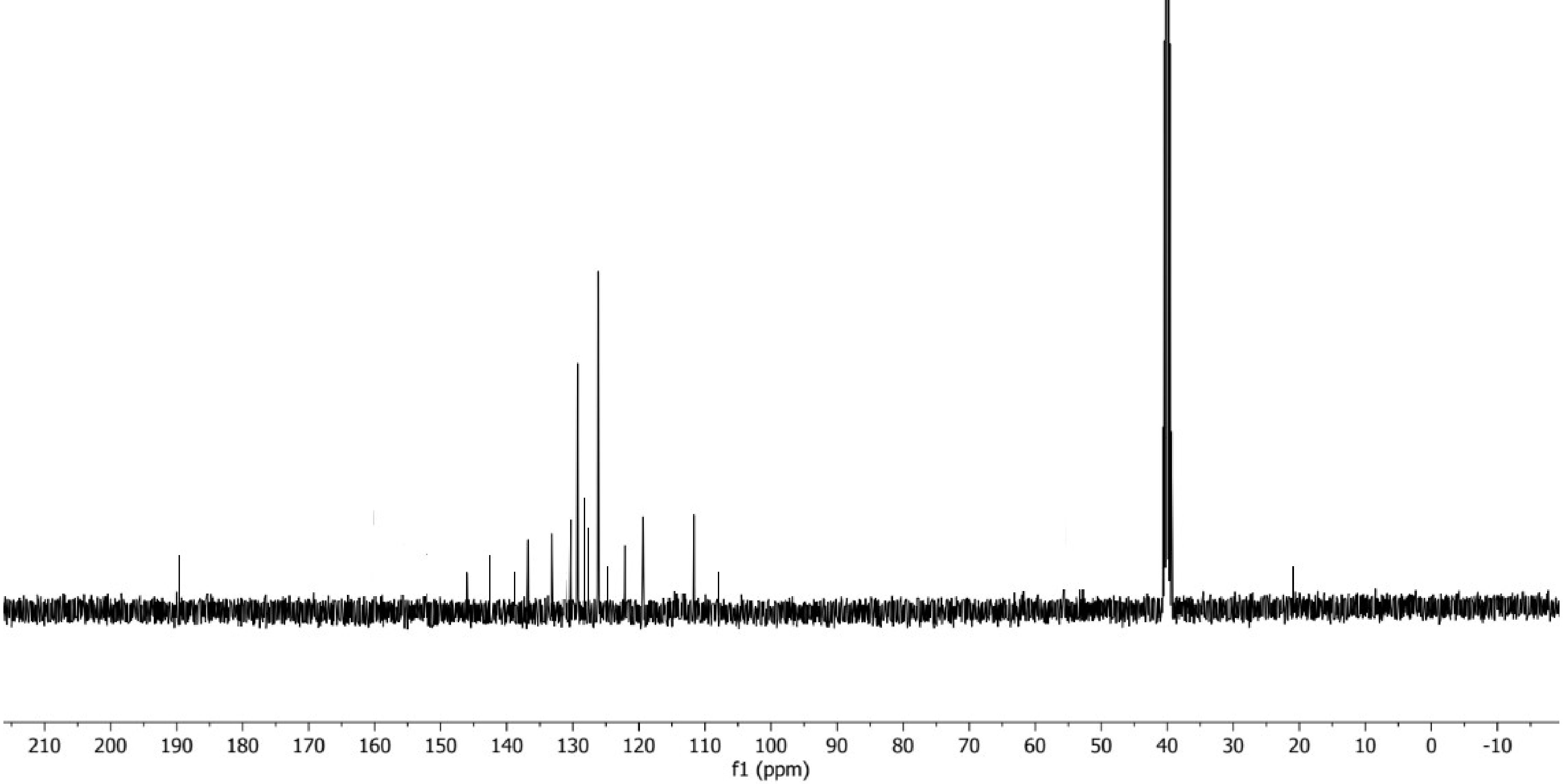

210 200 190 180 170 160 150 140 130 120 110 100 90 80 70 60 50 40 30 20 10 0 -10
f1 (ppm)

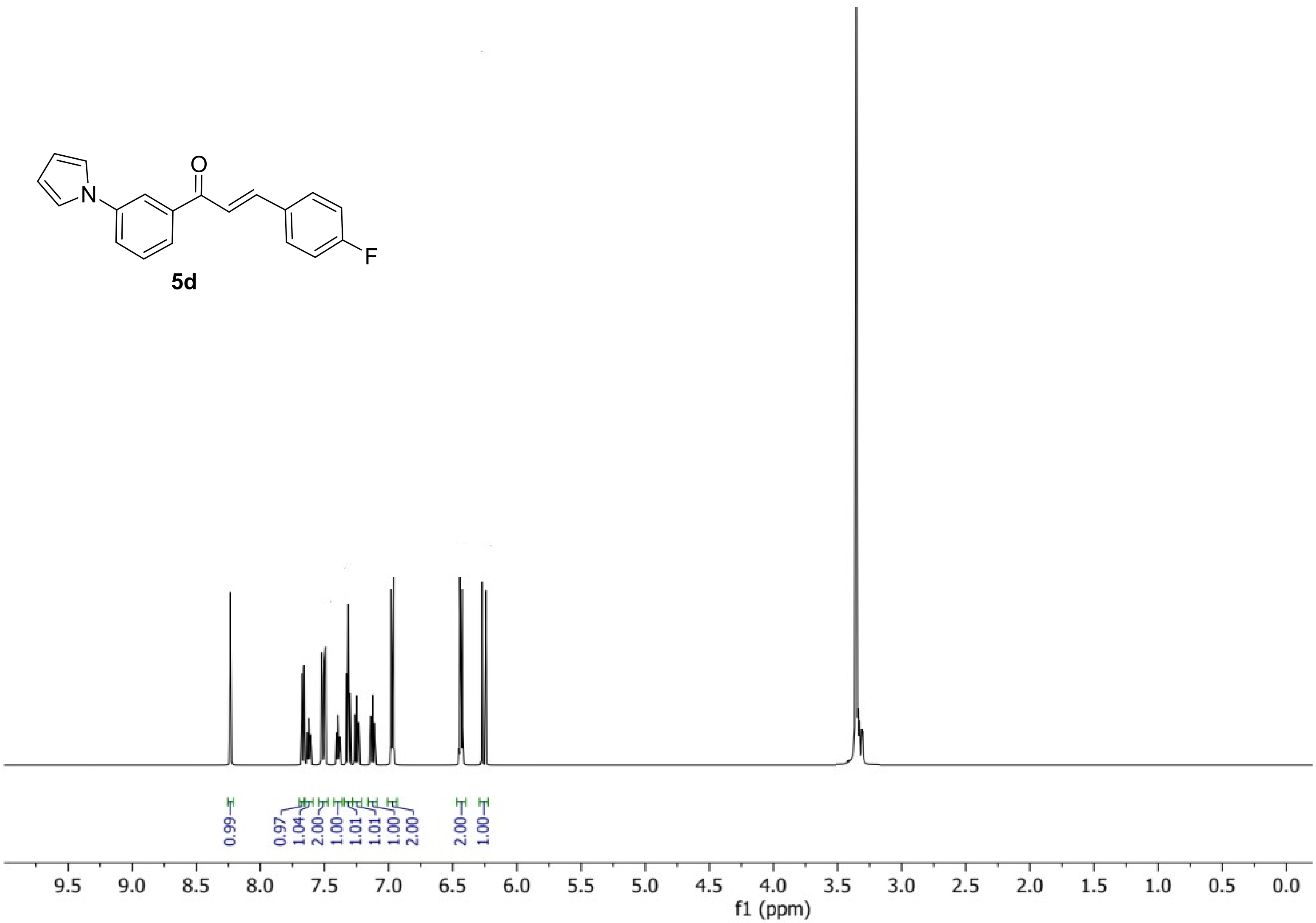
O
N
F
5d
0.99
0.97
1.04
2.00
1.00
1.01
1.01
1.00
2.00
2.00
1.00
9.5 9.0 8.5 8.0 7.5 7.0 6.5 6.0 5.5 5.0 4.5 4.0 3.5 3.0 2.5 2.0 1.5 1.0 0.5 0.0
f1 (ppm)

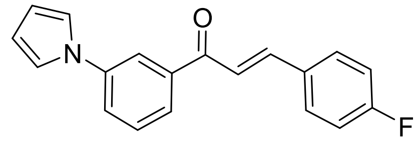
O
N
F

5d

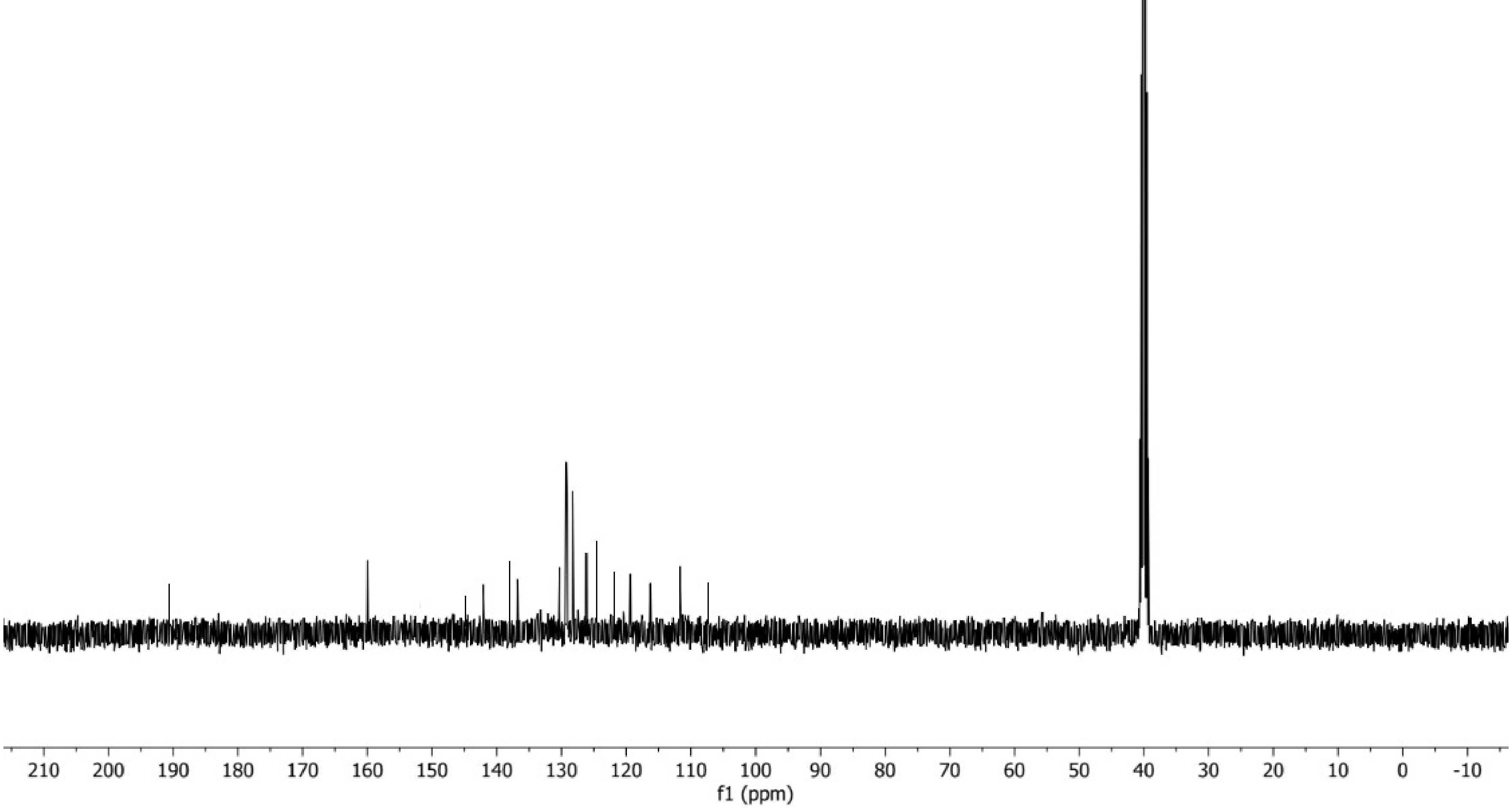
210
200
190
180
170
160
150
140
130
120
110
100
90
80
70
60
50
40
30
20
10
0
-10
f1 (ppm)

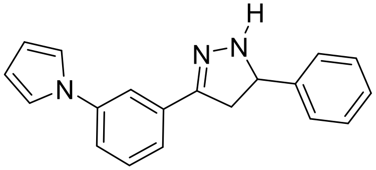
H
N
N
N

6a

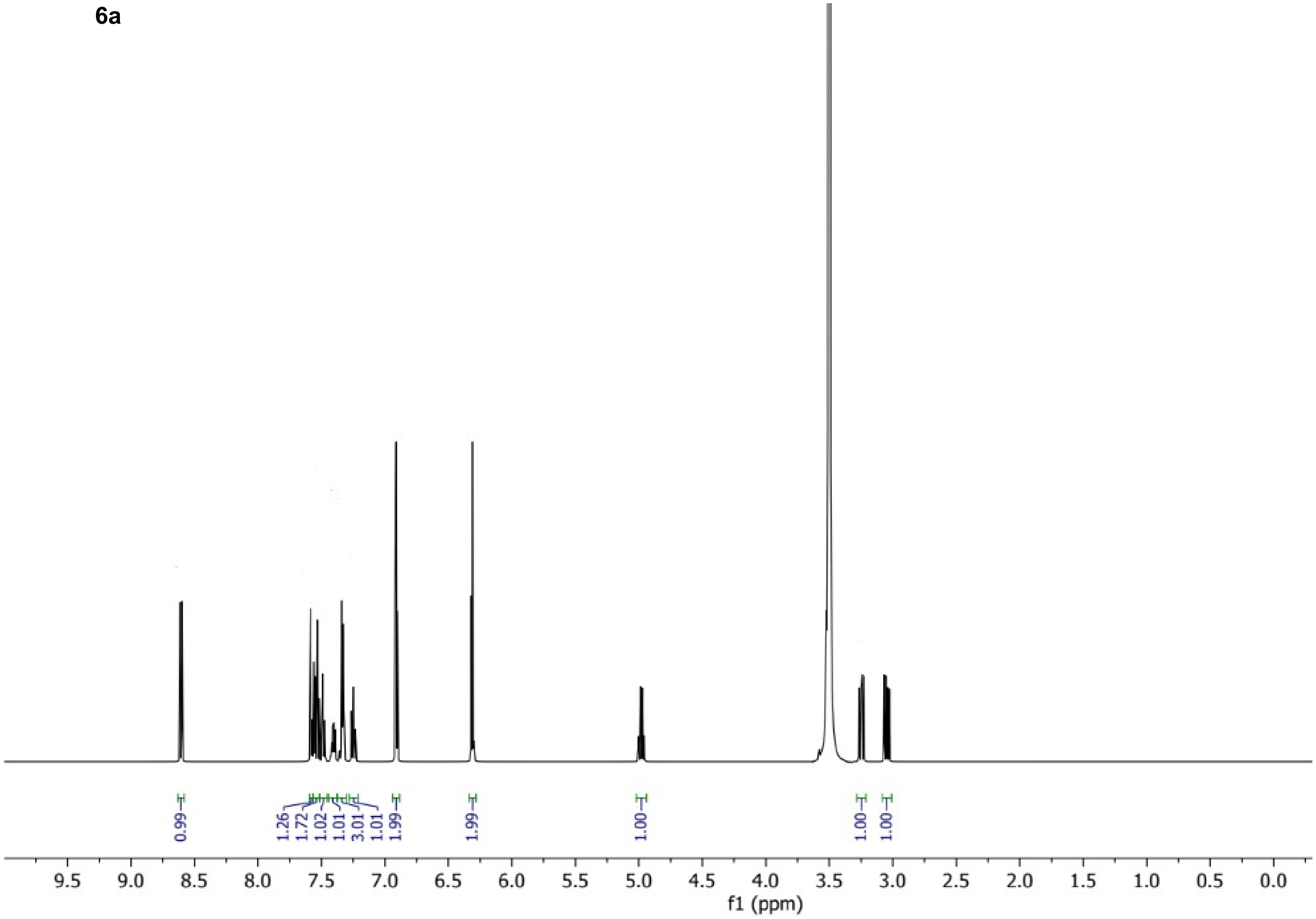
0.99
1.26
1.72
1.02
1.01
3.01
1.01
1.99
1.99
1.00
1.00
1.00
9.5 9.0 8.5 8.0 7.5 7.0 6.5 6.0 5.5 5.0 4.5 4.0 3.5 3.0 2.5 2.0 1.5 1.0 0.5 0.0
f1 (ppm)

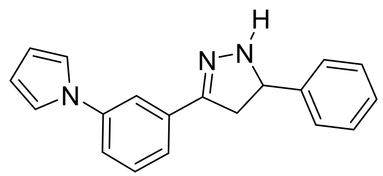
H
N
N
N

6a

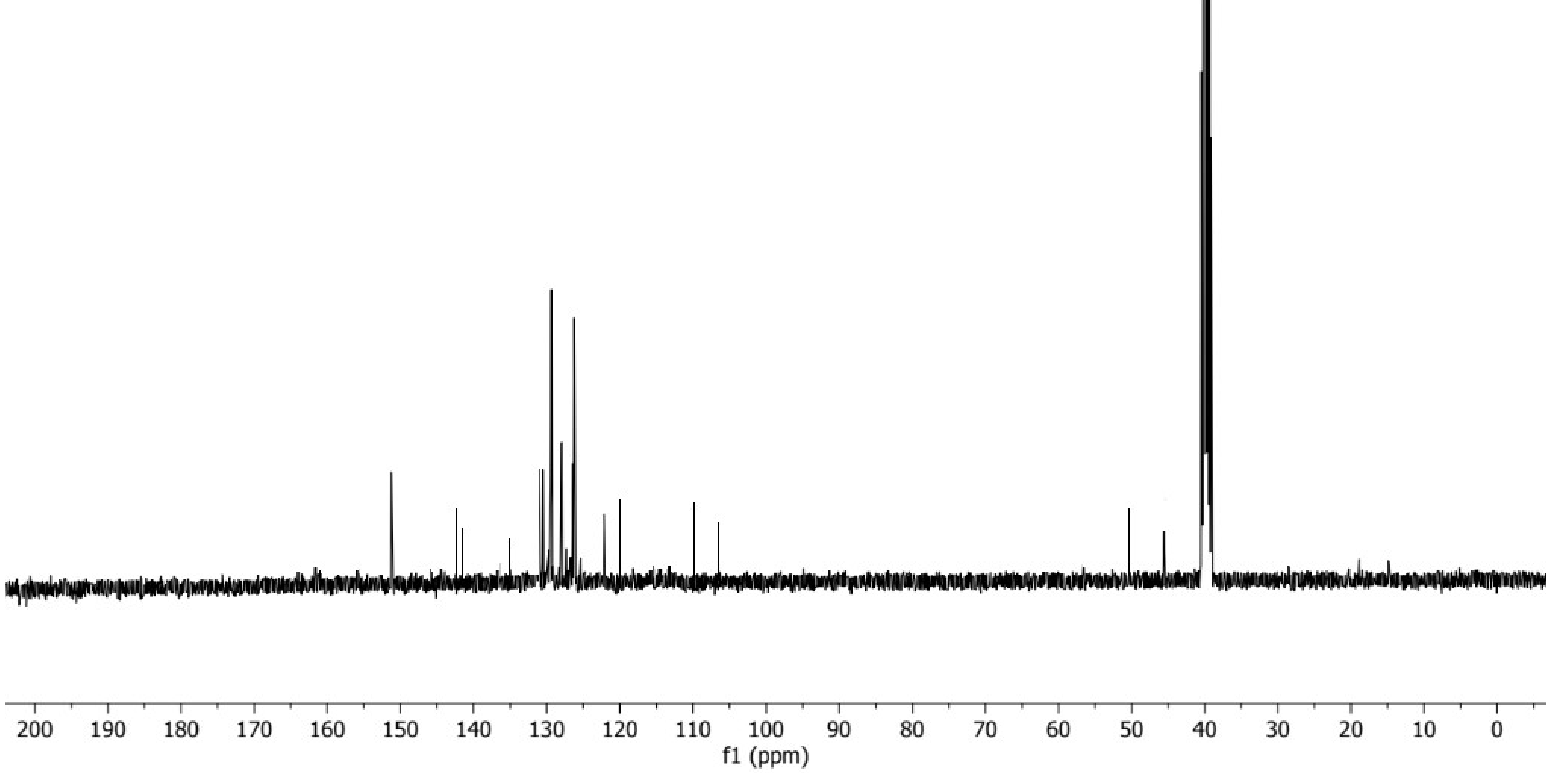
200 190 180 170 160 150 140 130 120 110 100 90 80 70 60 50 40 30 20 10 0
f1 (ppm)

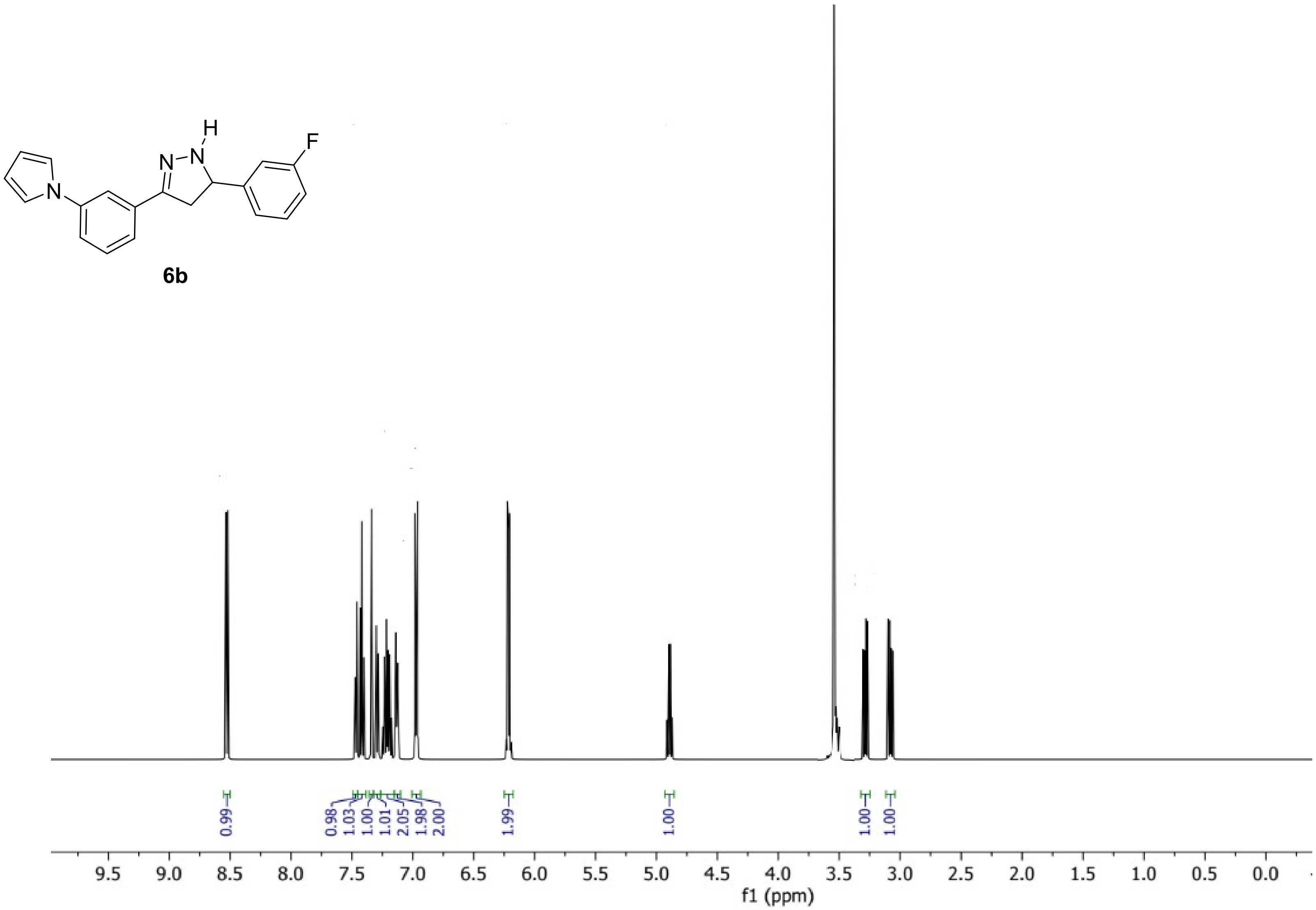
H
N
N
N
F
6b
0.99
0.98
1.03
1.00
1.01
2.05
1.98
2.00
1.99
1.00
1.00
1.00
9.5 9.0 8.5 8.0 7.5 7.0 6.5 6.0 5.5 5.0 4.5 4.0 3.5 3.0 2.5 2.0 1.5 1.0 0.5 0.0
f1 (ppm)

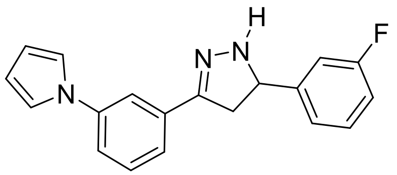

6b

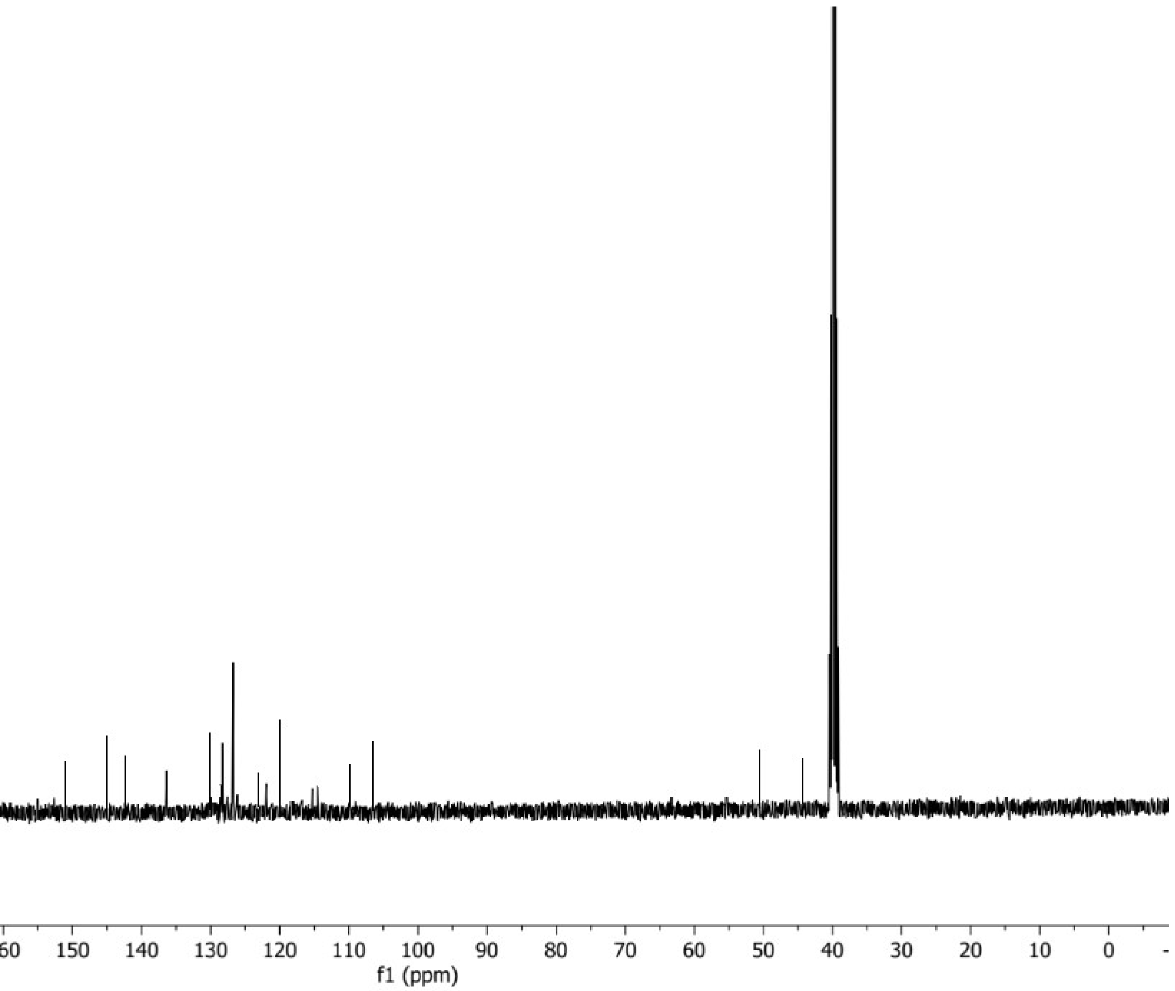

210 200 190 180 170 160 150 140 130 120 110 100 90 80 70 60 50 40 30 20 10 0
f1 (ppm)

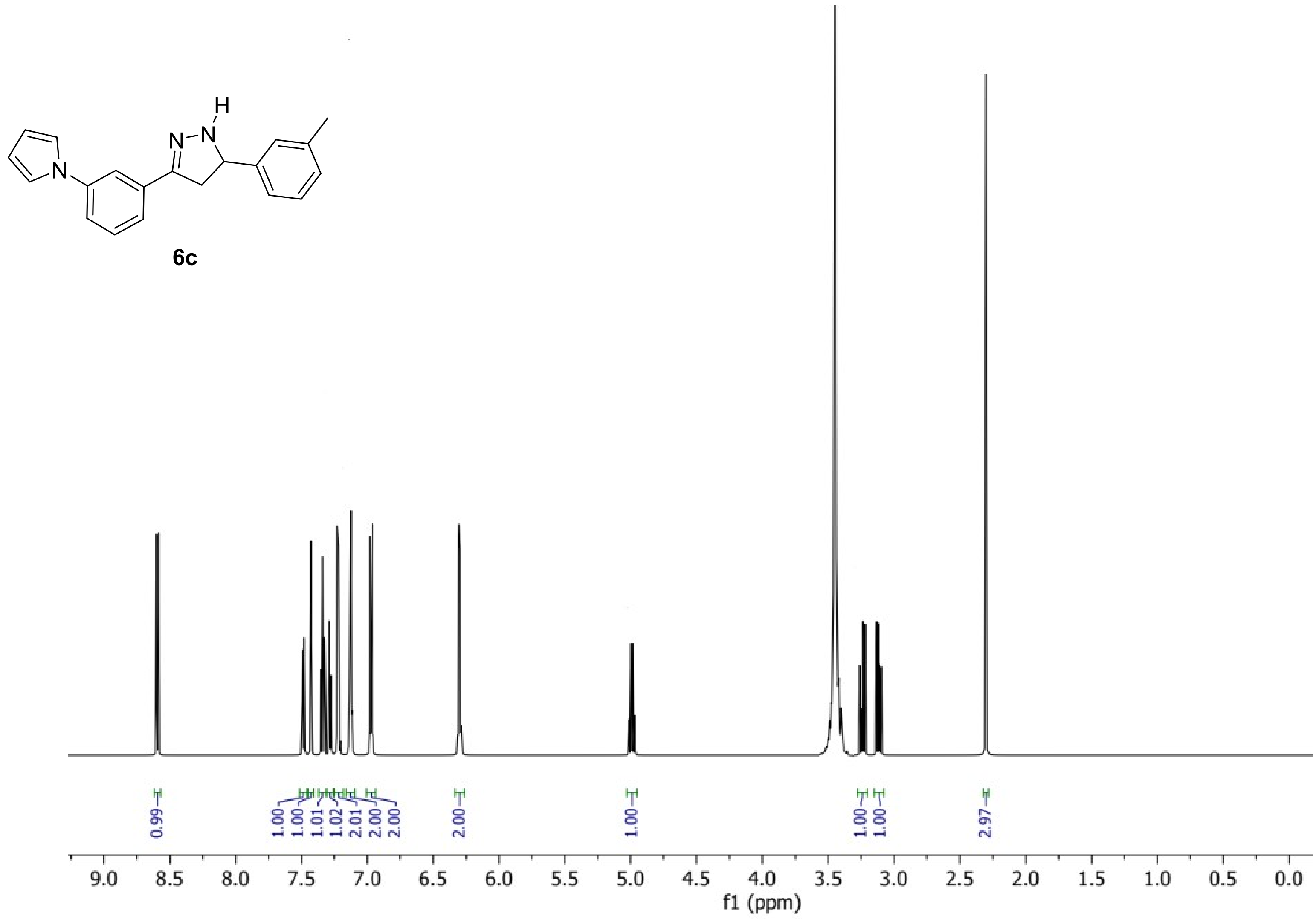
N
N
N
H
0.99
1.00
1.00
1.01
1.02
2.01
2.00
2.00
2.00
1.00
1.00
1.00
2.97
9.0 8.5 8.0 7.5 7.0 6.5 6.0 5.5 5.0 4.5 4.0 3.5 3.0 2.5 2.0 1.5 1.0 0.5 0.0
f1 (ppm)

6c

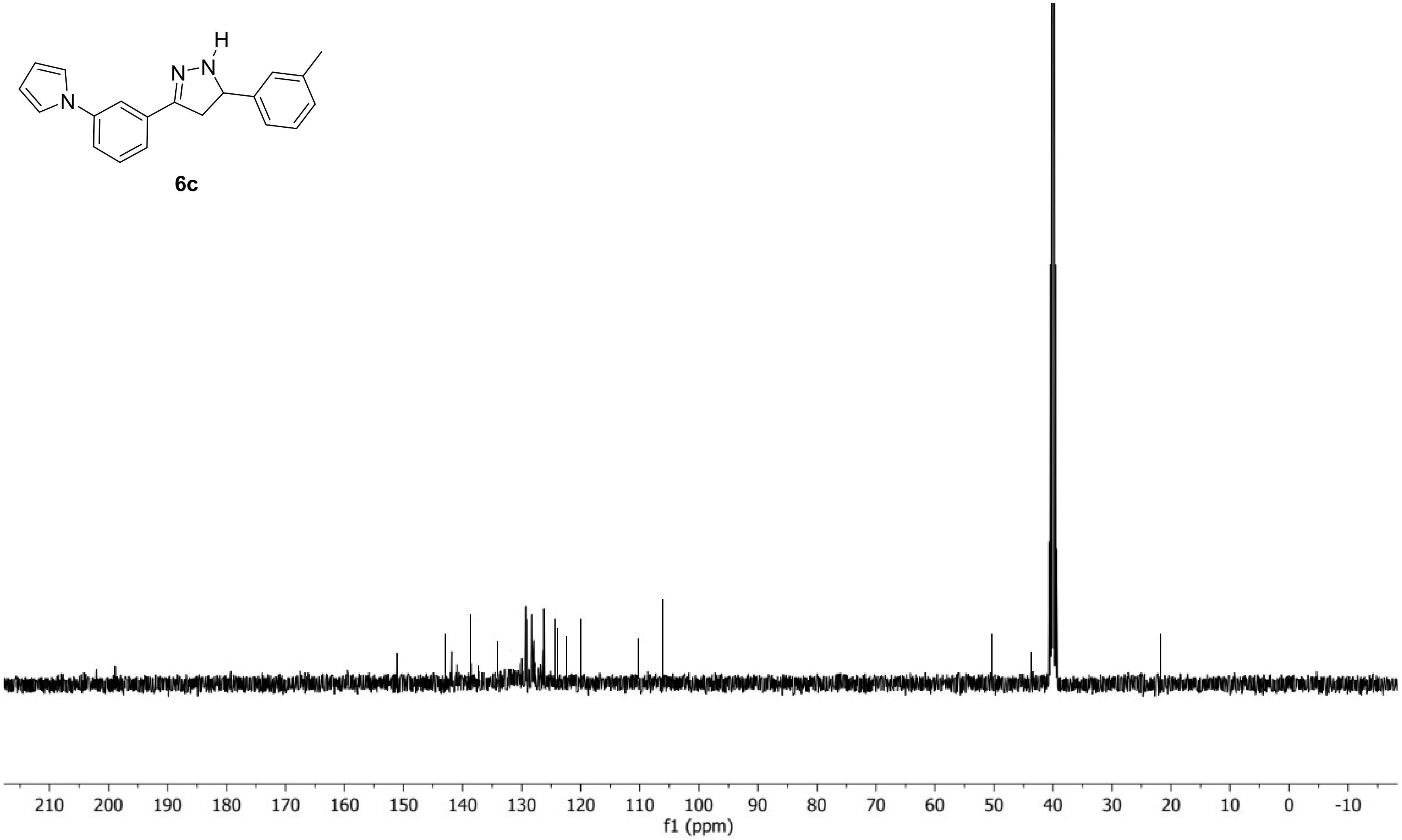
H
N
N
N
210 200 190 180 170 160 150 140 130 120 110 100 90 80 70 60 50 40 30 20 10 0 -10
f1 (ppm)

6c

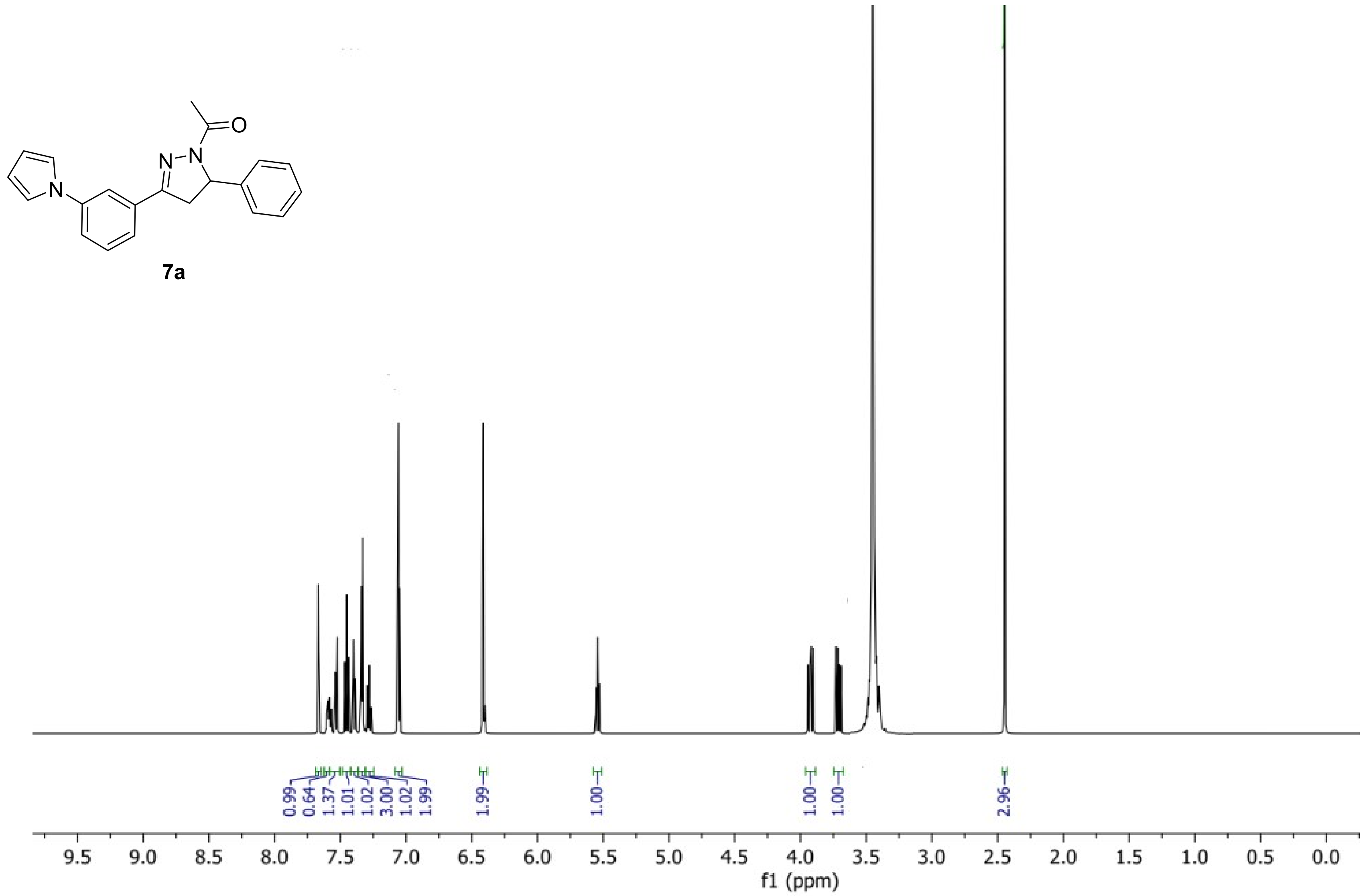

N
N
N
O
7a
0.99
0.64
1.37
1.01
1.02
3.00
1.02
1.99
1.99
1.00
1.00
1.00
2.96
9.5 9.0 8.5 8.0 7.5 7.0 6.5 6.0 5.5 5.0 4.5 4.0 3.5 3.0 2.5 2.0 1.5 1.0 0.5 0.0
f1 (ppm)

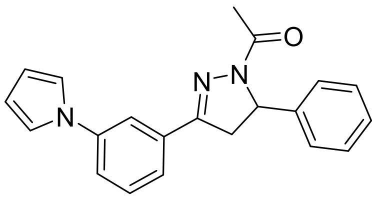

7a

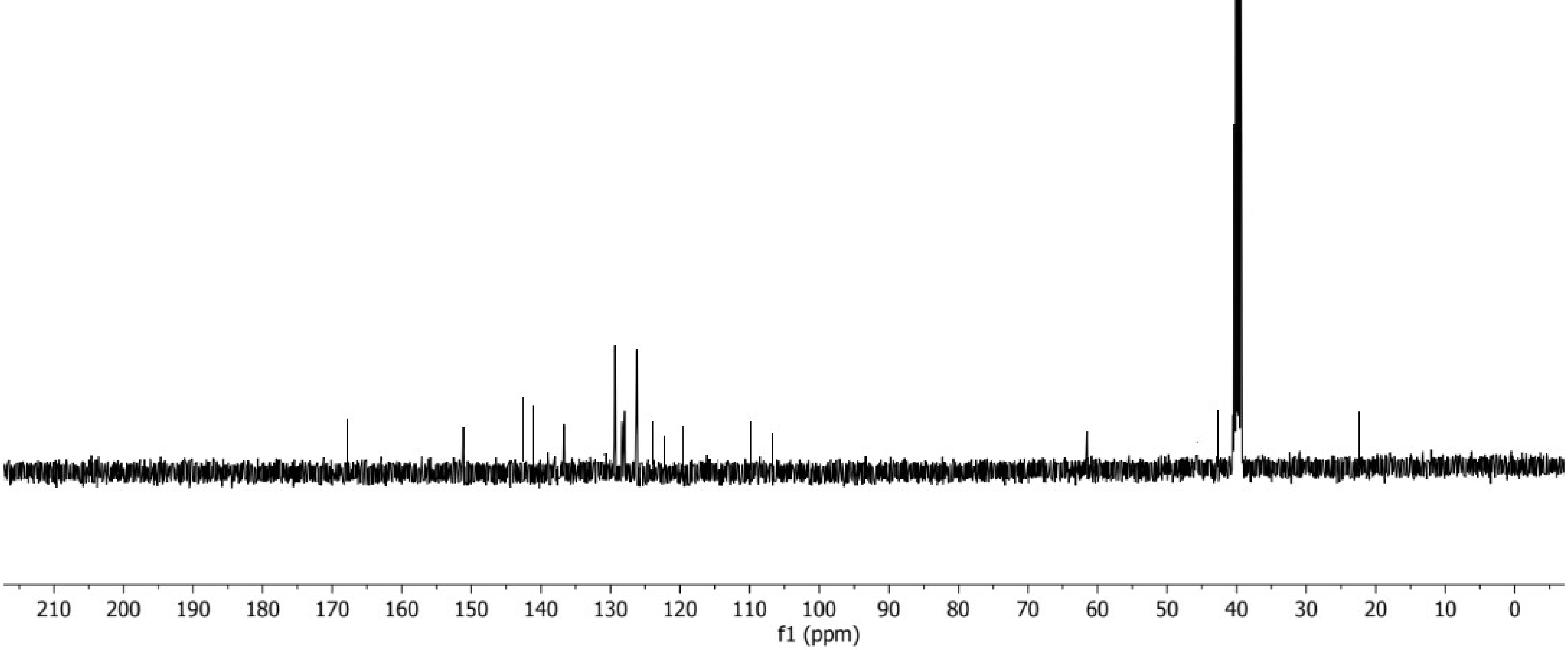
210 200 190 180 170 160 150 140 130 120 110 100 90 80 70 60 50 40 30 20 10 0
f1 (ppm)

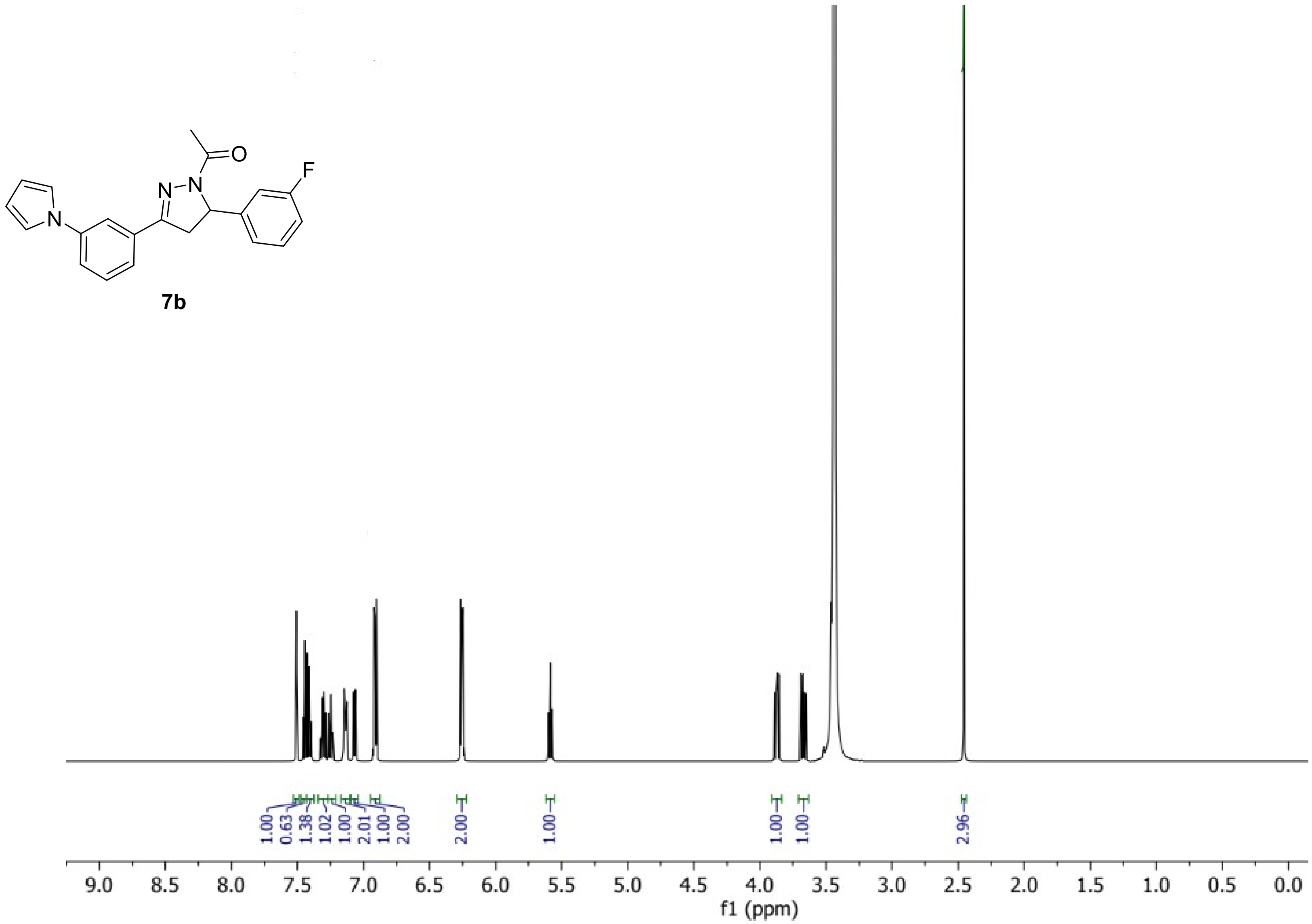

O
F
N
N
N
7b
1.00
0.63
1.38
1.02
1.00
2.01
1.00
2.00
2.00
1.00
1.00
1.00
2.96
9.0 8.5 8.0 7.5 7.0 6.5 6.0 5.5 5.0 4.5 4.0 3.5 3.0 2.5 2.0 1.5 1.0 0.5 0.0
f1 (ppm)

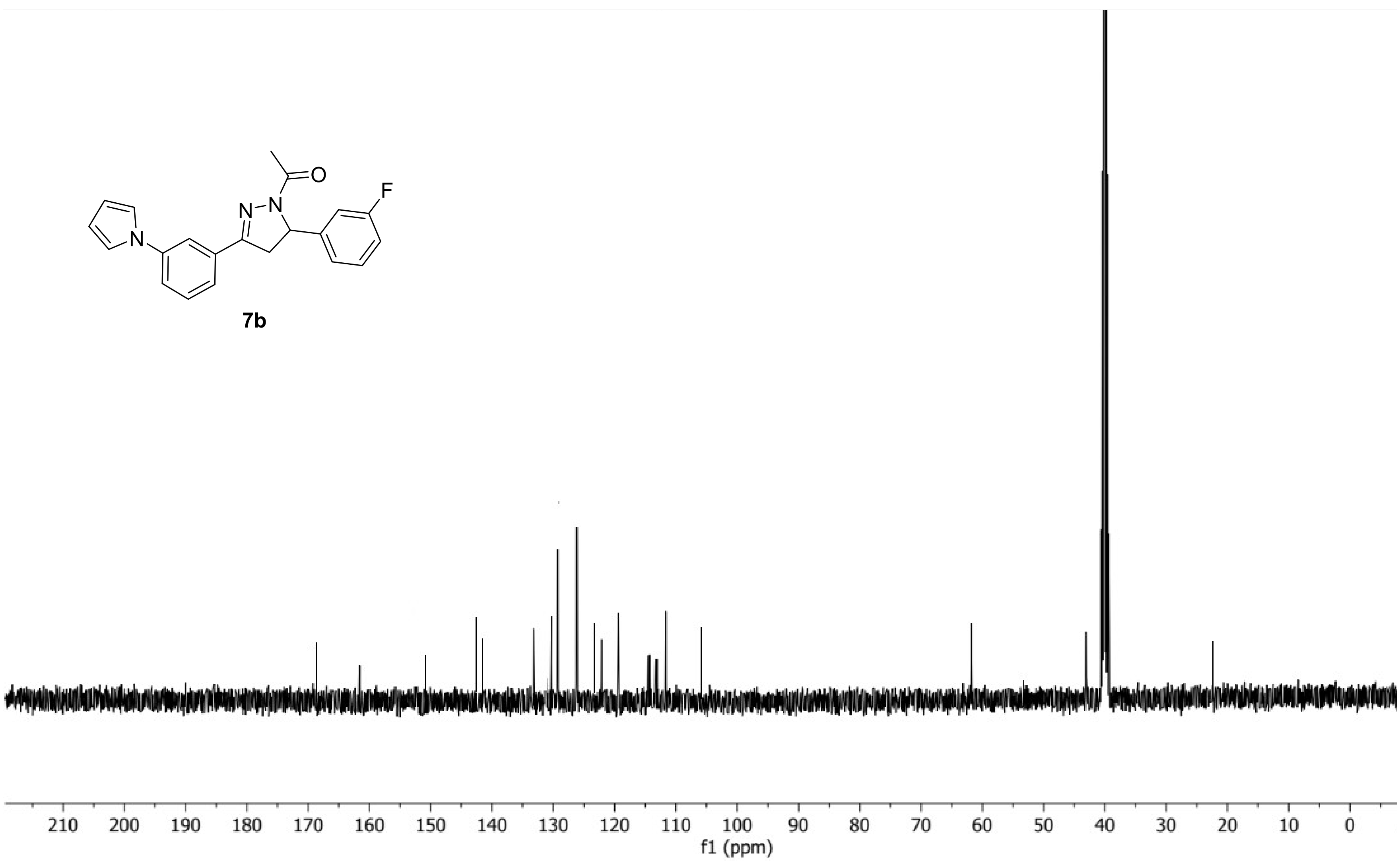
O
F
N
N
N

7b

210 200 190 180 170 160 150 140 130 120 110 100 90 80 70 60 50 40 30 20 10 0
f1 (ppm)

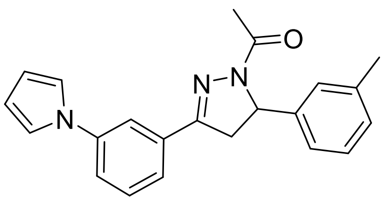
O
N–N
N

7c

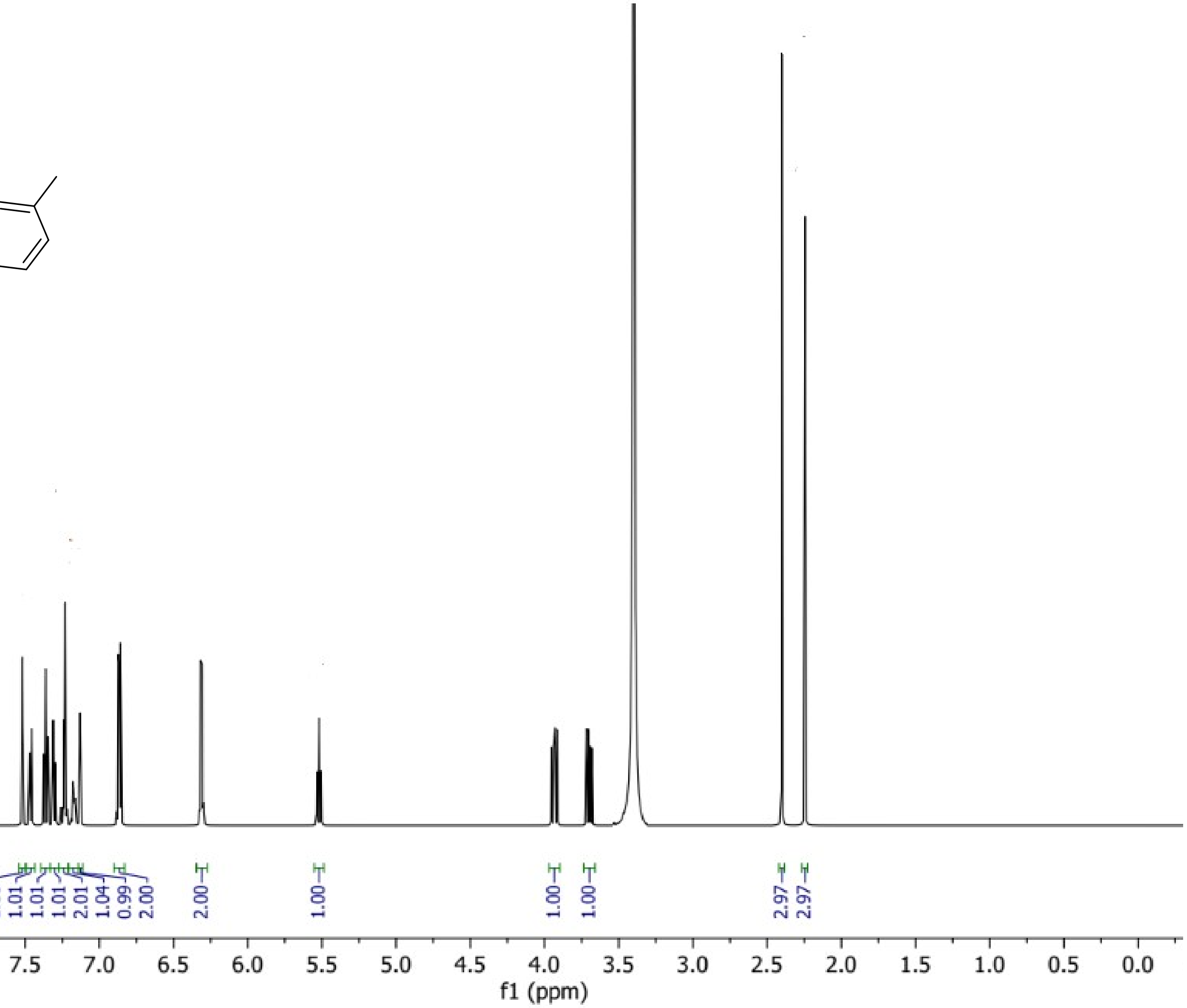
1.00
1.01
1.01
1.01
2.01
1.04
0.99
2.00
2.00
1.00
1.00
1.00
2.97
2.97
9.5 9.0 8.5 8.0 7.5 7.0 6.5 6.0 5.5 5.0 4.5 4.0 3.5 3.0 2.5 2.0 1.5 1.0 0.5 0.0
f1 (ppm)

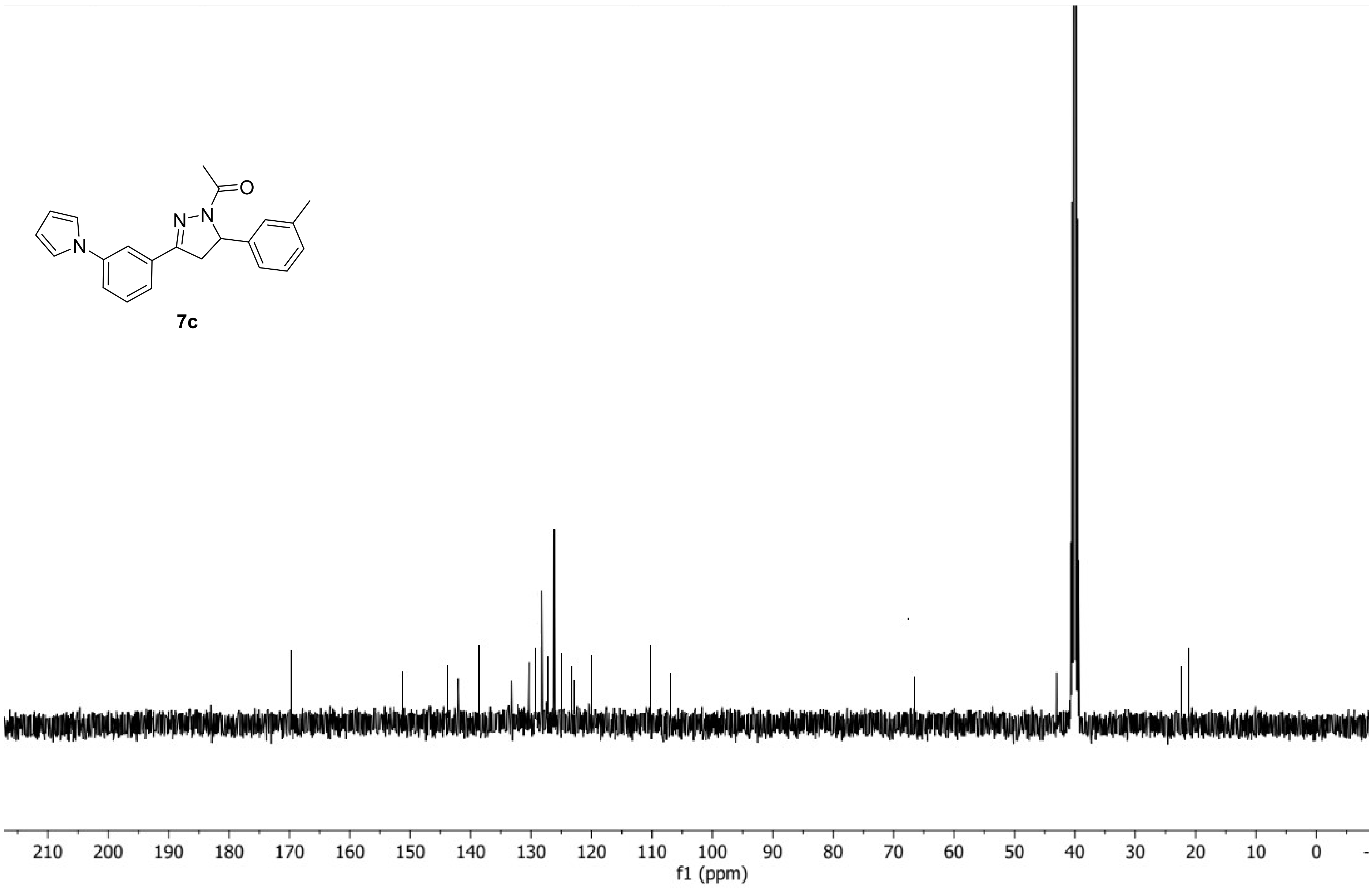

7c
210 200 190 180 170 160 150 140 130 120 110 100 90 80 70 60 50 40 30 20 10 0
f1 (ppm)

High-resolution mass spectrometry (HRMS) data for 6b

Elemental Composition Report

Page 1

Single Mass Analysis

Tolerance = 20.0 PPM / DBE: min = -1.5, max = 50.0
Element prediction: Off
Number of isotope peaks used for i-FIT = 5

Monoisotopic Mass, Even Electron Ions
51 formula(e) evaluated with 2 results within limits (up to 50 closest results for each mass)
Elements Used:
C: 1-300 H: 0-600 N: 0-4 F:0-1 Na: 0-1
MA_0227_rr 9 (0.203) Cm (9:12)
1: TOF MS ES+

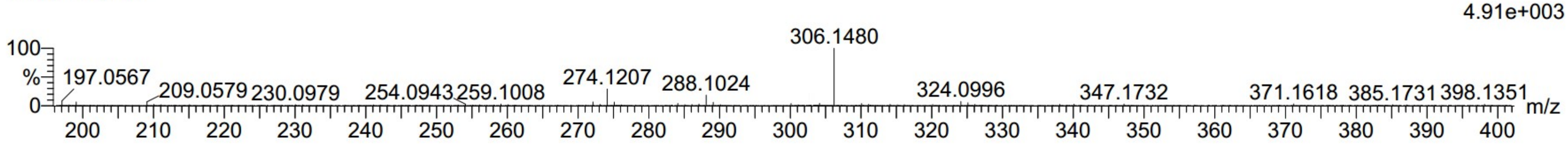

Minimum:				-1.5			
Maximum:		5000.0	20.0	50.0			

Exact Mass	Calc. Mass	mDa	PPM	DBE	i-FIT	i-FIT (Norm)	Formula
306.1480	306.1407	-0.1	-0.3	13	372.2	0.0	C19 H17 N3 F
